# Global, regional, and national burden of malignant neoplasm of bone and articular cartilage in adults aged 65 years and older, 1990–2021: a systematic analysis based on the global burden of disease study 2021

**DOI:** 10.1007/s40520-024-02926-0

**Published:** 2025-01-08

**Authors:** Xiang Zhang, Xiao Dai, Yuelin Chen, Song Wang, Hao Yang, Bo Qu, Hong Luo, Hongsheng Yang

**Affiliations:** 1 Orthopedics Department of the First Affiliated Hospital of Chengdu Medical College, Chengdu, China; 2 Department of Orthopedics, The Affiliated Hospital of Southwest Medical University, Luzhou, 646000 China; 3 Department of Respiratory and Critical Care Medicine, West China Hospital and, Division of Pulmonary Diseases, State Key Laboratory of Biotherapy of China, Sichuan University, Chengdu, Sichuan China

**Keywords:** Malignant neoplasm of bone and articular cartilage, Mortality, Disability-adjusted life-years (DALYs), Global Burden of Disease, Sociodemographic index

## Abstract

**Background:**

This study aims to delineate the global, regional, and national burden of malignant neoplasms of bone and articular cartilage (MNBAC) among individuals aged 65 years and older from 1990 to 2021, stratified by age, sex, and sociodemographic index (SDI).

**Methods:**

We harnessed data from the Global Burden of Disease Study 2021 to evaluate the prevalence, incidence, mortality, and disability-adjusted life years (DALYs) associated with MNBAC among individuals aged 65 years and older across 204 countries and territories between 1990 and 2021. The socio-demographic Index (SDI) served as a metric to examine the influence of socioeconomic development on the burden of MNBAC. Furthermore, joinpoint regression analysis was employed to identify the years marked by the most significant temporal changes over the study period.

**Results:**

In 2021, an estimated 163,561 prevalent cases of MNBAC were recorded among individuals aged ≥ 65 years, alongside 28,100 newly diagnosed cases, 27,588 deaths, and 508,202 DALYs. The age-standardized rates per 100,000 population were 21.30 for prevalence, 3.69 for incidence, 3.66 for mortality, and 65.85 for DALYs. Notably, Cuba reported the highest prevalence rate (42.42), while the Philippines exhibited the greatest DALY burden (161.78). Egypt demonstrated the highest incidence (7.44) and mortality rates (8.90). A significant inverse correlation was observed between age-standardized DALY rates and SDI across regions.

**Conclusions:**

This analysis underscores the substantial global burden of MNBAC among older adults, accentuating the imperative for tailored public health interventions, alongside advancements in diagnostic and therapeutic approaches, particularly within resource-constrained settings.

**Supplementary Information:**

The online version contains supplementary material available at 10.1007/s40520-024-02926-0.

## Introduction

Primary bone tumors are exceedingly rare, constituting only 0.2% of all human neoplasms [1]. As per the most recent data from 2020, the United States reported 3,600 newly diagnosed cases of primary malignant bone tumors, with 1,720 deaths attributed to these malignancies [2]. Notably, the burden of malignant neoplasms of bone and articular cartilage (MNBAC) in the elderly population demands focused attention. With the global demographic shift towards an aging population, the incidence of these tumors is anticipated to rise, presenting considerable public health challenges. Among adults, chondrosarcoma emerges as the most common subtype, accounting for approximately 40% of cases [3]. MNBAC in geriatric individuals encompass a heterogeneous group of malignant disorders, including osteosarcoma and chondrosarcoma, defined by the unregulated proliferation of cells originating from the skeletal system [4]. This demographic is particularly susceptible due to multiple compounding factors, such as the prevalence of comorbid conditions, diminished bone regeneration capacity, and age-related alterations in bone metabolism, all of which complicate both the diagnosis and management of malignant bone tumors. Furthermore, older adults often encounter distinctive challenges in oncologic treatment, including reduced tolerance to intensive therapeutic regimens and an elevated risk of treatment-associated complications. These factors collectively contribute to poorer prognoses and higher mortality rates in elderly patients compared to their younger counterparts. Despite advancements in diagnostic modalities and therapeutic strategies, the burden of these malignancies in older adults remains significant, exerting a profound influence on clinical outcomes [4]. Existing studies have documented variations in the incidence and mortality rates of MNBAC across different regions and age demographics [5–8]. However, most research efforts have been geographically or nationally confined. While these studies have yielded valuable insights, the lack of standardized methodologies for assessing prevalence has limited cross-regional and international comparisons. As a result, a comprehensive global perspective on the epidemiology of MNBAC and its specific implications for the elderly population remains inadequately addressed.

The Global Burden of Disease Study (GBD) constitutes a transformative undertaking in the field of comprehensive health evaluation, systematically quantifying the burden imposed by a vast spectrum of diseases, injuries, and risk factors at both global and regional levels [9]. This collaborative initiative, uniting expertise from an extensive network of research institutions and scholars worldwide, generates intricate and granular data on critical health indicators, such as incidence, mortality, and disability-adjusted life years (DALYs). Employing advanced analytical methodologies and exhaustive systematic reviews, the GBD integrates epidemiological evidence from a broad array of nations and regions. As an indispensable tool, it underpins evidence-informed public health policymaking, equitable allocation of healthcare resources, and meticulous assessment of health interventions on a worldwide scale [10].

The primary objective of this study is to delineate the temporal trends in incidence, prevalence, mortality, and DALYs attributable to MNBAC among individuals aged 65 years and older, seeking to generate critical insights to guide and enhance preventive strategies and therapeutic approaches for MNBAC in this demographic, with a specific focus on addressing the challenges prevalent in resource-constrained settings.

## Material and methods

### Data source and diagnostic criteria

This study utilized data from the GBD 2021, which provides comprehensive estimates of disease burden at global, regional, and national levels. Curated by the Institute for Health Metrics and Evaluation, the GBD 2021 employs a standardized methodology to estimate incidence, prevalence, mortality, and DALYs across diseases and injuries, encompassing 204 countries and territories [11]. Data on MNBAC were extracted using the GBD Results Tool, including age-standardized rates for incidence (ASIR), prevalence (ASPR), mortality (ASMR), and DALYs (ASDR), as well as global and regional estimates from 1990 to 2021. To ensure comparability across populations with age structures over time and across geographical locations, all measures were standardized to the GBD 2021 global population. Over the study period, diagnostic criteria for MNBAC have undergone substantial evolution, driven by advancements in medical imaging, histopathological techniques, and molecular diagnostics. For example, the integration of increasingly sensitive and specific biomarkers has facilitated earlier detection and more precise classification of malignant neoplasms. These diagnostic advancements, while improving accuracy, have introduced challenges to data consistency, complicating historical comparisons. To mitigate these challenges, the GBD framework applies standardized definitions and classifications aligned with contemporary diagnostic criteria to harmonize data across temporal and geographical contexts.

### DALY calculation

The calculation of DALYs in this context entails the aggregation of years of life lost (YLLs) due to premature mortality and years lived with disability (YLDs). YLLs are determined by multiplying the number of deaths by the standard life expectancy corresponding to the age at death [12]. YLDs, on the other hand, are computed by multiplying the prevalence of each condition by a disability weight, which quantifies the severity of health loss attributable to the condition [11]. The GBD study employs a standardized methodology to ensure the comparability of DALY estimates across regions and temporal intervals, accounting for variations in healthcare infrastructure and access to medical services that may influence this measure.

### Sociodemographic index

The sociodemographic index (SDI), an advanced composite metric formulated within the Global Burden of Disease (GBD) framework, functions as a critical indicator for assessing the socioeconomic development status of countries and regions. This multidimensional measure integrates three core components: per capita income, average educational attainment among individuals aged 15 years and older, and the total fertility rate among women under the age of 25 [13]. Scaled from 0 to 1, with higher values representing greater socioeconomic advancement, the SDI provides a standardized benchmark for comparative analyses across varied geographical and temporal contexts, enriching our understanding of the complex interrelations between socioeconomic factors and health outcomes, thereby advancing global health research.

### Statistical analysis

To evaluate temporal trends over the study period, we calculated both the absolute and estimated annual percentage change (EAPC) for each metric from 1990 to 2021. Age-standardized rates, along with their corresponding 95% certainty intervals (CI), were estimated using the GBD standard population framework, with rates expressed per 100,000 population [13]. Advanced statistical techniques, including EAPC analysis [14], Bayesian Age-Period-Cohort (BAPC) modeling [15] and joinpoint regression analysis [16], were employed to identify trends and underlying drivers of disease burden. A detailed description of these methodological approaches is provided in Supplementary Table S1. All analyses and visualizations were executed using Joinpoint Regression Program (version 4.9.0.0) and R software (V.4.3.1) [17].

## Results

### Global trends

In 2021, the global prevalence of MNBAC among individuals aged ≥ 65 years was estimated at 163,561.30 cases, with an ASPR of 21.30, reflecting a slight increase since 1990, as evidenced by an EAPC of 0.85 (95% CI 0.75, 0.95) (Figure S1 and Table S2). Joinpoint regression analysis identified significant inflection points in the ASPR of MNBAC within this age group in 2001, 2005, 2013, and 2018 (Figure S2A). Globally, the incidence of MNBAC in individuals aged ≥ 65 years reached 28,100.56 new cases in 2021, with an ASIR of 3.69, indicating a modest increase from 1990, with an EAPC of 0.64 (95% CI 0.55–0.73) (Figure S2 and Table S3). Significant shifts in ASIR were similarly detected in 2001, 2005, 2013, and 2018 through joinpoint regression analysis (Figure S2B). Mortality associated with MNBAC in the same age group amounted to 27,588.16 deaths in 2021, with an ASMR of 3.66, representing a slight rise compared to 1990, with an EAPC of 0.22 (95% CI 0.11–0.34) (Figure S3 and Table S4). Joinpoint regression analysis revealed significant changes in ASMR in 1996, 2000, 2005, 2013, and 2017 (Figure S2C). Additionally, the global burden of MNBAC in individuals aged ≥ 65 years, measured in DALYs, was 508,202.61 in 2021, with an ASDR of 65.85, indicating a slight increase since 1990, with an EAPC of 0.21 (95% CI: 0.10–0.33) (Figure S4 and Table S5). Significant inflection points in ASDR were observed in 1995, 2000, 2005, 2013, and 2017 according to joinpoint regression analysis (Figure S2D). Significant inflection points in ASDR were observed in 1995, 2000, 2005, 2013, and 2017 according to joinpoint regression analysis (Figure S2D).

### Regional discrepancies

In 2021, the highest ASPRs of MNBAC among individuals aged ≥ 65 years were recorded in East Asia (34.28), the Caribbean (28.90), and Southeast Asia (28.67), while the lowest prevalence rates were observed in High-income Asia Pacific (6.04), Oceania (8.91), and Australasia (10.01) (Table S2). The ASIRs of MNBAC in this age group were notably elevated in East Asia (5.96), Southeast Asia (5.00), and the Caribbean (4.96), whereas the lowest rates were reported in High-income Asia Pacific (0.98), Oceania (1.63), and Australasia (1.67) (Table S3). Similarly, the ASMRs were highest in Southeast Asia (5.73), East Asia (5.53), and the Caribbean (5.40), with the lowest mortality rates found in High-income Asia Pacific (0.75), Australasia (1.28), and Eastern Europe (1.83) (Table S4). Regarding the burden of disease, the ASDRs for MNBAC among individuals aged ≥ 65 years peaked in Southeast Asia (105.23), East Asia (98.55), and the Caribbean (95.30), while the lowest DALY rates were documented in High-income Asia Pacific (13.12), Australasia (21.42), and High-income North America (31.92) (Table S5).

The prevalence of MNBAC among individuals aged ≥ 65 years surged from 56,527.41 cases in 1990 to 163,561.30 cases in 2021. In 2021, the regions with the highest prevalence were East Asia (69,378.73), South Asia (18,112.57), and Southeast Asia (14,663.50) (Table S2). During the same period, the number of incident MNBAC cases in this age group increased from 10,112.65 in 1990 to 28,100.56 in 2021, with East Asia accounting for the highest incidence (11,762.22), followed by South Asia (3,193.68) and Southeast Asia (2,509.23) (Table S3). Mortality attributable to MNBAC in people aged ≥ 65 years also rose substantially, from 11,115.53 deaths in 1990 to 27,588.16 deaths in 2021, with the greatest number of deaths recorded in East Asia (10,630.10), South Asia (3,706.42), and Southeast Asia (2,811.64) (Table S4). Furthermore, the total DALYs associated with MNBAC in this population increased significantly, from 209,290.95 in 1990 to 508,202.61 in 2021, with East Asia (199,532.65), South Asia (70,675.73), and Southeast Asia (54,678.38) bearing the highest burden (Table S5).

### National variations

**Fig. 1:**
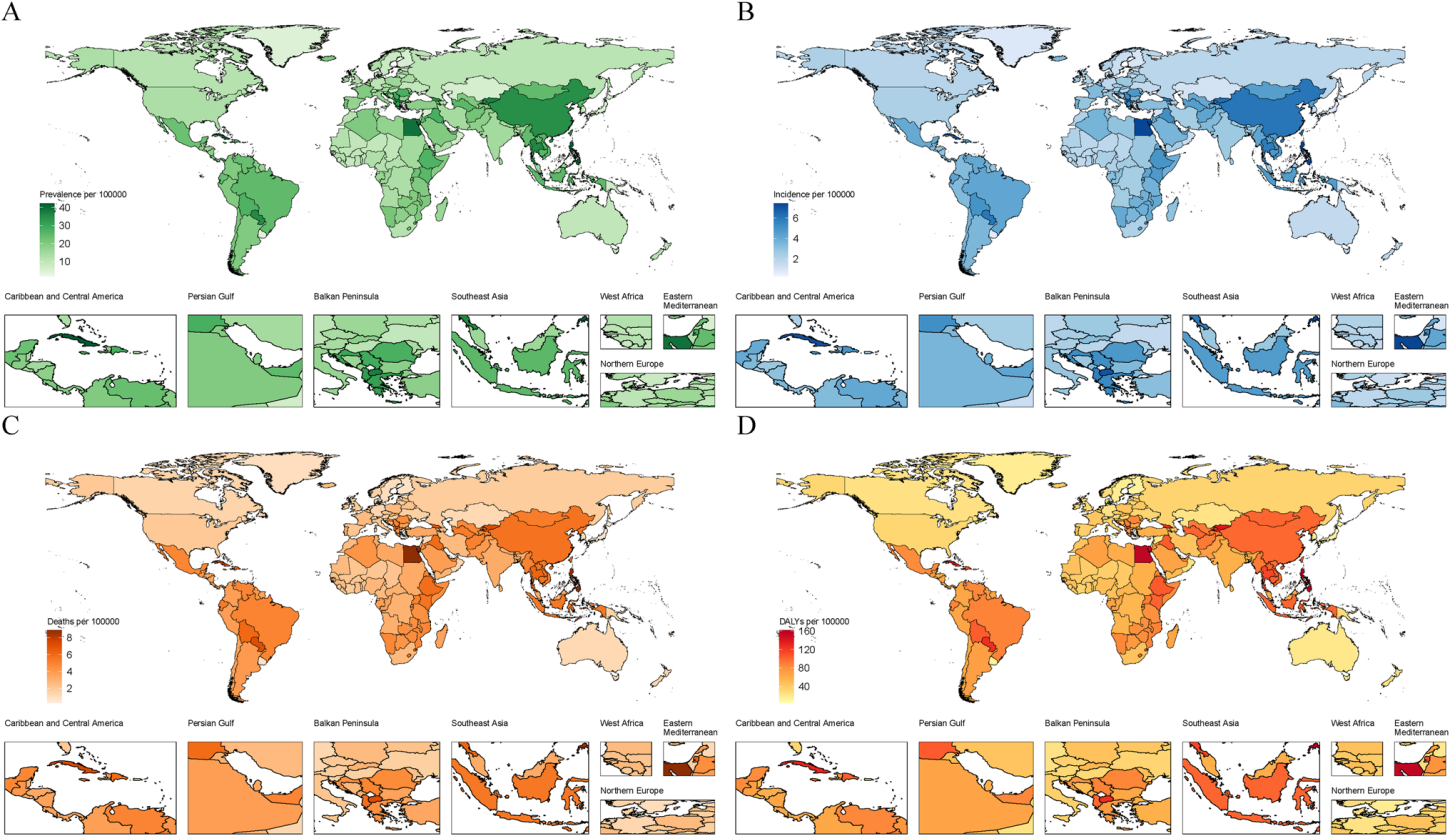
Age-standardized rates of prevalence (**A**), incidence (**B**), mortality (**C**), and disability adjusted life years (DALYs) (**D**) for malignant neoplasms of bone and articular cartilage in people aged ≥ 65 years across 204 countries in 2021

In 2021, the national ASPRs of MNBAC among individuals aged ≥ 65 years ranged from 1.93 to 42.42 cases. The highest ASPRs were observed in Cuba (42.42), the Philippines (41.97), and Egypt (39.13), while the lowest estimates were recorded in Palau (1.93), Andorra (2.00), and Monaco (2.57) (Fig. 1A and Table S2). Between 1990 and 2021, the EAPC in ASPRs exhibited significant variability across countries, with China (3.86), Saint Vincent and the Grenadines (3.48), and Thailand (2.76) demonstrating the most pronounced increases. In contrast, the Russian Federation (5.10), Greece (3.50), and the Republic of Moldova (2.96) experienced the steepest declines.

In 2021, the national ASIRs for MNBAC among individuals aged ≥ 65 years ranged from 0.34 to 7.44 cases. The highest rates were recorded in Egypt (7.44), the Philippines (7.42), and Cuba (7.19), while the lowest rates were observed in Andorra (0.34), Palau (0.34), and Monaco (0.44) (Fig. 1B and Table S3). Between 1990 and 2021, the most substantial increases in ASIRs were noted in China (3.55), Saint Vincent and the Grenadines (3.38), and Thailand (2.43). Conversely, the Russian Federation (5.32), Greece (3.63), and the Republic of Moldova (3.31) exhibited the most significant declines.

In 2021, the national ASMRs for MNBAC among individuals aged ≥ 65 years ranged from 0.05 to 1.77 deaths. The highest ASMRs were reported in Egypt (8.90), the Philippines (8.72), and Saint Vincent and the Grenadines (7.50), while the lowest rates were observed in Andorra (0.26), Monaco (0.34), and Palau (0.40) (Fig. 1C and Table S4). Between 1990 and 2021, the most notable increases in ASMRs occurred in Saint Vincent and the Grenadines (3.41), China (2.61), and Sri Lanka (2.11). In contrast, the Russian Federation (5.95), Greece (3.95), and the Republic of Moldova (3.73) experienced the most pronounced declines.

In 2021, the national ASDRs for MNBAC among individuals aged ≥ 65 years ranged from 4.34 to 161.78. The highest rates were recorded in the Philippines (161.78), Egypt (154.32), and Kyrgyzstan (139.71), while the lowest were observed in Andorra (4.34), Monaco (5.75), and Palau (7.25) (Fig. 1D and Table S5). Between 1990 and 2021, the most significant increases in ASDRs were noted in Saint Vincent and the Grenadines (3.31), China (2.59), and Thailand (2.11). Conversely, the largest reductions during this period were reported in the Russian Federation (6.00), Greece (4.02), and Finland (3.57).

### Age trends and gender disparities

**Fig. 2:**
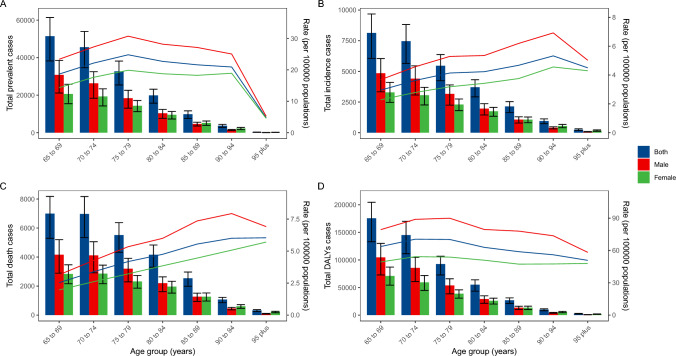
Total cases and rates of prevalence (**A**), incidence (**B**), mortality (**C**), and disability adjusted life years (DALYs) (**D**) of malignant neoplasms of bone and articular cartilage in people aged ≥ 65 years across gender and age groups in 2021. Error bars indicate the 95% certainty interval for numbers

In 2021, the global prevalence of MNBAC among individuals aged ≥ 65 years was highest in the 75–79 age group, while the total number of cases peaked in the 65–69 age group. Across all age groups, males consistently exhibited a higher prevalence of MNBAC compared to females (Fig. 2A). Similarly, the incidence of MNBAC among individuals aged ≥ 65 years reached its apex in the 90–94 age group. The distribution of the total number of incident cases mirrored that of prevalence, with males displaying higher incidence rates across all age groups (Fig. 2B). The mortality pattern of MNBAC in 2021 closely followed the trend observed for incidence (Fig. 2C). Additionally, the DALYs rate for MNBAC among individuals aged ≥ 65 years peaked in the 75–79 age group before declining thereafter. The trend in the total number of DALYs paralleled the pattern observed in the rate (Fig. 2D).

### Correlation with the sociodemographic index

**Fig. 3:**
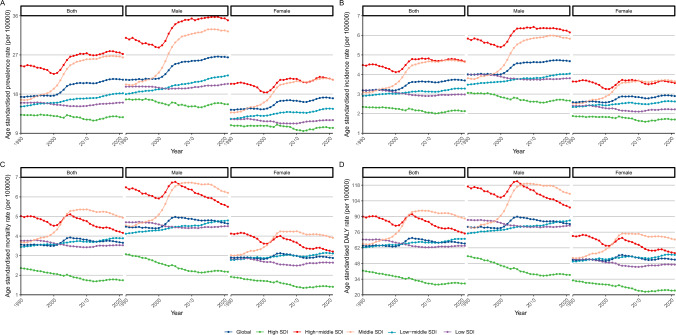
Trends analyses of age-standardized rates of prevalence (**A**), incidence (**B**), mortality (**C**), and disability adjusted life years (DALYs) (**D**) for malignant neoplasms of bone and articular cartilage in people aged ≥ 65 years by gender and sociodemographic index from 1990 to 2021

From 1990 to 2021, ASPRs in high-middle SDI regions consistently exceeded those observed in other areas. Throughout this period, female prevalence rates remained uniformly lower than those of males, both regionally and globally (Fig. 3A). The ASIRs of MNBAC among individuals aged ≥ 65 years mirrored the global and regional prevalence trends during the same timeframe, with high-middle SDI regions exhibiting notably elevated incidence rates compared to other areas. Across all SDI regions, female incidence rates were consistently lower than those observed in males (Fig. 3B). High SDI regions, meanwhile, sustained the lowest ASMRs relative to other regions throughout the study period (Fig. 3C). Similarly, the DALY rates in high SDI regions were consistently the lowest compared to other regions during this timeframe (Fig. 3D). These findings underscore the intricate interplay between sociodemographic factors and gender disparities in the epidemiology of MNBAC among individuals aged ≥ 65 years across varying regions and over time.

**Fig. 4:**
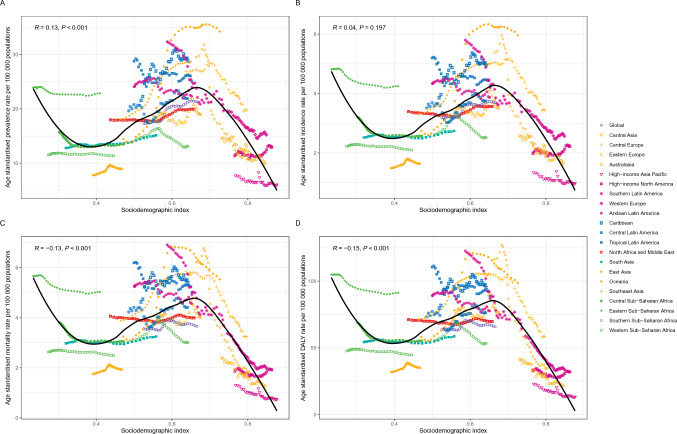
Age-standardized rate of prevalence (**A**), incidence (**B**), mortality (**C**), and disability adjusted life years (DALYs) (**D**) due to malignant neoplasms of bone and articular cartilage in people aged ≥ 65 years by GBD regions and the expected value based on the SDI from1990 to 2021

In the regional analysis from 1990 to 2021, a negative correlation was identified between the ASDR for MNBAC among individuals aged 65 years and older and the SDI. Throughout this period, the Eastern Sub-Saharan Africa region exhibited an ASDR that was unexpectedly elevated in relation to its SDI. Conversely, regions such as Western Sub-Saharan Africa, Oceania, and East Asia experienced a disease burden that was lower than anticipated (Fig. 4D). The correlation between the ASMRs for MNBAC in the elderly population and SDI was consistent with that of the DALY rate. From 1990 to 2021, regions including Eastern Sub-Saharan Africa, Central Europe, and Southeast Asia recorded mortality rates that surpassed those projected by their respective SDIs. In contrast, areas such as Oceania and Western Sub-Saharan Africa exhibited a disease burden that was lower than expected (Fig. 4C). The relationship between the ASPR and ASIR for MNBAC in individuals aged 65 years and older paralleled that of the DALY rate (Fig. 4A and Fig. 4B). At the national level in 2021, an increase in SDI corresponded with a decline in the age-standardized DALY rate, prevalence rate, mortality rate, and incidence rate (Figure S3).

### Future forecasts of global burden of MNBAC in people aged ≥ 65 years

**Fig. 5:**
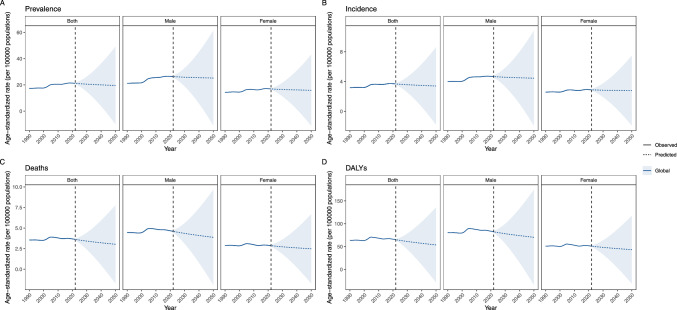
Future forecasts of global burden of age-standardized rates of prevalence (**A**), incidence (**B**), mortality (**C**), and disability adjusted life years (DALYs) (**D**) by 2050 of MNBAC in people aged ≥ 65 years

It is anticipated that by 2050, the ASMR and ASIR will stabilize. Conversely, the ASDR and ASPR for MNBAC among individuals aged 65 years and older will undergo a gradual decline on a global scale (Fig. 5).

## Discussion

This study provides a comprehensive analysis of the incidence, prevalence, mortality, and DALYs associated with MNBAC among individuals aged 65 years and older from 1990 to 2021, along with age-standardized rates across 204 countries and territories. In 2021, the global burden of MNBAC included 598,637 prevalent cases, 91,375 new cases, 66,114 deaths, and 2,525,827 DALYs.

The global prevalence and incidence trends of MNBAC among individuals aged ≥ 65 years exhibit marked heterogeneity across regions and populations. For instance, in South American countries such as Colombia and Ecuador, young males show a higher incidence rate of 7 to 7.6 cases per million [6, 7]. In the United States, Black individuals have an incidence rate of 5.1 cases per million [18]. Among Latin American populations, a pronounced gender disparity is evident, with males exhibiting an incidence rate of 7.0–7.6 cases per million, compared to a lower rate of 3.5–4.9 cases per million among females [18]. Notably, the Philippines reports an exceptionally elevated incidence rate of 11.4 cases per million, surpassing global averages [6, 19]. These findings diverge from the results of our study, likely due to differences in the study periods and the diagnostic criteria applied. As such, the burden of MNBAC among individuals aged ≥ 65 years, as estimated in our analysis, cannot be directly compared to findings from individual studies, which highlights the necessity of accounting for genetic, environmental, and socioeconomic factors in understanding the etiology and epidemiology of MNBAC.

Our findings indicate that, in 2021, the Philippines reported the highest global age-standardized incidence and mortality rates of MNBAC among individuals aged ≥ 65 years, reaching 125.8 and 17.7 per million population, respectively. Epidemiological estimates suggest an annual incidence of osteosarcoma in the Philippines ranging from 200 to 300 cases [20]. Alarmingly, the two-year overall survival rate for osteosarcoma in the country remains at 50%, while the treatment abandonment rate is as high as 36%, paralleling figures observed in other low- and middle-income nations [21–24]. The lack of coordinated, multidisciplinary treatment approaches has been identified as a key driver of treatment abandonment [22, 25]. Furthermore, economic constraints likely exacerbate this issue. Comparative data on healthcare expenditures highlight that out-of-pocket health spending constitutes 54% of total health expenditures in the Philippines, markedly surpassing the proportions in Singapore (31%) and Thailand (11%), based on 2021 World Bank statistics [26]. A retrospective multicenter study revealed that Filipino patients, compared to those in Thailand and Singapore, were diagnosed at a higher median age, with larger tumors, and were less likely to receive neoadjuvant chemotherapy. Moreover, Filipino patients presented with a higher incidence of metastatic disease at diagnosis [26]. These factors collectively contribute to the elevated mortality burden in the Philippines. Similarly, in Egypt, a substantial segment of the population resides in socioeconomically disadvantaged regions with severely restricted access to healthcare services. Consequently, many patients present at late stages of disease, resulting in a higher proportion of metastatic cases at initial diagnosis [27].

Our findings indicate that MNBAC among individuals aged ≥ 65 years demonstrates a peak incidence in the 65–69 age group, a trend consistent with prior epidemiological research [28]. In this population, MNBAC often arises in previously irradiated regions or as a secondary malignancy associated with underlying conditions such as Paget's disease of bone [29]. Among elderly patients, osteosarcoma is more frequently localized to axial skeletal sites or regions with prior radiation exposure or pre-existing bone abnormalities, influenced by a combination of factors [30]. With advancing age, the diminished capacity for DNA repair and the accumulation of cell division errors may lead to the inactivation of tumor suppressor genes or the activation of proto-oncogenes, thereby increasing susceptibility to malignancy [31]. Additionally, older adults are particularly prone to vitamin D deficiency, which can disrupt bone metabolism and exacerbate conditions such as osteoporosis and other degenerative skeletal changes, creating a microenvironment favorable to osteosarcoma development [32]. It is important to note that osteosarcoma in this demographic is frequently secondary in origin, often emerging from benign bone lesions, including those associated with Paget's disease of bone[33].

Multiple studies have consistently identified a gender disparity in the incidence of osteosarcoma, with a higher prevalence observed among males, reflected in an average male-to-female ratio of 1.4:1 [34]. Evidence suggests that pubertal skeletal growth, coupled with hormonal fluctuations and developmental processes, may play a pivotal role in the pathogenesis of osteosarcoma [19, 35]. This gender-specific pattern, alongside the distinct age distribution, underscores the potential influence of sex-specific biological factors and growth-related mechanisms in the etiology of osteosarcoma, necessitating further exploration of the pathways contributing to these disparities.

Our findings reveal a strong correlation between the SDI and mortality rates among osteosarcoma patients. Socioeconomic status, encompassing factors such as education, income, and occupation, has been identified as a significant predictor of disease incidence and mortality, with education exerting the most pronounced influence on patient survival. Individuals with limited or no formal education may face challenges in comprehending the severity of their condition, potentially resulting in delays or refusals to seek timely medical care [19]. Epidemiological studies further demonstrate that patients from socioeconomically disadvantaged regions are more likely to present with advanced disease, including larger tumor sizes and higher rates of metastasis [36, 37]. In the United States, socioeconomic factors have been definitively linked to overall survival disparities, with lower socioeconomic status correlating with poorer survival outcomes among osteosarcoma patients [38]. These findings highlight the pivotal role of social determinants in shaping health outcomes and underscore the pressing need for targeted interventions to mitigate disparities in osteosarcoma care and survival across socioeconomic groups.

To reduce disparities in osteosarcoma outcomes, particularly in low-and middle-income countries (LMICs) where mortality and DALY rates remain disproportionately high, the implementation of targeted public health strategies is imperative. First, enhancing early detection through community-based screening initiatives, particularly in underserved rural areas, is essential. Public awareness campaigns tailored to local cultural and socioeconomic contexts should aim to educate communities about the symptoms of osteosarcoma and the critical importance of early intervention. Second, improving access to treatment must be prioritized. Patients in LMICs frequently face significant barriers, including financial hardship and inadequate healthcare infrastructure. National policies should focus on subsidizing cancer care and strengthening referral pathways to ensure timely access to specialized services. Collaboration with international organizations can further support these efforts by providing resources, training, and capacity-building for local healthcare providers. Third, investment in cost-effective and accessible diagnostic technologies is vital. The absence of advanced diagnostic tools in many LMICs often results in delayed diagnoses and poorer outcomes. Partnerships between high-SDI and low-SDI countries can play a transformative role by offering technical expertise, financial assistance, and the adaptation of proven strategies from regions with lower mortality rates. Finally, fostering sustained international collaboration is critical to addressing disparities. High-income countries can support LMICs by sharing best practices, establishing cancer registries, implementing standardized treatment protocols, and developing sustainable healthcare infrastructure. In summary, tackling osteosarcoma in resource-limited settings necessitates a multifaceted approach that includes early detection, expanded treatment access, investment in diagnostics, and robust international partnerships. Leveraging successful models from high-SDI regions offers a pathway to reducing mortality and DALY rates in LMICs.

Projections suggest a decline in the ASDR and ASMR for MNBAC among individuals aged ≥ 65 years by 2050. This anticipated reduction underscores the importance of critically evaluating its implications for global health policy and shaping future research priorities, which is likely attributable to advancements in therapeutic interventions, improved preventive strategies, and strengthened healthcare infrastructures. To effectively address this trend, healthcare systems must proactively allocate resources and target investments in regions with the highest disease burden, particularly those with elevated DALY rates, to alleviate the global impact. Furthermore, reducing disparities in MNBAC prevalence and outcomes across nations remains a pressing priority. Policymakers in heavily affected regions should prioritize comprehensive epidemiological studies to better understand disease patterns and risk factors. Future research efforts should focus on developing targeted therapies tailored to the needs of older populations, while also leveraging genetic and molecular profiling to identify high-risk individuals. Aligning healthcare strategies with these emerging trends and insights will be essential to addressing the evolving challenges of MNBAC in aging populations.

While this study offers a comprehensive analysis of the epidemiology of MNBAC in individuals aged ≥ 65 years at global, regional, and national levels over the period 1990–2021, several limitations warrant more detailed consideration. First, substantial variability exists in the quality and completeness of data across countries and regions. Low-income countries, in particular, often grapple with inadequate healthcare infrastructure and underdeveloped reporting systems, leading to significant underreporting of MNBAC cases. This underreporting likely results in an underestimation of the true burden of disease in these regions, potentially distorting the global and regional trends observed in this analysis. Second, shifts in diagnostic criteria and advancements in diagnostic technologies over the three-decade study period may have introduced inconsistencies in data collection and interpretation. For instance, the adoption of improved diagnostic tools and increased access to healthcare in high-income countries may have facilitated earlier and more accurate case detection. In contrast, such advancements have not been uniformly implemented in lower-income regions, complicating cross-regional comparisons. Consequently, observed trends may reflect disparities in diagnostic practices rather than genuine changes in disease incidence or burden. Additionally, the extended timeframe of the study (1990–2021) poses challenges in comparing regions with vastly different healthcare access and diagnostic capabilities. Variations in healthcare systems, surveillance infrastructure, and diagnostic technologies across countries and over time may have introduced biases, potentially leading to either overestimation or underestimation of the true burden of MNBAC in certain regions. Given these limitations, it is essential to interpret the findings with caution, particularly when assessing trends across regions with unequal healthcare resources and reporting systems. To address these challenges, there is an urgent need for standardized and consistent data collection methodologies across countries and regions. Strengthening surveillance systems, particularly in low-income regions, is critical to improving the accuracy of future analyses and enabling more reliable comparisons of the global burden of MNBAC across diverse populations.

## Conclusions

MNBAC in individuals aged ≥ 65 years constitutes a significant public health challenge, imposing considerable healthcare and economic burdens. As global populations continue to age, sustained focus on this condition remains imperative. This comprehensive analysis of the global, regional, and national burden of MNBAC among older adults provides critical insights into future disease trajectories. Such a nuanced understanding equips policymakers with the evidence necessary to devise strategic plans for targeted interventions and optimize healthcare resource allocation. By addressing the shifting demands posed by MNBAC and its associated comorbidities, these efforts will be instrumental in navigating the complexities of an evolving healthcare landscape.

## Supplementary Information

Below is the link to the electronic supplementary material.

Supplementary Figure S1 Total cases and rates of prevalence (A), incidence (B), mortality (C), and disability adjusted life years (DALYs) (D) of malignant neoplasms of bone and articular cartilage in people aged ≥65 years across gender and age groups from 1990 to 2021. Error bars indicate the 95% certainty interval for numbers. file1 (PDF 152 KB)

Supplementary Figure S2 Joinpoint regression analysis of malignant neoplasms of bone and articular cartilage prevalence (A), incidence (B), mortality (C), and disability adjusted life years (DALYs) (D) in people aged ≥65 years from 1990 to 2021. file2 (PDF 151 KB)

Supplementary Figure S3 Age-standardized rate of prevalence (A), incidence (B), mortality (C), and disability adjusted life years (DALYs) (D) due to malignant neoplasms of bone and articular cartilage in people aged ≥65 years by GBD regions and the expected value based on the SDI from1990 to 2021. file3 (PDF 94 KB)

Supplementary file4 (DOCX 21 KB)

Supplementary file5 (DOCX 47 KB)

Supplementary file6 (DOCX 46 KB)

Supplementary file7 (DOCX 46 KB)

Supplementary file8 (DOCX 48 KB)

## Data Availability

No datasets were generated or analysed during the current study. Data sources and code used in the Global Burden of Disease Study 2021 are available on the internet. (http://ghdx.healthdata.org/gbd-results-tool).
