# Supplementary figures and images for: Global, regional, and national burden of malignant neoplasm of bone and articular cartilage in adults aged 65 years and older, 1990–2021: a systematic analysis based on the global burden of disease study 2021
Source: Aging Clin Exp Res. 2025 Jan 8;37(1):21. doi: 10.1007/s40520-024-02926-0 (PMC11711276; doi:10.1007/s40520-024-02926-0)

**A**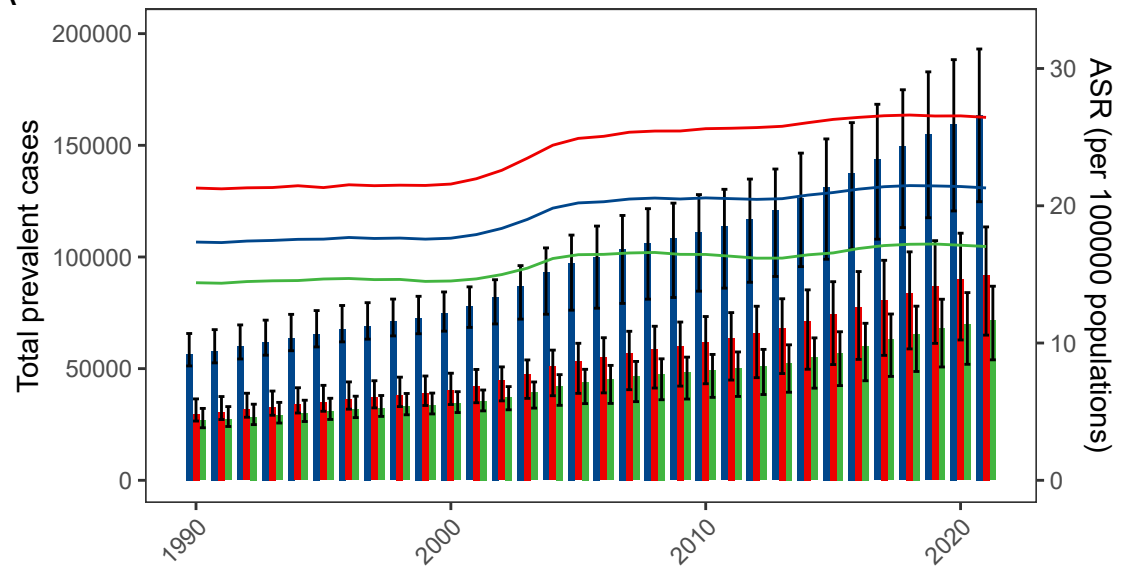**B**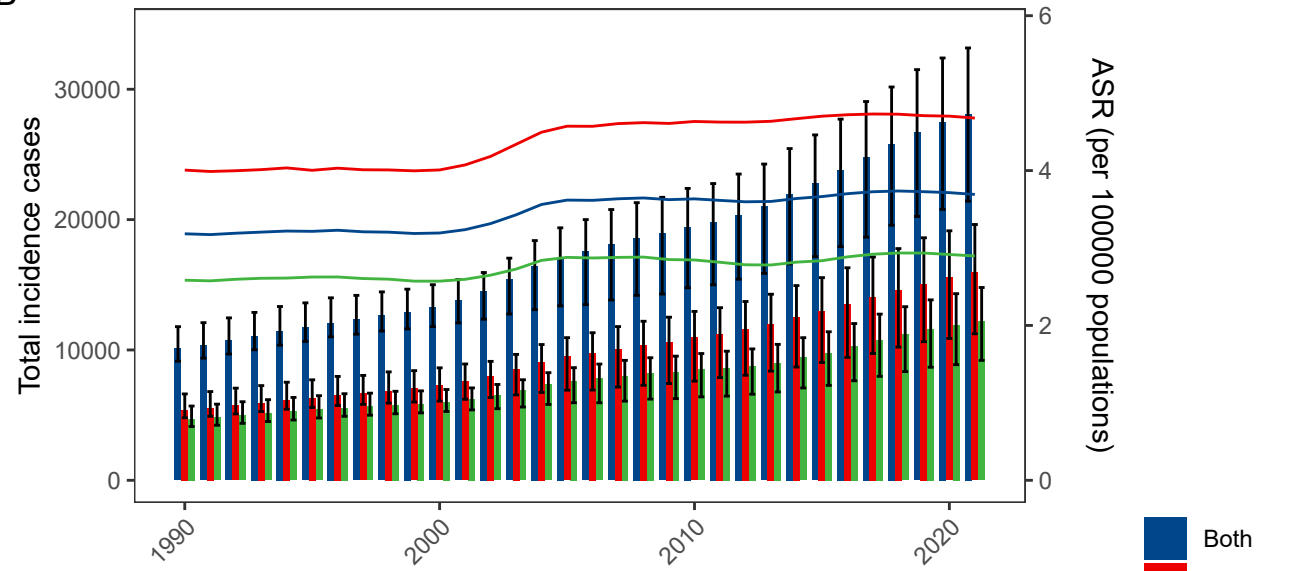**C**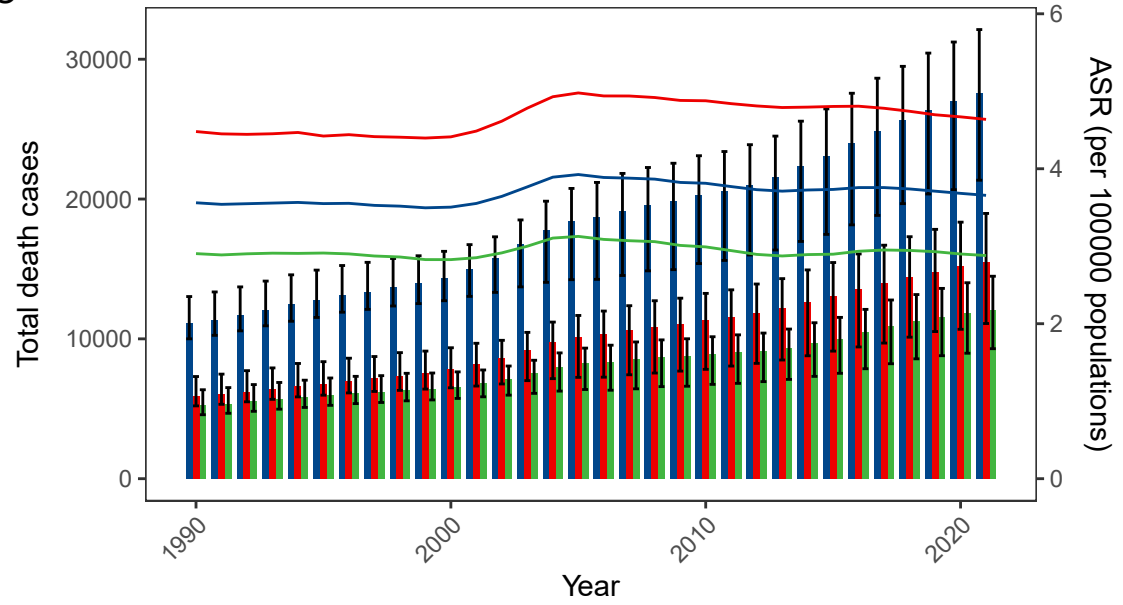**D**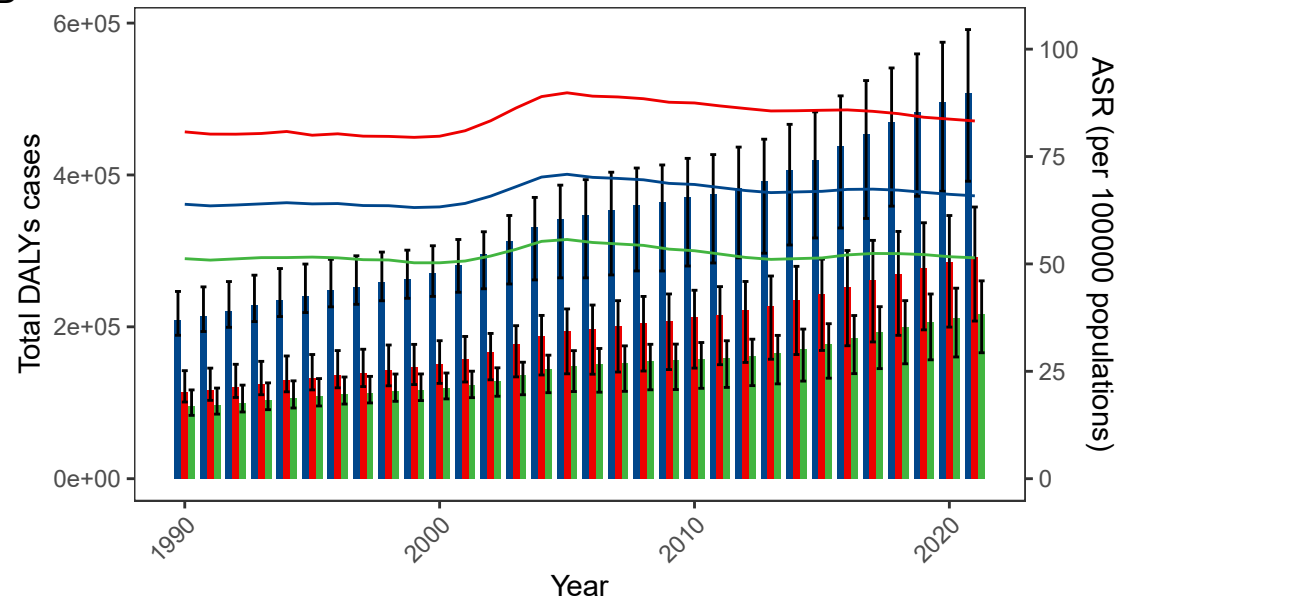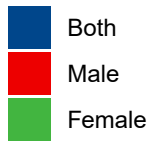

Supplement: Supplementary file 1 — Supplementary Figure S1 Total cases and rates of prevalence (A), incidence (B), mortality (C), and disability adjusted life years (DALYs) (D) of malignant neoplasms of bone and articular cartilage in people aged ≥65 years across gender and age groups from 1990 to 2021. Error bars indicate the 95% certainty interval for numbers. file1 (PDF 152 KB) [file 40520_2024_2926_MOESM1_ESM.pdf]

**A**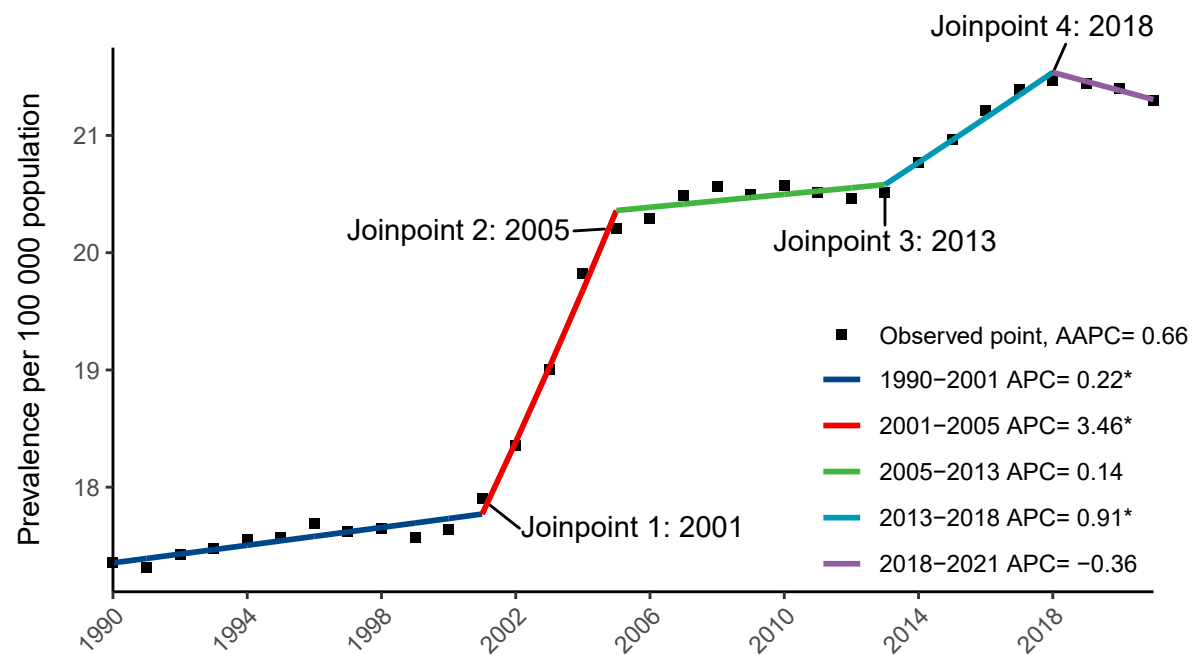**B**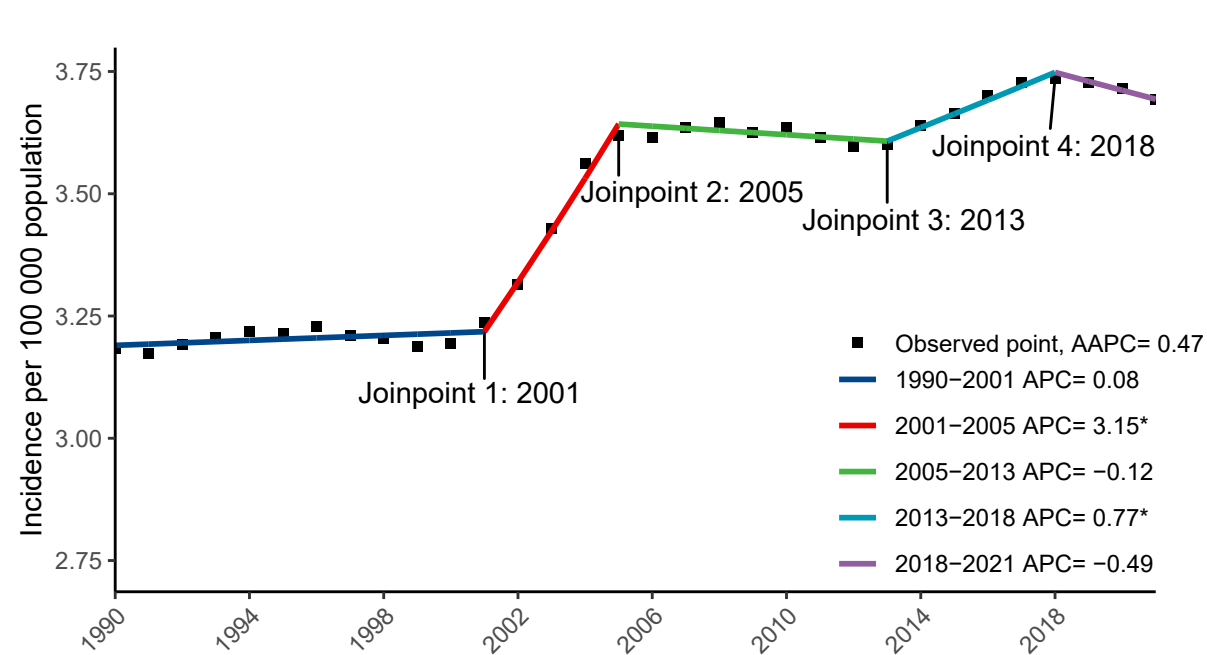**C**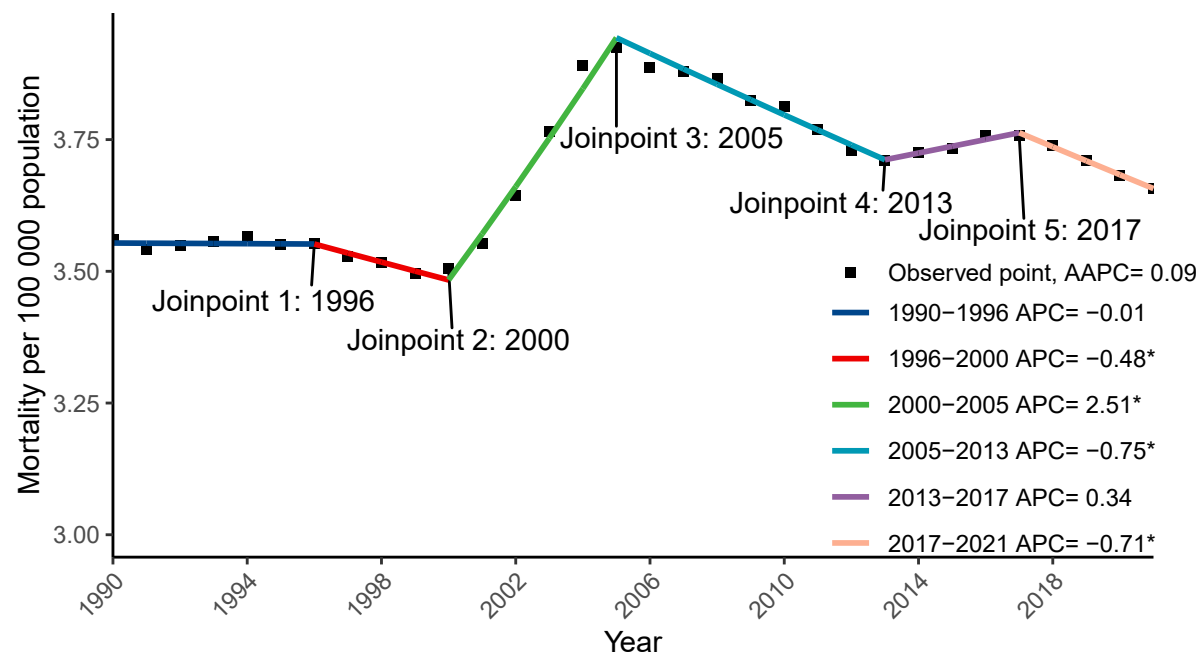**D**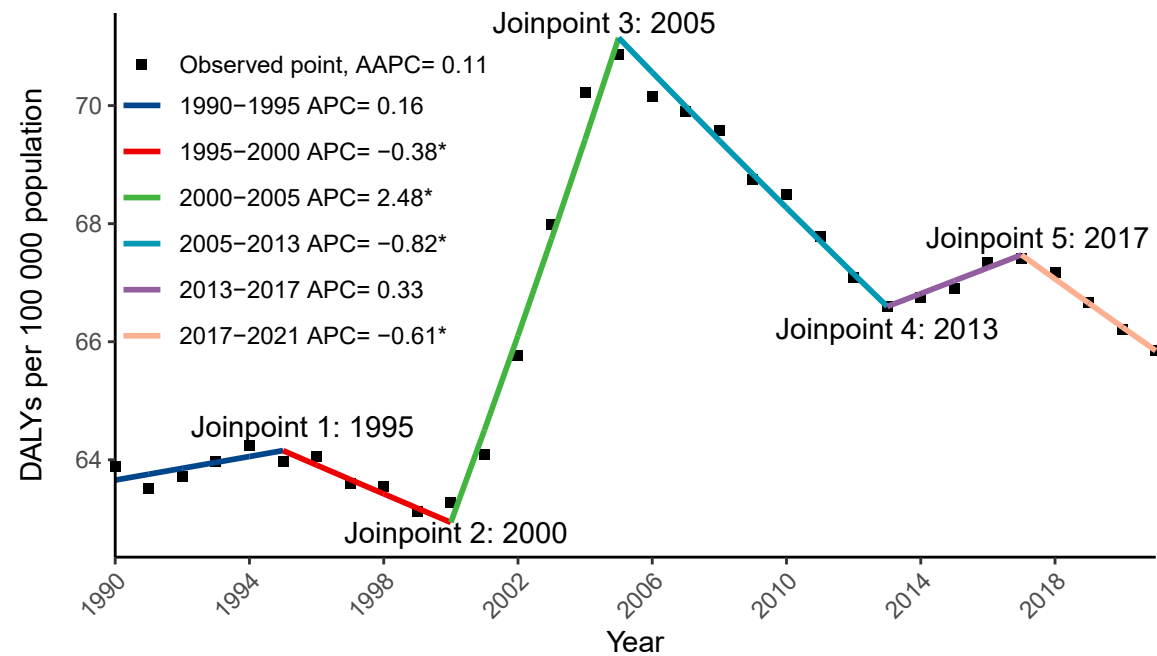

Supplement: Supplementary file 2 — Supplementary Figure S2 Joinpoint regression analysis of malignant neoplasms of bone and articular cartilage prevalence (A), incidence (B), mortality (C), and disability adjusted life years (DALYs) (D) in people aged ≥65 years from 1990 to 2021. file2 (PDF 151 KB) [file 40520_2024_2926_MOESM2_ESM.pdf]

A

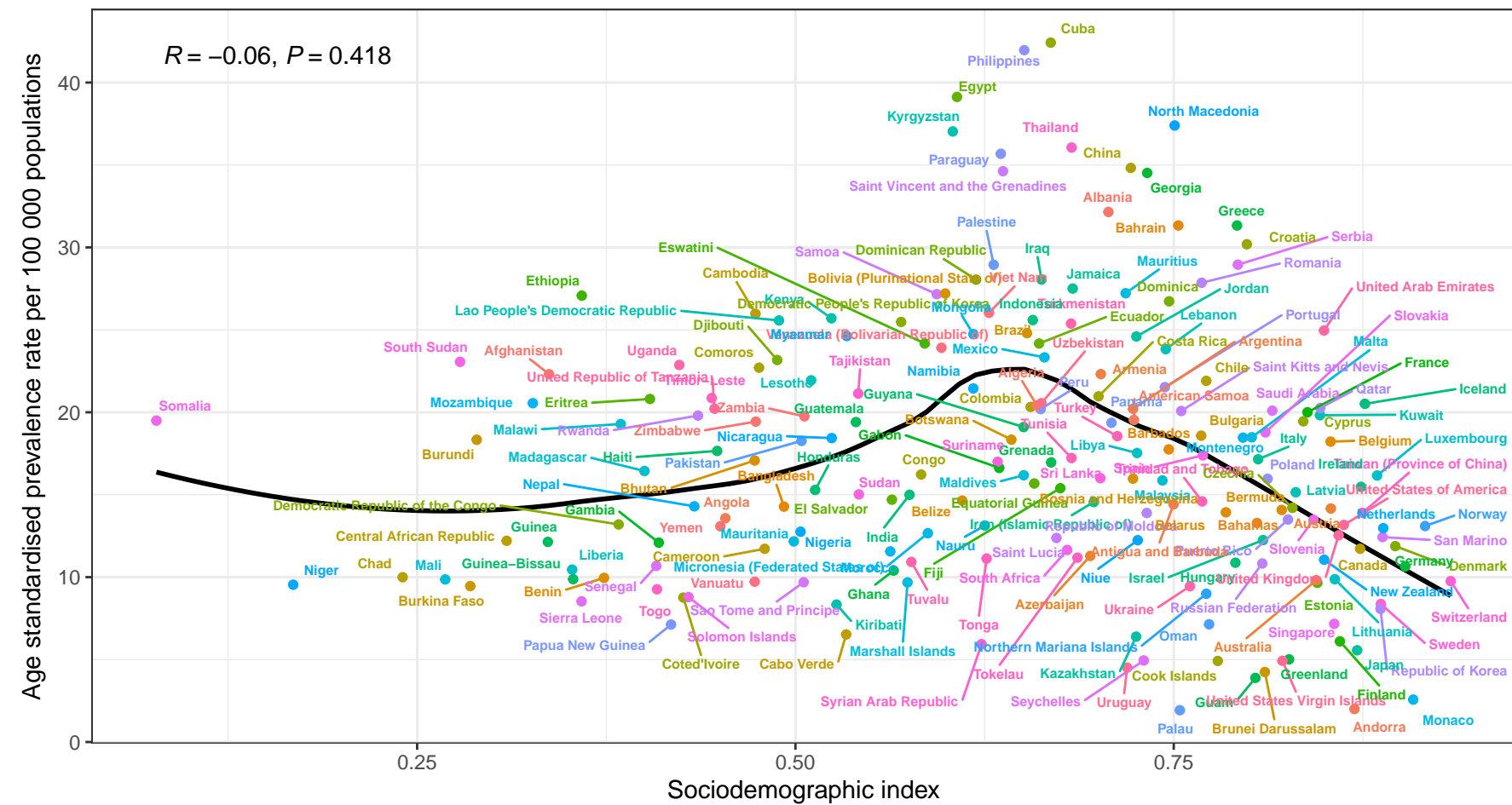

B

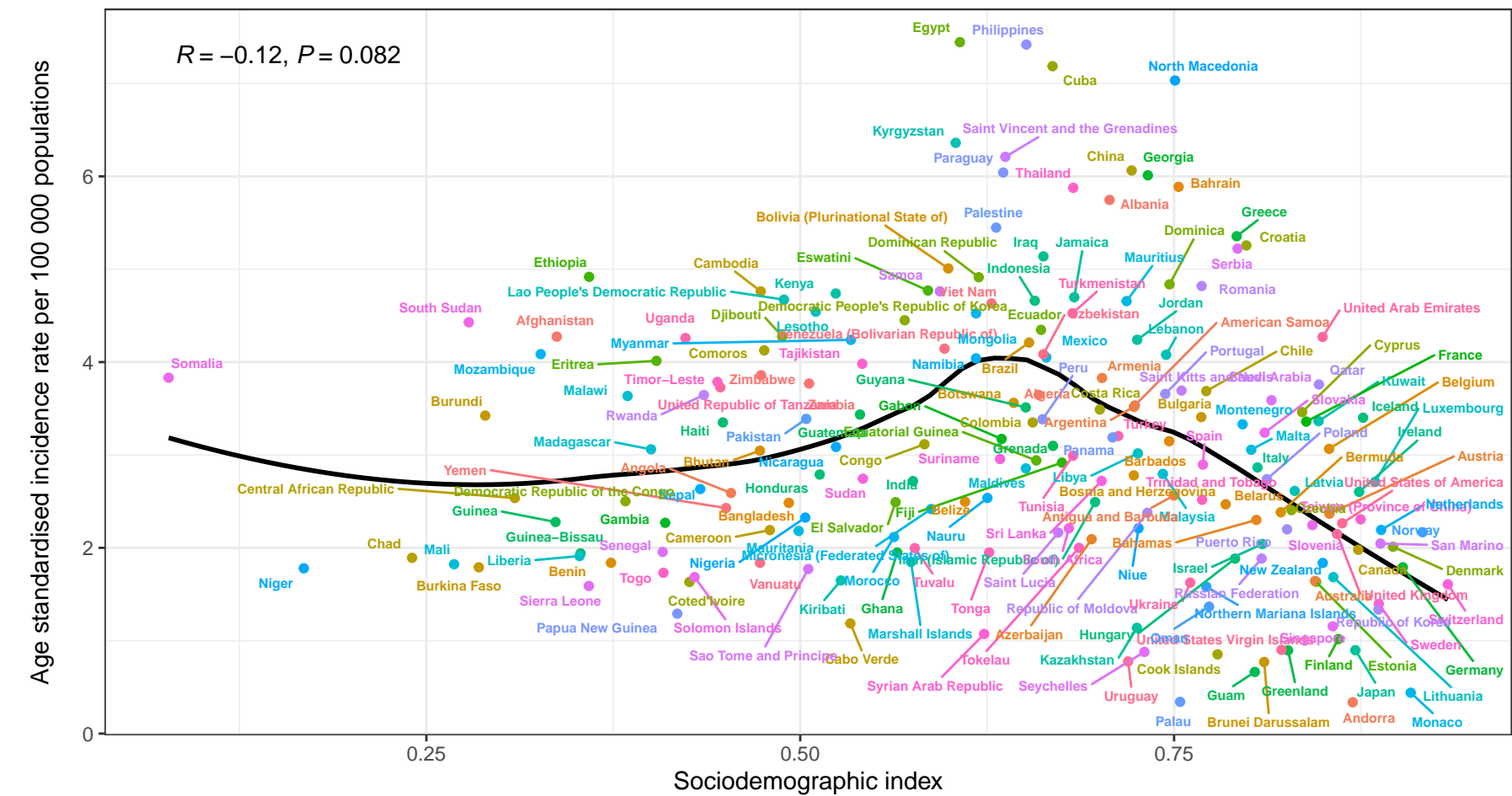

C

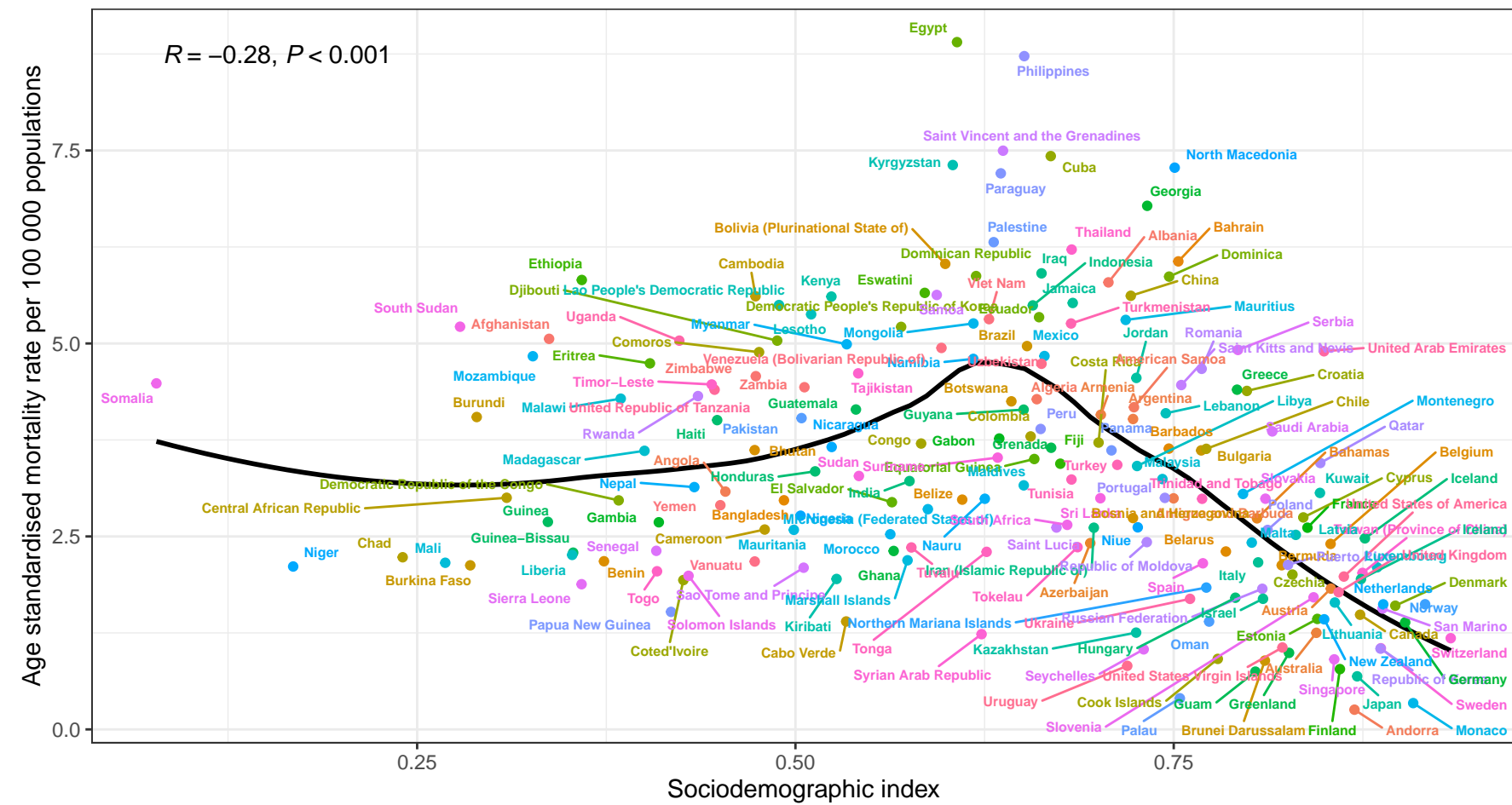

D

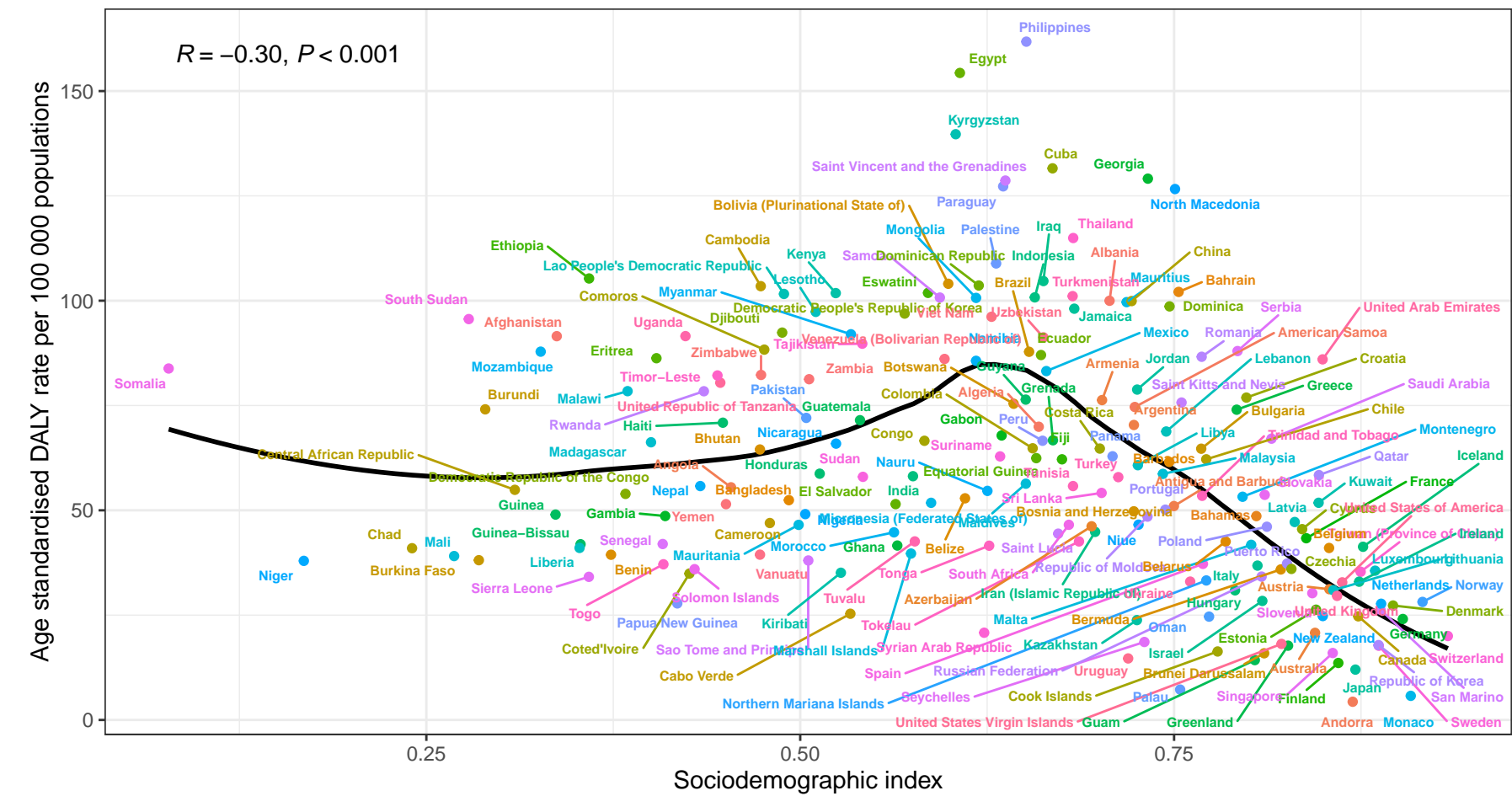

Supplement: Supplementary file 3 — Supplementary Figure S3 Age-standardized rate of prevalence (A), incidence (B), mortality (C), and disability adjusted life years (DALYs) (D) due to malignant neoplasms of bone and articular cartilage in people aged ≥65 years by GBD regions and the expected value based on the SDI from1990 to 2021. file3 (PDF 94 KB) [file 40520_2024_2926_MOESM3_ESM.pdf]
