# Supplementary material for: Global, regional, and national burden of malignant neoplasm of bone and articular cartilage in adults aged 65 years and older, 1990–2021: a systematic analysis based on the global burden of disease study 2021
Source: Aging Clin Exp Res. 2025 Jan 8;37(1):21. doi: 10.1007/s40520-024-02926-0 (PMC11711276; doi:10.1007/s40520-024-02926-0)
Supplement: Supplementary file 4 — Supplementary file4 (DOCX 21 KB) [file 40520_2024_2926_MOESM4_ESM.docx]

**Supplementary Materials**

**Global, regional, and national burden of malignant neoplasm of bone and articular cartilage** **in adults aged 65 years and older, 1990–2021: a systematic analysis for the global burden of disease study 2021**

**Supplementary Methods**

**1. Bayesian Age-Period-Cohort (BAPC) Modeling:**

The Bayesian age-period-cohort (BAPC) model was utilized to forecast MNBAC incidence, prevalence, and DALYs rates from 2022 to 2050. This model, fundamentally a logarithmic linear Poisson construct, postulates the multiplicative effects of age, period, and cohort, all presumed to adhere to a Poisson distribution and employ a model-specific link function.^[1]^ Projections were extrapolated from the 2021 database, with the age-standardized rates of MNBAC incidence, prevalence, and DALYs in 2021 serving as the baseline. A pessimistic scenario was simulated with an annual increment of 1%, while an optimistic scenario envisaged an annual decrement of 1%. The BAPC model was executed using the R package BAPC.

**2. Estimated annual percent change (EAPC)**

In this study, we used the estimated annual percent change (EAPC) to analyze trends in the age-standardized rates (ASR) of prevalence, incidence, mortality, and DALYs for MNBAC in adults aged 65 years and older across various global regions from 1990 to 2021. The EAPC is a commonly used indicator to reflect ASR trends over a specified period.^[2]^ We fitted a regression line to the natural logarithm of the ASR, modeled as (y = a + bx + e), where x represents the calendar year. The EAPC was calculated using the formula (100 × [exp(b) – 1]). An EAPC >0 indicates an increasing ASR trend, whereas an EAPC <0 indicates a decreasing trend; otherwise, the ASR is considered stable.

**3. Joinpoint regression analysis**

We conducted a Joinpoint regression analysis to examine trends in prevalence, incidence, mortality, and DALYs rates for MNBAC among adults aged 65 years and older across global regions from 1990 to 2021. The Joinpoint Regression Program (version 4.9.0.0) developed by the National Cancer Institute was utilized to identify significant changes in trends over time.^[3]^ The analysis allowed for up to five joinpoints, with a minimum of four years between joinpoints. We calculated the annual percentage change (APC) for each identified trend segment and the average annual percentage change (AAPC) for the entire study period. The final models were selected based on the Monte Carlo Permutation method, with a significance level set at 0.05 for the permutation test. This approach enabled us to identify both the magnitude and the timing of significant changes in trends for each indicator across different global regions.

References:

[1]. Jürgens, V., et al., *A Bayesian generalized age-period-cohort power model for cancer projections.* Stat Med, 2014. **33**(26): p. 4627-36.

[2]. Hankey, B.F., et al., *Partitioning linear trends in age-adjusted rates.* Cancer Causes Control, 2000. **11**(1): p. 31-5.

[3]. Kim, H.J., et al., *Permutation tests for joinpoint regression with applications to cancer rates.* Stat Med, 2000. **19**(3): p. 335-51.
