# Supplementary material for: Global, regional, and national burden of malignant neoplasm of bone and articular cartilage in adults aged 65 years and older, 1990–2021: a systematic analysis based on the global burden of disease study 2021
Source: Aging Clin Exp Res. 2025 Jan 8;37(1):21. doi: 10.1007/s40520-024-02926-0 (PMC11711276; doi:10.1007/s40520-024-02926-0)
Supplement: Supplementary file 5 — Supplementary file5 (DOCX 47 KB) [file 40520_2024_2926_MOESM5_ESM.docx]

Table S2 MNBAC prevalence in people aged ≥65 years in 1990 and 2021 for both sexes and EAPC in age-standardized rates by location

| location | Number in 1990 (95% CI) | Rate in 1990 (95% CI) | Number in 2021 (95% CI) | Rate in 2021 (95% CI) | EAPC in age-standardized rates between 1990 and 2021 (95%CI) |
| --- | --- | --- | --- | --- | --- |
| Global | 56527.41 (51214.25, 65725.67) | 17.35 (15.67, 20.13) | 163561.30 (124738.37, 193129.06) | 21.30 (16.26, 25.14) | 0.85 (0.75, 0.95) |
| High SDI | 13837.11 (12658.33, 14854.64) | 13.23 (12.08, 14.22) | 26289.16 (22704.39, 29474.53) | 12.69 (11.02, 14.19) | -0.22 (-0.31, -0.12) |
| High-middle SDI | 20421.66 (18464.43, 23128.85) | 24.49 (22.07, 27.71) | 49849.16 (33232.98, 65299.54) | 27.21 (18.17, 35.61) | 0.61 (0.46, 0.76) |
| Middle SDI | 12804.55 (10341.87, 17980.50) | 16.69 (13.52, 23.23) | 60590.99 (44256.70, 74201.24) | 26.42 (19.35, 32.28) | 1.90 (1.66, 2.15) |
| Low-middle SDI | 6723.30 (5208.60, 8065.14) | 15.13 (11.75, 18.16) | 20743.93 (16679.39, 24744.19) | 18.18 (14.64, 21.68) | 0.59 (0.56, 0.61) |
| Low SDI | 2650.25 (2033.24, 3308.98) | 15.97 (12.22, 19.99) | 5933.65 (4647.56, 7852.04) | 16.03 (12.55, 21.19) | -0.04 (-0.12, 0.03) |
| Eastern Sub-Saharan Africa | 1322.81 (1031.63, 1743.27) | 23.95 (18.62, 31.59) | 2706.83 (1964.89, 4006.45) | 22.88 (16.63, 33.76) | -0.24 (-0.29, -0.20) |
| Western Sub-Saharan Africa | 779.48 (546.60, 1027.63) | 11.56 (8.13, 15.21) | 1540.35 (1158.86, 2019.96) | 11.38 (8.58, 14.92) | -0.09 (-0.12, -0.05) |
| Central Sub-Saharan Africa | 235.80 (146.86, 370.38) | 15.71 (9.67, 24.81) | 466.89 (263.01, 717.96) | 13.49 (7.58, 20.82) | -0.57 (-0.70, -0.44) |
| North Africa and Middle East | 2163.24 (1632.51, 2874.44) | 17.97 (13.53, 23.91) | 6657.95 (5196.10, 8916.83) | 19.96 (15.55, 26.78) | 0.41 (0.32, 0.50) |
| Oceania | 14.77 (8.35, 29.14) | 7.76 (4.45, 15.06) | 42.72 (20.47, 86.47) | 8.91 (4.30, 17.97) | 0.52 (0.33, 0.70) |
| South Asia | 5231.28 (3872.57, 6476.11) | 12.81 (9.47, 15.90) | 18112.57 (14546.01, 23383.84) | 15.15 (12.15, 19.54) | 0.43 (0.35, 0.51) |
| Southeast Asia | 3379.04 (2689.01, 4230.69) | 18.12 (14.39, 22.72) | 14663.50 (9581.85, 18999.29) | 28.67 (18.89, 37.05) | 1.74 (1.59, 1.88) |
| Caribbean | 486.13 (410.04, 574.73) | 21.56 (18.17, 25.50) | 1368.89 (1113.93, 1645.37) | 28.90 (23.52, 34.73) | 0.78 (0.58, 0.98) |
| Southern Sub-Saharan Africa | 301.55 (204.87, 390.09) | 14.48 (9.78, 18.78) | 584.63 (477.14, 735.54) | 13.15 (10.70, 16.46) | -0.58 (-0.78, -0.38) |
| Central Latin America | 1246.58 (1162.13, 1321.24) | 19.64 (18.26, 20.84) | 4587.34 (4036.10, 5138.91) | 21.92 (19.27, 24.55) | 0.55 (0.30, 0.81) |
| Central Asia | 623.78 (478.64, 762.62) | 17.71 (13.57, 21.66) | 1154.50 (967.02, 1356.81) | 18.93 (15.87, 22.22) | 0.30 (0.08, 0.51) |
| Andean Latin America | 380.28 (297.81, 489.67) | 24.10 (18.87, 31.01) | 1113.75 (811.07, 1521.97) | 22.36 (16.28, 30.55) | -0.39 (-0.50, -0.29) |
| Tropical Latin America | 2036.36 (1860.42, 2188.19) | 28.79 (26.15, 31.02) | 5520.86 (4930.87, 5997.87) | 25.03 (22.29, 27.22) | -0.22 (-0.38, -0.06) |
| Central Europe | 3554.73 (3134.18, 3976.88) | 26.87 (23.67, 30.04) | 4421.68 (3839.53, 5039.27) | 19.78 (17.18, 22.55) | -1.13 (-1.32, -0.94) |
| Southern Latin America | 1330.15 (1090.11, 1610.15) | 32.37 (26.49, 39.20) | 1599.84 (1348.03, 1878.30) | 19.65 (16.57, 23.07) | -1.62 (-1.85, -1.39) |
| East Asia | 9776.56 (6315.64, 17011.99) | 14.46 (9.35, 25.16) | 69378.73 (41038.11, 95117.20) | 34.28 (20.31, 46.93) | 3.73 (3.13, 4.33) |
| Eastern Europe | 7040.88 (6597.14, 7440.93) | 29.57 (27.66, 31.28) | 3611.58 (3196.31, 4032.67) | 10.73 (9.50, 11.99) | -4.05 (-4.35, -3.74) |
| Western Europe | 11045.15 (10016.55, 11988.00) | 19.61 (17.78, 21.29) | 14279.85 (12161.01, 16173.36) | 15.21 (13.09, 17.15) | -0.87 (-0.99, -0.75) |
| High-income Asia Pacific | 1341.14 (1200.80, 1483.31) | 7.72 (6.87, 8.55) | 2869.35 (2344.33, 3359.15) | 6.04 (5.00, 7.06) | -0.66 (-0.88, -0.43) |
| High-income North America | 3912.20 (3539.66, 4224.51) | 11.34 (10.25, 12.26) | 8348.69 (7337.97, 9149.76) | 13.01 (11.44, 14.24) | 0.28 (0.10, 0.47) |
| Australasia | 325.51 (276.14, 378.86) | 14.58 (12.33, 17.00) | 530.80 (420.29, 657.55) | 10.01 (7.95, 12.39) | -1.25 (-1.44, -1.05) |
| Mexico | 656.12 (621.64, 687.21) | 20.19 (19.09, 21.17) | 2434.26 (2134.56, 2736.22) | 23.33 (20.47, 26.21) | 1.09 (0.58, 1.59) |
| Guatemala | 50.55 (41.71, 59.36) | 21.37 (17.40, 25.19) | 177.57 (147.07, 213.95) | 19.41 (16.07, 23.36) | -0.20 (-0.44, 0.04) |
| Guinea | 32.77 (18.56, 50.40) | 11.71 (6.65, 18.01) | 51.74 (26.66, 85.47) | 12.14 (6.28, 19.98) | 0.03 (-0.04, 0.09) |
| Gambia | 2.78 (1.57, 4.53) | 10.65 (6.02, 17.27) | 8.78 (4.54, 14.15) | 12.09 (6.26, 19.52) | 0.20 (0.08, 0.32) |
| El Salvador | 27.13 (19.58, 36.00) | 11.17 (8.06, 14.83) | 81.92 (51.15, 123.14) | 14.70 (9.18, 22.07) | 0.90 (0.84, 0.95) |
| Costa Rica | 20.58 (16.44, 25.56) | 14.32 (11.44, 17.78) | 99.75 (75.31, 127.94) | 20.97 (15.84, 26.89) | 1.60 (1.36, 1.84) |
| Cabo Verde | 1.31 (0.74, 2.37) | 6.04 (3.40, 10.93) | 2.07 (1.12, 3.71) | 6.53 (3.54, 11.82) | 0.22 (0.14, 0.30) |
| Peru | 173.17 (109.69, 270.50) | 18.66 (11.81, 29.11) | 573.52 (347.39, 901.28) | 20.19 (12.23, 31.73) | -0.05 (-0.23, 0.13) |
| Paraguay | 56.76 (33.95, 83.58) | 31.66 (18.94, 46.65) | 168.58 (98.95, 258.77) | 35.68 (20.97, 54.73) | 0.43 (0.23, 0.63) |
| Guinea-Bissau | 3.18 (1.73, 5.71) | 10.75 (5.88, 19.21) | 4.58 (2.49, 7.71) | 9.88 (5.36, 16.57) | -0.33 (-0.36, -0.29) |
| Colombia | 284.06 (239.37, 334.65) | 21.46 (18.04, 25.32) | 972.91 (746.65, 1233.93) | 20.32 (15.62, 25.76) | -0.76 (-0.97, -0.55) |
| Sao Tome and Principe | 0.48 (0.28, 0.88) | 8.98 (5.12, 16.36) | 0.74 (0.42, 1.20) | 9.69 (5.42, 15.67) | 0.13 (0.07, 0.20) |
| Saint Vincent and the Grenadines | 0.67 (0.58, 0.76) | 9.97 (8.63, 11.38) | 4.33 (3.64, 5.10) | 34.62 (29.17, 40.84) | 3.48 (2.20, 4.78) |
| Trinidad and Tobago | 14.12 (12.33, 16.23) | 18.95 (16.51, 21.81) | 25.67 (19.32, 32.87) | 14.59 (11.01, 18.64) | -0.82 (-0.99, -0.64) |
| Jamaica | 48.06 (35.92, 61.91) | 28.19 (21.07, 36.34) | 72.58 (51.39, 99.74) | 27.51 (19.47, 37.82) | 0.04 (-0.22, 0.30) |
| Turkey | 441.04 (273.30, 696.39) | 17.96 (11.14, 28.33) | 1476.45 (864.68, 2215.83) | 18.55 (10.89, 27.82) | 0.16 (-0.13, 0.45) |
| Bermuda | 0.77 (0.58, 1.01) | 14.41 (10.74, 18.83) | 1.88 (1.38, 2.57) | 14.08 (10.34, 19.24) | 0.01 (-0.18, 0.20) |
| Saudi Arabia | 64.28 (36.37, 102.40) | 16.35 (9.27, 26.02) | 195.08 (111.20, 308.49) | 20.10 (11.59, 31.79) | 0.61 (0.54, 0.69) |
| Burundi | 34.65 (18.09, 54.32) | 18.71 (9.78, 29.28) | 59.42 (26.62, 101.47) | 18.33 (8.28, 31.27) | -0.03 (-0.09, 0.03) |
| Morocco | 114.34 (65.28, 185.98) | 10.06 (5.74, 16.34) | 313.76 (181.08, 515.56) | 11.56 (6.68, 18.94) | 0.56 (0.47, 0.66) |
| Palestine | 16.14 (8.57, 25.11) | 23.65 (12.58, 36.80) | 50.16 (31.94, 77.57) | 28.95 (18.46, 44.75) | 0.89 (0.70, 1.07) |
| Bahrain | 2.58 (1.63, 4.31) | 25.03 (15.77, 41.79) | 15.41 (9.25, 25.98) | 31.33 (18.85, 53.12) | 0.75 (0.56, 0.93) |
| Algeria | 179.50 (107.83, 279.77) | 18.79 (11.37, 29.54) | 556.79 (284.53, 897.04) | 20.43 (10.48, 32.88) | 0.32 (0.20, 0.44) |
| Central African Republic | 10.50 (5.58, 18.51) | 14.45 (7.67, 25.44) | 15.23 (7.21, 24.56) | 12.21 (5.70, 19.81) | -0.61 (-0.65, -0.57) |
| Syrian Arab Republic | 18.14 (9.44, 35.13) | 4.99 (2.59, 9.65) | 57.68 (29.82, 108.45) | 5.94 (3.07, 11.24) | 0.30 (0.17, 0.44) |
| Equatorial Guinea | 1.91 (0.98, 3.45) | 14.26 (7.24, 25.79) | 5.21 (2.53, 8.80) | 15.68 (7.68, 26.33) | 0.52 (0.41, 0.63) |
| Iran (Islamic Republic of) | 221.84 (173.59, 285.00) | 12.58 (9.82, 16.10) | 854.38 (653.44, 1138.26) | 14.56 (11.12, 19.40) | 0.56 (0.50, 0.63) |
| Jordan | 16.53 (9.59, 25.33) | 19.39 (11.19, 29.73) | 122.44 (72.24, 197.95) | 24.60 (14.51, 39.80) | 1.03 (0.89, 1.18) |
| Nepal | 79.21 (46.35, 128.28) | 12.11 (7.08, 19.62) | 271.55 (154.39, 433.22) | 14.30 (8.13, 22.79) | 0.55 (0.44, 0.67) |
| Congo | 13.16 (7.35, 21.03) | 16.90 (9.45, 27.16) | 27.79 (14.30, 44.66) | 16.22 (8.38, 26.01) | -0.23 (-0.28, -0.17) |
| Democratic Republic of the Congo | 168.52 (91.46, 286.85) | 16.09 (8.63, 27.72) | 307.15 (145.32, 506.43) | 13.20 (6.23, 21.88) | -0.72 (-0.88, -0.56) |
| Libya | 19.71 (10.87, 32.43) | 14.32 (7.89, 23.60) | 60.88 (33.87, 100.44) | 17.53 (9.75, 28.87) | 1.00 (0.86, 1.14) |
| Comoros | 3.20 (1.60, 5.07) | 21.88 (10.99, 34.62) | 8.42 (3.74, 15.28) | 22.70 (10.10, 41.12) | 0.03 (-0.03, 0.09) |
| Lebanon | 29.54 (17.09, 46.12) | 18.26 (10.56, 28.43) | 133.83 (73.86, 205.38) | 23.83 (13.11, 36.54) | 1.06 (0.83, 1.28) |
| Kuwait | 4.98 (3.79, 6.33) | 14.76 (11.22, 18.77) | 32.72 (23.18, 44.80) | 19.82 (14.04, 27.16) | 1.72 (0.33, 3.12) |
| Gabon | 7.80 (4.13, 13.06) | 16.78 (8.88, 28.19) | 12.28 (6.22, 20.26) | 16.62 (8.46, 27.43) | -0.19 (-0.29, -0.10) |
| Sudan | 98.38 (53.56, 167.89) | 13.05 (7.07, 22.34) | 206.86 (122.25, 348.94) | 15.02 (8.88, 25.35) | 0.50 (0.43, 0.57) |
| Djibouti | 1.59 (0.78, 2.67) | 19.76 (9.75, 32.98) | 9.24 (4.15, 16.71) | 23.17 (10.41, 41.37) | 0.53 (0.47, 0.60) |
| Eritrea | 10.49 (5.09, 16.19) | 17.63 (8.62, 27.43) | 35.22 (15.40, 61.41) | 20.80 (9.13, 36.34) | 0.34 (0.24, 0.44) |
| Kenya | 143.61 (106.90, 187.32) | 23.19 (17.23, 30.22) | 422.47 (309.79, 598.83) | 25.70 (18.86, 36.27) | 0.45 (0.38, 0.52) |
| Iraq | 157.25 (84.24, 242.20) | 25.49 (13.65, 39.25) | 466.80 (276.35, 716.70) | 28.05 (16.67, 42.88) | 0.43 (0.32, 0.54) |
| Malawi | 56.27 (28.09, 85.67) | 19.62 (9.82, 29.93) | 104.26 (49.76, 177.94) | 19.29 (9.20, 32.97) | -0.00 (-0.04, 0.04) |
| Angola | 33.91 (17.27, 61.37) | 14.02 (7.13, 25.59) | 99.23 (47.38, 162.18) | 13.57 (6.49, 22.30) | -0.15 (-0.24, -0.06) |
| Seychelles | 0.26 (0.07, 0.56) | 4.95 (1.39, 10.92) | 0.44 (0.11, 0.97) | 4.95 (1.20, 10.80) | 0.18 (-0.23, 0.59) |
| Madagascar | 75.01 (39.08, 117.62) | 19.79 (10.35, 30.91) | 112.76 (50.44, 192.15) | 16.44 (7.38, 27.87) | -0.49 (-0.71, -0.28) |
| Ethiopia | 434.64 (305.24, 610.46) | 30.39 (21.24, 42.88) | 875.97 (595.20, 1213.46) | 27.08 (18.43, 37.48) | -0.68 (-0.78, -0.57) |
| Mozambique | 91.37 (45.13, 141.46) | 21.39 (10.58, 33.15) | 157.83 (72.67, 262.08) | 20.55 (9.42, 34.40) | -0.08 (-0.16, 0.01) |
| Rwanda | 44.92 (22.91, 68.74) | 21.70 (11.05, 33.13) | 88.52 (39.39, 160.62) | 19.80 (8.88, 35.88) | -0.22 (-0.50, 0.06) |
| Uganda | 114.09 (68.25, 170.57) | 22.95 (13.73, 34.30) | 232.36 (122.54, 375.24) | 22.87 (12.09, 36.98) | -0.25 (-0.36, -0.14) |
| Mauritius | 16.42 (14.44, 18.50) | 27.71 (24.37, 31.19) | 44.97 (38.88, 51.47) | 27.22 (23.52, 31.12) | 1.31 (-0.20, 2.85) |
| Somalia | 27.33 (13.32, 43.55) | 20.86 (10.12, 33.15) | 76.25 (31.56, 128.93) | 19.50 (8.08, 33.33) | -0.15 (-0.18, -0.12) |
| United Republic of Tanzania | 191.20 (98.59, 294.79) | 22.65 (11.73, 34.88) | 374.97 (171.13, 657.95) | 20.22 (9.28, 35.42) | -0.40 (-0.48, -0.33) |
| Eswatini | 4.50 (2.60, 7.27) | 22.55 (12.98, 36.29) | 9.55 (4.78, 16.28) | 24.18 (12.09, 41.40) | 0.18 (0.01, 0.36) |
| Zambia | 43.80 (22.13, 66.93) | 21.90 (11.13, 33.50) | 90.89 (39.99, 161.74) | 19.75 (8.81, 35.03) | -0.39 (-0.45, -0.32) |
| Chad | 22.15 (11.76, 41.17) | 9.01 (4.77, 16.75) | 41.12 (19.41, 74.58) | 9.99 (4.74, 18.03) | 0.32 (0.29, 0.36) |
| Benin | 17.38 (10.17, 30.51) | 10.57 (6.19, 18.54) | 36.81 (20.07, 61.43) | 9.95 (5.43, 16.59) | -0.30 (-0.37, -0.23) |
| Coted'Ivoire | 22.21 (13.26, 38.76) | 9.17 (5.51, 15.88) | 62.97 (36.28, 107.54) | 8.76 (5.04, 14.87) | -0.13 (-0.17, -0.10) |
| Ghana | 39.38 (21.43, 74.50) | 8.92 (4.87, 16.81) | 123.50 (66.21, 205.85) | 10.40 (5.58, 17.35) | 0.51 (0.45, 0.56) |
| Cameroon | 42.27 (25.77, 71.87) | 13.56 (8.26, 22.96) | 100.52 (53.63, 172.25) | 11.72 (6.27, 19.99) | -0.55 (-0.63, -0.46) |
| Liberia | 11.82 (6.96, 22.13) | 11.85 (6.95, 22.15) | 14.24 (7.82, 24.18) | 10.46 (5.74, 17.71) | -0.32 (-0.48, -0.16) |
| Niger | 19.85 (10.74, 36.16) | 10.45 (5.63, 18.94) | 55.18 (27.82, 95.17) | 9.54 (4.80, 16.38) | -0.39 (-0.45, -0.34) |
| Mali | 28.83 (17.32, 48.18) | 9.94 (6.00, 16.56) | 62.36 (35.02, 103.86) | 9.86 (5.56, 16.41) | -0.03 (-0.08, 0.01) |
| Sierra Leone | 15.40 (8.18, 28.72) | 8.87 (4.71, 16.52) | 23.86 (12.52, 42.05) | 8.53 (4.50, 14.94) | -0.22 (-0.32, -0.12) |
| Senegal | 27.45 (15.54, 48.26) | 10.81 (6.11, 18.98) | 62.29 (32.30, 105.54) | 10.68 (5.56, 18.06) | -0.01 (-0.10, 0.07) |
| Cook Islands | 0.05 (0.03, 0.09) | 4.85 (2.73, 8.84) | 0.12 (0.06, 0.23) | 4.92 (2.61, 9.62) | -0.13 (-0.25, -0.01) |
| Greenland | 0.19 (0.09, 0.33) | 8.83 (4.21, 15.39) | 0.26 (0.15, 0.43) | 5.01 (2.82, 8.09) | -1.56 (-1.98, -1.13) |
| Guam | 0.32 (0.19, 0.51) | 6.36 (3.84, 10.20) | 0.73 (0.45, 1.08) | 3.89 (2.43, 5.78) | -1.13 (-1.36, -0.90) |
| Niue | 0.02 (0.01, 0.05) | 9.83 (4.10, 22.67) | 0.02 (0.01, 0.05) | 12.24 (4.15, 27.77) | 0.72 (0.61, 0.82) |
| Palau | 0.01 (0.01, 0.03) | 1.76 (0.69, 4.10) | 0.03 (0.01, 0.07) | 1.93 (0.76, 4.46) | 0.53 (0.37, 0.69) |
| Nigeria | 437.20 (297.22, 613.59) | 12.63 (8.61, 17.61) | 780.25 (563.67, 1097.82) | 12.76 (9.24, 17.87) | 0.03 (-0.06, 0.11) |
| Northern Mariana Islands | 0.07 (0.04, 0.12) | 8.47 (4.54, 14.18) | 0.33 (0.20, 0.54) | 8.99 (5.50, 14.74) | 0.62 (0.16, 1.09) |
| Mauritania | 10.27 (6.04, 17.73) | 11.95 (7.05, 20.60) | 20.39 (10.56, 35.27) | 12.17 (6.32, 20.99) | -0.13 (-0.18, -0.07) |
| Togo | 8.53 (4.89, 15.36) | 9.79 (5.62, 17.61) | 23.36 (12.61, 40.64) | 9.26 (5.03, 16.04) | -0.36 (-0.42, -0.30) |
| Saint Kitts and Nevis | 1.01 (0.87, 1.17) | 25.28 (21.75, 29.14) | 1.00 (0.81, 1.20) | 20.08 (16.33, 24.02) | -0.22 (-0.59, 0.15) |
| American Samoa | 0.25 (0.13, 0.40) | 16.02 (8.54, 25.76) | 0.71 (0.43, 1.17) | 19.54 (11.84, 32.14) | 1.03 (0.77, 1.28) |
| Puerto Rico | 22.60 (17.54, 28.64) | 6.66 (5.17, 8.44) | 98.42 (72.90, 128.14) | 13.49 (10.00, 17.57) | 2.63 (1.28, 4.00) |
| Monaco | 0.18 (0.06, 0.38) | 2.44 (0.81, 5.22) | 0.26 (0.10, 0.52) | 2.57 (0.98, 5.18) | -0.08 (-0.21, 0.04) |
| Nauru | 0.03 (0.01, 0.06) | 10.06 (4.22, 20.69) | 0.05 (0.02, 0.12) | 13.13 (4.32, 29.03) | 0.79 (0.71, 0.86) |
| Tokelau | 0.01 (0.00, 0.03) | 8.96 (3.31, 21.72) | 0.02 (0.00, 0.04) | 11.18 (3.48, 26.04) | 0.76 (0.66, 0.87) |
| San Marino | 0.79 (0.43, 1.25) | 23.15 (12.59, 36.38) | 0.92 (0.44, 1.62) | 12.43 (5.92, 21.82) | -1.33 (-1.67, -0.99) |
| South Sudan | 49.70 (23.41, 80.34) | 22.73 (10.77, 36.74) | 55.88 (23.18, 103.84) | 23.06 (9.58, 42.59) | -0.00 (-0.03, 0.02) |
| Tuvalu | 0.04 (0.02, 0.10) | 8.22 (2.94, 20.56) | 0.09 (0.03, 0.23) | 10.92 (3.28, 26.84) | 0.93 (0.79, 1.07) |
| United States Virgin Islands | 0.36 (0.23, 0.60) | 5.71 (3.54, 9.42) | 0.93 (0.47, 1.74) | 4.93 (2.50, 9.26) | -0.06 (-0.25, 0.12) |
| Taiwan (Province of China) | 320.69 (269.62, 374.66) | 24.45 (20.48, 28.63) | 544.76 (417.35, 691.61) | 13.90 (10.66, 17.65) | -1.42 (-1.89, -0.96) |
| Uzbekistan | 179.76 (90.43, 271.47) | 20.79 (10.56, 31.30) | 385.71 (280.82, 511.35) | 20.55 (14.89, 27.29) | 0.22 (-0.33, 0.77) |
| Honduras | 14.69 (8.81, 24.99) | 9.37 (5.58, 15.99) | 76.86 (39.64, 144.28) | 15.29 (7.89, 28.71) | 1.64 (1.48, 1.79) |
| France | 2138.64 (1778.03, 2471.71) | 26.70 (22.23, 30.86) | 2915.71 (2213.19, 3717.95) | 20.00 (15.26, 25.46) | -0.86 (-0.99, -0.73) |
| Myanmar | 277.94 (159.74, 490.43) | 15.82 (9.06, 27.89) | 936.88 (419.97, 1898.31) | 24.62 (11.13, 49.61) | 1.56 (1.42, 1.70) |
| Bahamas | 1.76 (1.50, 2.04) | 14.22 (12.13, 16.55) | 4.16 (3.32, 5.16) | 13.28 (10.61, 16.43) | -0.18 (-0.47, 0.10) |
| South Africa | 207.53 (120.03, 280.82) | 13.00 (7.50, 17.63) | 422.24 (336.91, 514.40) | 11.65 (9.26, 14.19) | -0.72 (-0.95, -0.50) |
| Guyana | 5.90 (4.95, 6.94) | 20.06 (16.82, 23.64) | 9.57 (7.38, 12.22) | 19.10 (14.79, 24.26) | 0.14 (-0.05, 0.32) |
| Bhutan | 2.11 (1.17, 3.39) | 13.05 (7.21, 21.06) | 8.56 (4.63, 13.90) | 17.07 (9.23, 27.71) | 0.84 (0.78, 0.89) |
| Hungary | 282.48 (236.22, 334.75) | 20.23 (16.95, 23.98) | 218.70 (167.38, 280.78) | 10.87 (8.33, 13.95) | -2.30 (-2.71, -1.90) |
| Philippines | 709.47 (558.50, 1030.18) | 32.92 (25.81, 48.00) | 2606.19 (2019.92, 3640.45) | 41.97 (32.43, 59.07) | 0.94 (0.72, 1.17) |
| Argentina | 1017.47 (798.35, 1281.83) | 35.09 (27.52, 44.21) | 1064.58 (862.48, 1306.71) | 20.21 (16.38, 24.80) | -1.83 (-2.09, -1.57) |
| North Macedonia | 59.48 (44.56, 78.42) | 40.47 (30.34, 53.36) | 115.58 (70.98, 175.79) | 37.39 (22.89, 56.83) | -0.24 (-0.43, -0.04) |
| Germany | 1170.38 (940.20, 1429.45) | 9.63 (7.74, 11.77) | 2027.04 (1598.69, 2507.35) | 10.65 (8.48, 13.13) | -0.02 (-0.18, 0.15) |
| Andorra | 0.13 (0.05, 0.28) | 2.56 (0.93, 5.58) | 0.28 (0.11, 0.60) | 2.00 (0.77, 4.32) | -0.69 (-0.85, -0.52) |
| Nicaragua | 16.83 (10.34, 25.13) | 14.64 (9.00, 21.88) | 72.01 (45.47, 113.00) | 18.43 (11.63, 28.93) | 0.97 (0.69, 1.25) |
| Greece | 993.13 (879.69, 1111.01) | 69.54 (61.51, 77.87) | 770.08 (652.21, 888.47) | 31.33 (26.80, 35.96) | -3.50 (-3.95, -3.04) |
| Chile | 293.35 (226.01, 369.40) | 35.09 (27.03, 44.18) | 511.10 (403.25, 629.76) | 21.90 (17.29, 26.97) | -1.40 (-1.60, -1.20) |
| Barbados | 5.71 (4.85, 6.60) | 18.77 (15.95, 21.69) | 8.67 (6.68, 10.69) | 17.75 (13.69, 21.87) | 0.27 (0.10, 0.44) |
| Azerbaijan | 40.31 (23.28, 73.58) | 11.63 (6.72, 21.22) | 80.15 (47.81, 140.25) | 11.28 (6.75, 19.77) | -0.02 (-0.08, 0.04) |
| Ecuador | 149.39 (116.98, 188.08) | 36.20 (28.30, 45.61) | 340.64 (250.45, 454.25) | 24.17 (17.79, 32.20) | -1.29 (-1.43, -1.15) |
| Haiti | 42.16 (23.66, 74.86) | 17.92 (9.88, 31.93) | 87.08 (44.80, 158.86) | 17.66 (9.05, 32.45) | -0.00 (-0.17, 0.16) |
| Brunei Darussalam | 0.31 (0.19, 0.50) | 4.15 (2.60, 6.88) | 1.05 (0.62, 1.76) | 4.25 (2.51, 7.21) | 0.71 (0.48, 0.93) |
| India | 3985.70 (2910.10, 4995.13) | 12.37 (9.01, 15.57) | 14642.57 (11707.45, 19059.11) | 15.00 (11.97, 19.49) | 0.52 (0.42, 0.61) |
| Japan | 1128.08 (1018.85, 1219.63) | 7.48 (6.73, 8.11) | 2130.61 (1790.17, 2387.48) | 5.57 (4.78, 6.17) | -0.75 (-1.04, -0.45) |
| Armenia | 35.99 (25.24, 48.93) | 19.25 (13.49, 26.19) | 88.16 (64.35, 119.86) | 22.32 (16.26, 30.33) | 0.87 (0.62, 1.11) |
| Zimbabwe | 61.20 (34.60, 91.39) | 20.00 (11.27, 30.08) | 94.42 (48.65, 152.88) | 19.44 (9.99, 31.30) | -0.06 (-0.24, 0.12) |
| Kazakhstan | 87.34 (58.90, 116.68) | 9.14 (6.15, 12.24) | 91.98 (64.91, 124.74) | 6.39 (4.51, 8.67) | -1.60 (-1.90, -1.31) |
| Georgia | 97.56 (65.69, 134.49) | 18.98 (12.78, 26.19) | 192.14 (138.80, 253.80) | 34.52 (24.95, 45.57) | 2.08 (1.50, 2.66) |
| Thailand | 484.15 (283.03, 736.79) | 19.35 (11.33, 29.40) | 3458.14 (2135.41, 5449.51) | 36.06 (22.29, 56.76) | 2.76 (2.50, 3.01) |
| Oman | 2.32 (1.26, 4.04) | 5.00 (2.71, 8.69) | 8.24 (5.08, 13.66) | 7.14 (4.39, 11.83) | 1.16 (1.00, 1.32) |
| Bulgaria | 217.08 (165.57, 272.54) | 19.06 (14.58, 23.88) | 277.10 (204.51, 364.68) | 18.58 (13.69, 24.50) | 0.51 (0.16, 0.86) |
| China | 9221.55 (5755.43, 16513.06) | 14.16 (8.85, 25.34) | 68141.69 (40148.51, 93656.44) | 34.82 (20.54, 47.79) | 3.86 (3.24, 4.49) |
| Denmark | 95.44 (77.96, 115.49) | 11.78 (9.63, 14.26) | 142.75 (109.23, 181.01) | 11.88 (9.09, 15.08) | -0.63 (-0.95, -0.32) |
| Uruguay | 19.27 (14.74, 24.83) | 5.19 (3.97, 6.69) | 24.08 (18.54, 31.08) | 4.51 (3.48, 5.83) | -0.57 (-0.94, -0.19) |
| Malaysia | 79.49 (44.91, 144.41) | 11.75 (6.63, 21.35) | 370.92 (176.98, 711.53) | 15.86 (7.55, 30.53) | 0.96 (0.82, 1.09) |
| Panama | 12.58 (10.89, 14.40) | 10.28 (8.89, 11.78) | 75.11 (57.03, 93.13) | 19.36 (14.71, 24.00) | 1.82 (1.52, 2.13) |
| Norway | 68.17 (60.72, 75.58) | 9.64 (8.59, 10.69) | 128.81 (109.86, 147.70) | 13.10 (11.20, 15.01) | 0.31 (-0.08, 0.70) |
| Poland | 1177.12 (1101.83, 1247.88) | 30.17 (28.18, 32.03) | 1144.37 (1005.19, 1287.06) | 15.97 (14.04, 17.96) | -2.35 (-2.63, -2.07) |
| Micronesia (Federated States of) | 0.39 (0.17, 0.87) | 9.64 (4.15, 21.61) | 0.58 (0.18, 1.36) | 12.66 (4.04, 29.53) | 0.84 (0.72, 0.96) |
| Ireland | 86.42 (70.09, 105.81) | 21.25 (17.20, 26.05) | 117.76 (88.22, 151.24) | 15.48 (11.60, 19.88) | -0.81 (-1.16, -0.45) |
| Estonia | 25.44 (20.65, 31.10) | 13.90 (11.29, 17.00) | 25.93 (19.59, 33.21) | 9.66 (7.29, 12.38) | -2.02 (-2.47, -1.57) |
| Croatia | 135.33 (110.57, 164.24) | 27.38 (22.40, 33.23) | 272.54 (212.50, 344.10) | 30.19 (23.53, 38.16) | -0.12 (-0.54, 0.31) |
| Israel | 75.26 (58.47, 95.86) | 16.15 (12.54, 20.56) | 147.51 (111.54, 187.68) | 12.25 (9.27, 15.58) | -1.09 (-1.27, -0.91) |
| Viet Nam | 663.11 (358.09, 1013.03) | 19.83 (10.73, 30.26) | 1983.54 (1084.37, 3057.70) | 26.03 (14.35, 40.22) | 0.92 (0.81, 1.04) |
| United States of America | 3451.52 (3117.77, 3720.61) | 10.99 (9.92, 11.85) | 7511.69 (6603.34, 8224.42) | 13.17 (11.58, 14.40) | 0.38 (0.19, 0.58) |
| Brazil | 1979.60 (1806.62, 2126.71) | 28.71 (26.05, 30.92) | 5352.28 (4769.66, 5826.81) | 24.80 (22.04, 27.02) | -0.24 (-0.41, -0.07) |
| Belarus | 233.46 (182.84, 288.53) | 21.15 (16.56, 26.15) | 206.50 (149.94, 276.20) | 13.93 (10.15, 18.58) | -1.36 (-1.56, -1.15) |
| Republic of Korea | 194.94 (124.39, 293.02) | 9.18 (5.85, 13.85) | 683.93 (412.54, 1014.77) | 8.07 (4.87, 11.98) | -0.70 (-1.01, -0.39) |
| Canada | 460.40 (388.37, 536.65) | 15.02 (12.64, 17.54) | 836.61 (653.12, 1030.82) | 11.72 (9.17, 14.43) | -0.60 (-0.74, -0.46) |
| Iceland | 4.97 (3.95, 6.08) | 18.35 (14.60, 22.44) | 11.52 (8.67, 14.65) | 20.51 (15.50, 26.08) | -0.40 (-0.77, -0.03) |
| Romania | 874.31 (637.71, 1153.71) | 36.10 (26.60, 47.29) | 1052.04 (811.89, 1345.09) | 27.86 (21.49, 35.63) | -0.84 (-0.96, -0.72) |
| Qatar | 0.68 (0.41, 1.15) | 15.46 (9.36, 26.06) | 6.97 (4.15, 11.78) | 20.26 (12.06, 34.56) | 1.27 (1.05, 1.49) |
| Portugal | 362.75 (306.30, 428.03) | 27.28 (22.97, 32.28) | 546.84 (419.50, 689.17) | 21.52 (16.56, 27.09) | -1.26 (-1.60, -0.91) |
| Montenegro | 9.69 (5.40, 15.27) | 19.32 (10.76, 30.41) | 17.10 (11.73, 25.45) | 18.46 (12.63, 27.51) | -0.22 (-0.41, -0.03) |
| Sri Lanka | 76.86 (42.02, 115.16) | 9.35 (5.13, 14.00) | 394.37 (197.00, 756.79) | 16.00 (8.01, 30.66) | 2.55 (2.16, 2.94) |
| Dominican Republic | 69.98 (34.23, 112.33) | 24.58 (12.01, 39.42) | 236.05 (122.72, 376.97) | 28.05 (14.59, 44.79) | 0.60 (0.45, 0.75) |
| Venezuela (Bolivarian Republic of) | 164.05 (140.77, 187.66) | 22.17 (18.98, 25.38) | 596.97 (446.41, 774.48) | 23.91 (17.92, 30.94) | 0.36 (0.16, 0.56) |
| Democratic People's Republic of Korea | 234.33 (133.82, 415.26) | 20.59 (11.86, 36.38) | 692.28 (336.60, 1377.53) | 25.48 (12.38, 50.63) | 1.03 (0.83, 1.23) |
| Yemen | 41.25 (20.45, 74.69) | 11.79 (5.79, 21.53) | 130.96 (68.21, 240.33) | 13.08 (6.80, 24.00) | 0.36 (0.30, 0.42) |
| Suriname | 3.68 (2.05, 5.61) | 19.08 (10.60, 29.05) | 8.75 (4.89, 14.26) | 17.00 (9.49, 27.69) | -0.05 (-0.15, 0.05) |
| Italy | 2651.41 (2369.48, 2896.09) | 30.84 (27.52, 33.74) | 2528.02 (2109.20, 2882.53) | 17.15 (14.51, 19.47) | -1.56 (-1.97, -1.15) |
| Indonesia | 975.05 (621.60, 1338.01) | 14.18 (9.08, 19.42) | 4498.04 (2019.26, 7175.11) | 25.59 (11.65, 40.61) | 1.98 (1.84, 2.13) |
| Egypt | 568.53 (315.83, 895.14) | 33.45 (18.37, 52.54) | 1601.34 (981.96, 2385.75) | 39.13 (23.44, 59.37) | 0.51 (0.38, 0.65) |
| Papua New Guinea | 6.70 (1.89, 18.80) | 5.78 (1.59, 16.16) | 22.04 (4.86, 61.28) | 7.13 (1.55, 19.91) | 0.64 (0.48, 0.81) |
| Latvia | 56.12 (45.29, 68.95) | 17.55 (14.17, 21.55) | 58.70 (44.41, 75.51) | 15.15 (11.46, 19.49) | -1.04 (-1.45, -0.62) |
| Serbia | 266.37 (154.89, 463.26) | 31.15 (18.01, 53.58) | 479.37 (297.86, 724.83) | 28.95 (17.98, 43.85) | -0.40 (-0.52, -0.29) |
| Cyprus | 15.11 (8.01, 23.06) | 19.51 (10.24, 30.16) | 38.71 (22.74, 58.03) | 19.45 (11.39, 29.24) | 0.20 (-0.03, 0.43) |
| Bosnia and Herzegovina | 47.92 (24.74, 89.83) | 16.43 (8.52, 30.83) | 96.60 (58.33, 160.51) | 15.98 (9.65, 26.52) | -0.03 (-0.10, 0.03) |
| Pakistan | 681.34 (459.91, 981.37) | 14.97 (10.09, 21.58) | 1542.28 (1004.43, 2260.82) | 18.26 (11.92, 26.75) | 0.56 (0.47, 0.64) |
| Tonga | 0.34 (0.13, 0.78) | 7.91 (3.12, 18.19) | 0.75 (0.23, 1.73) | 11.13 (3.40, 25.85) | 1.10 (0.93, 1.27) |
| Lesotho | 11.03 (6.25, 18.19) | 15.96 (9.03, 26.33) | 18.28 (9.87, 30.24) | 21.94 (11.80, 36.45) | 1.32 (1.15, 1.50) |
| Kyrgyzstan | 49.84 (36.93, 65.76) | 21.75 (16.10, 28.71) | 126.01 (89.66, 170.74) | 37.03 (26.56, 49.86) | 2.01 (1.49, 2.53) |
| Luxembourg | 9.28 (8.08, 10.58) | 18.25 (15.87, 20.82) | 15.89 (13.37, 18.67) | 16.16 (13.64, 18.96) | -0.37 (-0.56, -0.19) |
| Cambodia | 59.59 (34.26, 104.29) | 17.94 (10.33, 31.23) | 245.58 (106.74, 485.57) | 25.98 (11.39, 51.06) | 1.34 (1.26, 1.43) |
| Slovakia | 106.04 (72.93, 162.44) | 19.19 (13.20, 29.33) | 175.56 (107.17, 275.86) | 18.78 (11.46, 29.49) | -0.24 (-0.30, -0.18) |
| Samoa | 1.54 (0.65, 2.79) | 24.02 (10.13, 43.20) | 2.97 (1.43, 4.85) | 27.16 (13.03, 44.37) | 0.52 (0.38, 0.66) |
| Tunisia | 57.70 (32.06, 88.78) | 14.75 (8.24, 22.75) | 191.19 (101.93, 307.16) | 17.23 (9.23, 27.65) | 0.63 (0.58, 0.69) |
| Spain | 1345.67 (1155.33, 1551.86) | 25.63 (21.98, 29.59) | 1662.08 (1262.79, 2098.25) | 17.39 (13.29, 21.85) | -1.00 (-1.27, -0.73) |
| Saint Lucia | 1.27 (1.10, 1.46) | 15.91 (13.73, 18.39) | 2.55 (2.03, 3.12) | 12.38 (9.88, 15.14) | -1.00 (-1.18, -0.83) |
| Timor-Leste | 2.46 (1.40, 4.38) | 15.51 (8.82, 27.52) | 15.77 (6.78, 31.59) | 20.86 (8.99, 41.82) | 1.17 (1.07, 1.26) |
| Dominica | 1.22 (0.67, 1.89) | 21.32 (11.73, 33.06) | 1.89 (1.08, 2.94) | 26.73 (15.14, 41.55) | 0.91 (0.62, 1.19) |
| Lao People's Democratic Republic | 28.40 (15.74, 51.96) | 17.56 (9.76, 32.00) | 84.65 (36.26, 175.48) | 25.57 (11.08, 52.61) | 1.32 (1.19, 1.45) |
| Lithuania | 61.74 (50.17, 75.13) | 15.53 (12.62, 18.87) | 53.94 (41.34, 68.58) | 9.87 (7.56, 12.56) | -2.22 (-2.67, -1.77) |
| Burkina Faso | 36.19 (19.61, 69.36) | 10.63 (5.73, 20.29) | 65.57 (33.93, 112.56) | 9.46 (4.90, 16.17) | -0.47 (-0.58, -0.35) |
| Sweden | 219.35 (181.18, 262.87) | 13.92 (11.49, 16.70) | 186.77 (144.30, 235.40) | 8.37 (6.49, 10.55) | -1.27 (-1.54, -1.00) |
| Belgium | 324.52 (260.16, 400.20) | 21.91 (17.56, 27.05) | 420.63 (321.79, 530.60) | 18.22 (14.01, 22.97) | -0.69 (-1.05, -0.32) |
| United Arab Emirates | 5.13 (2.86, 9.29) | 23.88 (13.29, 43.68) | 28.57 (14.43, 48.01) | 24.96 (12.19, 42.23) | 2.19 (1.59, 2.79) |
| Albania | 58.24 (38.13, 82.40) | 36.69 (23.96, 52.10) | 133.92 (61.15, 234.30) | 32.15 (14.73, 56.14) | -0.44 (-0.55, -0.34) |
| Fiji | 2.91 (1.42, 4.78) | 12.35 (6.01, 20.40) | 8.63 (4.74, 13.51) | 15.39 (8.43, 24.14) | 0.68 (0.38, 0.98) |
| United Kingdom | 868.37 (800.50, 933.19) | 9.58 (8.83, 10.30) | 1625.93 (1438.12, 1781.49) | 12.54 (11.16, 13.70) | 1.09 (0.86, 1.33) |
| Cuba | 246.76 (194.15, 309.57) | 26.26 (20.65, 32.97) | 752.90 (581.69, 952.24) | 42.42 (32.77, 53.70) | 1.04 (0.78, 1.30) |
| Marshall Islands | 0.09 (0.03, 0.23) | 7.06 (2.37, 18.20) | 0.21 (0.06, 0.52) | 9.68 (2.76, 23.80) | 0.85 (0.66, 1.05) |
| Belize | 1.27 (0.94, 1.55) | 15.96 (11.84, 19.56) | 3.23 (2.70, 3.81) | 14.64 (12.24, 17.23) | -0.22 (-0.49, 0.04) |
| Slovenia | 32.37 (26.01, 39.79) | 14.90 (11.98, 18.28) | 59.38 (43.42, 78.67) | 13.48 (9.86, 17.88) | -0.42 (-0.83, -0.01) |
| Republic of Moldova | 112.06 (93.12, 131.92) | 30.13 (25.06, 35.40) | 77.62 (65.78, 90.68) | 13.89 (11.78, 16.22) | -2.96 (-3.43, -2.49) |
| Ukraine | 648.77 (500.59, 835.32) | 10.15 (7.83, 13.07) | 706.42 (501.01, 963.91) | 9.45 (6.70, 12.89) | -0.26 (-0.52, 0.01) |
| Austria | 139.81 (113.75, 170.02) | 11.95 (9.71, 14.55) | 250.42 (193.29, 316.95) | 14.16 (10.98, 17.91) | 0.10 (-0.21, 0.42) |
| Mongolia | 25.25 (14.27, 40.38) | 28.66 (16.21, 45.61) | 36.64 (22.58, 53.47) | 24.74 (15.16, 36.14) | -0.54 (-0.73, -0.36) |
| Netherlands | 249.80 (205.33, 300.34) | 12.98 (10.66, 15.61) | 456.45 (351.49, 572.39) | 12.97 (10.00, 16.26) | -0.29 (-0.50, -0.08) |
| Antigua and Barbuda | 0.71 (0.58, 0.84) | 13.32 (10.96, 15.83) | 1.26 (1.08, 1.46) | 14.42 (12.32, 16.74) | 0.50 (0.23, 0.78) |
| Malta | 8.97 (7.01, 11.26) | 22.87 (17.88, 28.70) | 18.73 (14.16, 24.15) | 18.48 (13.98, 23.84) | -0.86 (-1.09, -0.62) |
| Vanuatu | 0.30 (0.10, 0.75) | 7.04 (2.37, 17.67) | 1.16 (0.34, 2.86) | 9.72 (2.80, 24.03) | 1.01 (0.88, 1.14) |
| Singapore | 17.81 (14.02, 22.02) | 10.39 (8.18, 12.85) | 53.77 (40.48, 69.38) | 7.17 (5.39, 9.24) | -1.12 (-1.53, -0.72) |
| Turkmenistan | 43.29 (27.50, 62.07) | 30.82 (19.48, 44.28) | 72.73 (45.32, 111.52) | 25.38 (15.81, 39.00) | -0.81 (-0.90, -0.72) |
| Australia | 267.36 (220.89, 315.77) | 14.44 (11.91, 17.09) | 440.62 (339.14, 559.10) | 9.81 (7.57, 12.44) | -1.25 (-1.47, -1.03) |
| New Zealand | 58.15 (46.53, 71.69) | 15.28 (12.21, 18.86) | 90.18 (69.25, 114.35) | 11.06 (8.50, 14.02) | -1.27 (-1.58, -0.95) |
| Czechia | 231.47 (194.19, 273.65) | 17.67 (14.82, 20.90) | 315.06 (246.36, 398.99) | 14.20 (11.09, 18.00) | -0.81 (-1.19, -0.43) |
| Tajikistan | 64.45 (37.02, 103.92) | 31.67 (18.24, 51.01) | 80.97 (47.28, 124.05) | 21.13 (12.41, 32.41) | -1.74 (-2.07, -1.40) |
| Russian Federation | 5903.29 (5568.31, 6209.99) | 39.19 (36.90, 41.28) | 2482.46 (2213.87, 2754.68) | 10.83 (9.66, 12.02) | -5.10 (-5.47, -4.72) |
| Botswana | 7.00 (4.16, 11.68) | 16.97 (10.05, 28.21) | 18.69 (10.86, 30.03) | 18.34 (10.61, 29.59) | 0.51 (0.33, 0.70) |
| Namibia | 10.28 (5.49, 15.38) | 20.90 (11.07, 31.57) | 21.44 (10.37, 34.92) | 21.44 (10.42, 35.01) | -0.16 (-0.38, 0.06) |
| Kiribati | 0.17 (0.10, 0.28) | 6.49 (3.68, 10.74) | 0.37 (0.20, 0.68) | 8.34 (4.54, 15.20) | 0.94 (0.70, 1.19) |
| Bolivia (Plurinational State of) | 57.73 (33.18, 89.96) | 23.92 (13.76, 37.19) | 199.59 (104.74, 331.84) | 27.20 (14.36, 45.13) | 0.38 (0.31, 0.45) |
| Maldives | 0.96 (0.58, 1.67) | 16.82 (10.05, 29.51) | 3.55 (2.02, 6.40) | 16.18 (9.19, 29.16) | -0.18 (-0.32, -0.05) |
| Grenada | 1.67 (1.14, 2.10) | 23.90 (16.44, 30.00) | 1.64 (1.35, 1.94) | 16.96 (13.90, 20.17) | -0.69 (-1.14, -0.24) |
| Finland | 87.19 (69.42, 107.11) | 12.66 (10.07, 15.56) | 80.22 (60.89, 102.26) | 6.11 (4.66, 7.77) | -2.41 (-2.57, -2.24) |
| Afghanistan | 102.22 (56.99, 175.80) | 18.85 (10.41, 32.40) | 141.23 (80.29, 235.77) | 22.31 (12.69, 37.17) | 0.63 (0.48, 0.78) |
| Switzerland | 120.34 (97.17, 146.61) | 11.86 (9.59, 14.45) | 173.93 (127.06, 227.14) | 9.76 (7.16, 12.72) | -0.81 (-1.03, -0.59) |
| Bangladesh | 482.91 (282.62, 846.04) | 13.64 (7.98, 23.90) | 1647.61 (926.84, 2798.73) | 14.28 (8.03, 24.20) | 0.01 (-0.05, 0.06) |
| Solomon Islands | 0.59 (0.18, 1.60) | 6.66 (1.99, 18.02) | 1.99 (0.56, 5.12) | 8.79 (2.45, 22.70) | 0.85 (0.73, 0.97) |
