# Supplementary material for: Global, regional, and national burden of malignant neoplasm of bone and articular cartilage in adults aged 65 years and older, 1990–2021: a systematic analysis based on the global burden of disease study 2021
Source: Aging Clin Exp Res. 2025 Jan 8;37(1):21. doi: 10.1007/s40520-024-02926-0 (PMC11711276; doi:10.1007/s40520-024-02926-0)
Supplement: Supplementary file 6 — Supplementary file6 (DOCX 46 KB) [file 40520_2024_2926_MOESM6_ESM.docx]

Table S3 MNBAC incidence in people aged ≥65 years in 1990 and 2021 for both sexes and EAPC in age-standardized rates by location

| location | Number in 1990 (95% CI) | Rate in 1990 (95% CI) | Number in 2021 (95% CI) | Rate in 2021 (95% CI) | EAPC in age-standardized rates between 1990 and 2021 (95%CI) |
| --- | --- | --- | --- | --- | --- |
| Global | 10112.65 (9140.38, 11786.43) | 3.18 (2.86, 3.70) | 28100.56 (21417.77, 33168.44) | 3.69 (2.82, 4.36) | 0.64 (0.55, 0.73) |
| High SDI | 2427.93 (2210.87, 2613.82) | 2.34 (2.13, 2.53) | 4494.00 (3841.36, 5066.71) | 2.13 (1.83, 2.39) | -0.41 (-0.52, -0.31) |
| High-middle SDI | 3631.58 (3279.50, 4123.28) | 4.48 (4.02, 5.08) | 8456.31 (5653.73, 11066.58) | 4.66 (3.12, 6.09) | 0.34 (0.20, 0.48) |
| Middle SDI | 2280.39 (1839.47, 3205.01) | 3.10 (2.50, 4.31) | 10401.61 (7595.58, 12745.64) | 4.63 (3.39, 5.67) | 1.71 (1.46, 1.96) |
| Low-middle SDI | 1247.31 (963.69, 1500.10) | 2.90 (2.25, 3.50) | 3655.89 (2937.93, 4364.85) | 3.28 (2.64, 3.92) | 0.37 (0.34, 0.40) |
| Low SDI | 509.18 (390.20, 636.48) | 3.19 (2.44, 4.01) | 1066.02 (834.69, 1412.12) | 2.98 (2.33, 3.94) | -0.32 (-0.40, -0.24) |
| Eastern Sub-Saharan Africa | 256.21 (199.41, 337.30) | 4.82 (3.74, 6.35) | 486.27 (353.61, 719.75) | 4.25 (3.09, 6.26) | -0.56 (-0.63, -0.50) |
| Western Sub-Saharan Africa | 145.29 (101.82, 191.74) | 2.22 (1.56, 2.93) | 276.67 (208.28, 362.67) | 2.10 (1.58, 2.75) | -0.23 (-0.28, -0.18) |
| Central Sub-Saharan Africa | 45.24 (28.15, 71.11) | 3.21 (1.97, 5.09) | 85.11 (48.07, 130.77) | 2.57 (1.44, 3.97) | -0.84 (-0.96, -0.72) |
| North Africa and Middle East | 394.23 (296.99, 525.25) | 3.39 (2.55, 4.53) | 1156.70 (901.89, 1550.66) | 3.56 (2.77, 4.78) | 0.24 (0.14, 0.34) |
| Oceania | 2.71 (1.53, 5.34) | 1.48 (0.85, 2.87) | 7.62 (3.66, 15.36) | 1.63 (0.79, 3.28) | 0.36 (0.17, 0.55) |
| South Asia | 988.33 (730.51, 1225.20) | 2.52 (1.86, 3.14) | 3193.68 (2562.81, 4124.17) | 2.74 (2.20, 3.54) | 0.12 (0.03, 0.21) |
| Southeast Asia | 599.98 (476.85, 752.42) | 3.30 (2.61, 4.15) | 2509.23 (1641.73, 3251.32) | 5.00 (3.30, 6.46) | 1.59 (1.44, 1.73) |
| Caribbean | 85.34 (71.81, 100.96) | 3.86 (3.24, 4.57) | 236.13 (191.61, 284.42) | 4.96 (4.03, 5.97) | 0.65 (0.45, 0.84) |
| Southern Sub-Saharan Africa | 53.39 (36.42, 68.93) | 2.62 (1.78, 3.40) | 107.88 (87.64, 135.58) | 2.52 (2.03, 3.15) | -0.41 (-0.71, -0.10) |
| Central Latin America | 218.25 (203.00, 231.60) | 3.52 (3.26, 3.74) | 782.04 (686.43, 876.29) | 3.75 (3.29, 4.20) | 0.41 (0.13, 0.68) |
| Central Asia | 111.73 (85.43, 136.70) | 3.19 (2.44, 3.91) | 208.86 (174.69, 246.00) | 3.49 (2.92, 4.11) | 0.35 (0.14, 0.56) |
| Andean Latin America | 67.48 (52.81, 86.83) | 4.34 (3.39, 5.58) | 191.86 (139.76, 261.80) | 3.87 (2.82, 5.27) | -0.49 (-0.59, -0.39) |
| Tropical Latin America | 357.46 (325.71, 384.65) | 5.20 (4.70, 5.62) | 933.80 (828.38, 1017.44) | 4.25 (3.76, 4.63) | -0.40 (-0.57, -0.24) |
| Central Europe | 644.89 (568.21, 721.65) | 4.99 (4.39, 5.58) | 767.99 (664.72, 877.44) | 3.44 (2.98, 3.93) | -1.38 (-1.56, -1.19) |
| Southern Latin America | 234.52 (191.94, 284.21) | 5.80 (4.74, 7.03) | 275.77 (231.45, 325.01) | 3.38 (2.84, 3.98) | -1.74 (-1.97, -1.50) |
| East Asia | 1749.13 (1127.19, 3045.69) | 2.74 (1.77, 4.77) | 11762.22 (6955.14, 16116.70) | 5.96 (3.53, 8.16) | 3.43 (2.82, 4.04) |
| Eastern Europe | 1262.58 (1181.26, 1334.89) | 5.38 (5.02, 5.70) | 626.04 (553.06, 699.99) | 1.86 (1.65, 2.09) | -4.27 (-4.57, -3.97) |
| Western Europe | 1931.25 (1743.40, 2102.25) | 3.46 (3.11, 3.77) | 2477.03 (2085.65, 2824.43) | 2.56 (2.19, 2.91) | -1.03 (-1.17, -0.90) |
| High-income Asia Pacific | 227.46 (202.35, 252.84) | 1.34 (1.18, 1.49) | 479.97 (388.16, 564.95) | 0.98 (0.80, 1.15) | -0.88 (-1.11, -0.65) |
| High-income North America | 680.68 (611.60, 738.55) | 1.98 (1.78, 2.15) | 1445.75 (1255.36, 1596.77) | 2.23 (1.94, 2.46) | 0.19 (0.01, 0.38) |
| Australasia | 56.49 (47.77, 65.96) | 2.57 (2.17, 3.01) | 89.95 (70.68, 111.77) | 1.67 (1.32, 2.08) | -1.42 (-1.62, -1.23) |
| Mexico | 115.66 (109.34, 121.25) | 3.69 (3.48, 3.88) | 417.81 (366.14, 469.48) | 4.05 (3.55, 4.55) | 0.91 (0.40, 1.43) |
| Guatemala | 9.58 (7.86, 11.26) | 4.45 (3.58, 5.26) | 30.65 (25.39, 36.88) | 3.44 (2.85, 4.13) | -0.76 (-0.99, -0.54) |
| Guinea | 6.13 (3.48, 9.42) | 2.25 (1.28, 3.46) | 9.51 (4.90, 15.74) | 2.28 (1.18, 3.75) | -0.01 (-0.05, 0.04) |
| Gambia | 0.51 (0.29, 0.84) | 2.03 (1.15, 3.30) | 1.61 (0.84, 2.60) | 2.27 (1.18, 3.66) | 0.16 (0.05, 0.27) |
| El Salvador | 4.66 (3.35, 6.20) | 1.92 (1.38, 2.55) | 14.11 (8.79, 21.30) | 2.49 (1.55, 3.75) | 0.84 (0.78, 0.91) |
| Costa Rica | 3.49 (2.79, 4.34) | 2.44 (1.95, 3.03) | 16.73 (12.58, 21.52) | 3.49 (2.63, 4.49) | 1.48 (1.24, 1.72) |
| Cabo Verde | 0.23 (0.13, 0.41) | 1.04 (0.58, 1.88) | 0.38 (0.21, 0.68) | 1.19 (0.64, 2.15) | 0.29 (0.17, 0.42) |
| Peru | 30.18 (19.07, 47.10) | 3.27 (2.06, 5.10) | 96.77 (58.56, 152.58) | 3.38 (2.05, 5.33) | -0.20 (-0.38, -0.01) |
| Paraguay | 9.58 (5.72, 14.13) | 5.38 (3.21, 7.95) | 28.45 (16.71, 43.93) | 6.04 (3.55, 9.32) | 0.47 (0.26, 0.67) |
| Guinea-Bissau | 0.64 (0.35, 1.16) | 2.26 (1.23, 4.05) | 0.86 (0.47, 1.46) | 1.94 (1.05, 3.27) | -0.54 (-0.57, -0.50) |
| Colombia | 49.03 (41.20, 57.84) | 3.76 (3.15, 4.44) | 161.86 (123.65, 205.47) | 3.35 (2.57, 4.25) | -0.97 (-1.17, -0.76) |
| Sao Tome and Principe | 0.09 (0.05, 0.16) | 1.65 (0.94, 3.00) | 0.13 (0.07, 0.22) | 1.77 (0.99, 2.87) | 0.11 (0.04, 0.19) |
| Saint Vincent and the Grenadines | 0.12 (0.10, 0.14) | 1.85 (1.60, 2.10) | 0.76 (0.64, 0.89) | 6.21 (5.23, 7.32) | 3.38 (2.11, 4.66) |
| Trinidad and Tobago | 2.56 (2.23, 2.94) | 3.56 (3.10, 4.10) | 4.39 (3.31, 5.61) | 2.52 (1.90, 3.21) | -1.12 (-1.31, -0.94) |
| Jamaica | 8.29 (6.19, 10.69) | 4.87 (3.63, 6.27) | 12.62 (8.95, 17.31) | 4.70 (3.33, 6.45) | 0.04 (-0.25, 0.32) |
| Turkey | 79.92 (49.36, 126.17) | 3.32 (2.05, 5.24) | 250.67 (146.49, 376.88) | 3.20 (1.88, 4.81) | 0.01 (-0.34, 0.37) |
| Bermuda | 0.14 (0.11, 0.19) | 2.70 (2.00, 3.53) | 0.32 (0.24, 0.44) | 2.38 (1.75, 3.26) | -0.31 (-0.52, -0.09) |
| Saudi Arabia | 11.71 (6.62, 18.64) | 3.07 (1.74, 4.88) | 33.16 (18.91, 52.47) | 3.59 (2.07, 5.68) | 0.43 (0.36, 0.51) |
| Burundi | 6.97 (3.64, 10.98) | 3.89 (2.03, 6.10) | 10.71 (4.80, 18.29) | 3.42 (1.55, 5.84) | -0.59 (-0.66, -0.52) |
| Morocco | 20.69 (11.78, 33.68) | 1.86 (1.06, 3.02) | 55.94 (32.28, 92.12) | 2.12 (1.22, 3.47) | 0.55 (0.43, 0.67) |
| Palestine | 3.10 (1.64, 4.82) | 4.73 (2.51, 7.37) | 8.98 (5.71, 13.90) | 5.45 (3.46, 8.45) | 0.71 (0.56, 0.86) |
| Bahrain | 0.49 (0.31, 0.82) | 5.21 (3.28, 8.74) | 2.63 (1.58, 4.44) | 5.89 (3.53, 10.04) | 0.32 (0.18, 0.45) |
| Algeria | 30.94 (18.64, 48.15) | 3.63 (2.21, 5.72) | 93.53 (48.05, 150.72) | 3.65 (1.87, 5.90) | 0.10 (-0.03, 0.23) |
| Central African Republic | 2.15 (1.14, 3.83) | 3.13 (1.65, 5.59) | 2.99 (1.42, 4.86) | 2.54 (1.18, 4.15) | -0.75 (-0.80, -0.70) |
| Syrian Arab Republic | 3.33 (1.73, 6.45) | 0.93 (0.49, 1.81) | 9.84 (5.10, 18.55) | 1.07 (0.55, 2.05) | 0.33 (0.25, 0.40) |
| Equatorial Guinea | 0.38 (0.20, 0.70) | 3.00 (1.51, 5.48) | 0.94 (0.46, 1.58) | 2.94 (1.45, 4.93) | 0.10 (0.01, 0.18) |
| Iran (Islamic Republic of) | 38.88 (30.38, 50.03) | 2.33 (1.81, 2.98) | 144.50 (110.43, 192.37) | 2.49 (1.90, 3.32) | 0.30 (0.23, 0.38) |
| Jordan | 2.96 (1.71, 4.54) | 3.59 (2.06, 5.51) | 20.39 (12.05, 33.06) | 4.24 (2.50, 6.88) | 0.73 (0.58, 0.87) |
| Nepal | 14.73 (8.61, 23.89) | 2.34 (1.37, 3.81) | 48.32 (27.46, 77.17) | 2.63 (1.50, 4.20) | 0.42 (0.27, 0.58) |
| Congo | 2.60 (1.46, 4.17) | 3.54 (1.99, 5.74) | 5.08 (2.61, 8.17) | 3.12 (1.61, 5.00) | -0.59 (-0.68, -0.50) |
| Democratic Republic of the Congo | 31.90 (17.31, 54.62) | 3.26 (1.74, 5.68) | 55.81 (26.29, 92.11) | 2.50 (1.17, 4.16) | -0.97 (-1.12, -0.83) |
| Libya | 3.42 (1.88, 5.66) | 2.50 (1.37, 4.13) | 10.38 (5.77, 17.10) | 3.02 (1.68, 4.96) | 0.99 (0.85, 1.12) |
| Comoros | 0.59 (0.30, 0.94) | 4.23 (2.13, 6.67) | 1.49 (0.66, 2.70) | 4.13 (1.84, 7.45) | -0.19 (-0.25, -0.14) |
| Lebanon | 5.39 (3.11, 8.38) | 3.43 (1.98, 5.33) | 23.15 (12.81, 35.57) | 4.08 (2.25, 6.26) | 0.82 (0.61, 1.03) |
| Kuwait | 0.86 (0.65, 1.09) | 2.59 (1.96, 3.29) | 5.57 (3.93, 7.63) | 3.36 (2.38, 4.61) | 1.66 (0.28, 3.06) |
| Gabon | 1.51 (0.80, 2.55) | 3.35 (1.77, 5.70) | 2.25 (1.14, 3.71) | 3.17 (1.62, 5.22) | -0.37 (-0.49, -0.25) |
| Sudan | 18.14 (9.86, 31.04) | 2.51 (1.35, 4.31) | 36.76 (21.70, 62.19) | 2.74 (1.62, 4.65) | 0.32 (0.25, 0.39) |
| Djibouti | 0.29 (0.14, 0.49) | 3.77 (1.87, 6.28) | 1.63 (0.73, 2.95) | 4.28 (1.91, 7.61) | 0.39 (0.32, 0.46) |
| Eritrea | 2.18 (1.07, 3.38) | 3.91 (1.93, 6.10) | 6.48 (2.85, 11.26) | 4.01 (1.77, 6.98) | -0.02 (-0.07, 0.03) |
| Kenya | 25.95 (19.30, 33.83) | 4.30 (3.19, 5.60) | 75.02 (54.99, 105.95) | 4.74 (3.47, 6.66) | 0.40 (0.36, 0.44) |
| Iraq | 28.43 (15.19, 44.06) | 4.62 (2.47, 7.17) | 82.50 (48.81, 126.06) | 5.14 (3.05, 7.81) | 0.43 (0.33, 0.54) |
| Malawi | 10.74 (5.35, 16.35) | 3.93 (1.97, 6.00) | 18.91 (9.06, 32.12) | 3.64 (1.74, 6.19) | -0.32 (-0.38, -0.25) |
| Angola | 6.71 (3.41, 12.23) | 2.93 (1.48, 5.39) | 18.04 (8.63, 29.47) | 2.59 (1.24, 4.26) | -0.50 (-0.60, -0.40) |
| Seychelles | 0.05 (0.01, 0.10) | 0.89 (0.25, 1.97) | 0.08 (0.02, 0.17) | 0.88 (0.21, 1.93) | 0.12 (-0.30, 0.54) |
| Madagascar | 13.83 (7.20, 21.70) | 3.77 (1.97, 5.89) | 20.10 (9.00, 34.18) | 3.06 (1.38, 5.18) | -0.60 (-0.78, -0.42) |
| Ethiopia | 87.89 (61.64, 123.82) | 6.47 (4.50, 9.16) | 155.27 (105.77, 214.96) | 4.92 (3.35, 6.81) | -1.21 (-1.35, -1.08) |
| Mozambique | 17.49 (8.63, 27.09) | 4.27 (2.11, 6.63) | 29.88 (13.70, 49.51) | 4.09 (1.87, 6.83) | -0.03 (-0.11, 0.05) |
| Rwanda | 8.94 (4.55, 13.65) | 4.53 (2.30, 6.90) | 15.67 (7.02, 28.36) | 3.65 (1.65, 6.59) | -1.05 (-1.19, -0.90) |
| Uganda | 22.19 (13.24, 33.33) | 4.64 (2.76, 6.97) | 42.03 (22.22, 67.79) | 4.26 (2.26, 6.88) | -0.61 (-0.75, -0.46) |
| Mauritius | 2.94 (2.59, 3.32) | 5.09 (4.47, 5.73) | 7.62 (6.57, 8.72) | 4.66 (4.01, 5.33) | 1.08 (-0.43, 2.61) |
| Somalia | 5.50 (2.68, 8.79) | 4.36 (2.12, 6.95) | 14.25 (5.92, 23.97) | 3.83 (1.59, 6.53) | -0.37 (-0.41, -0.34) |
| United Republic of Tanzania | 35.52 (18.34, 54.77) | 4.38 (2.28, 6.75) | 67.26 (30.79, 117.67) | 3.73 (1.72, 6.51) | -0.66 (-0.72, -0.60) |
| Eswatini | 0.85 (0.49, 1.38) | 4.46 (2.57, 7.19) | 1.79 (0.91, 3.04) | 4.77 (2.40, 8.15) | 0.29 (-0.05, 0.63) |
| Zambia | 8.45 (4.27, 12.95) | 4.37 (2.23, 6.71) | 16.75 (7.40, 29.71) | 3.77 (1.69, 6.66) | -0.67 (-0.75, -0.59) |
| Chad | 4.19 (2.22, 7.82) | 1.75 (0.92, 3.26) | 7.58 (3.58, 13.75) | 1.89 (0.90, 3.41) | 0.22 (0.18, 0.26) |
| Benin | 3.31 (1.93, 5.85) | 2.05 (1.20, 3.62) | 6.66 (3.63, 11.16) | 1.84 (1.00, 3.08) | -0.46 (-0.53, -0.38) |
| Coted'Ivoire | 4.16 (2.48, 7.31) | 1.79 (1.07, 3.12) | 11.37 (6.54, 19.44) | 1.63 (0.94, 2.77) | -0.35 (-0.41, -0.29) |
| Ghana | 7.30 (3.97, 13.87) | 1.71 (0.94, 3.24) | 22.35 (11.97, 37.42) | 1.95 (1.05, 3.27) | 0.44 (0.41, 0.47) |
| Cameroon | 7.89 (4.81, 13.46) | 2.61 (1.58, 4.43) | 18.22 (9.73, 31.32) | 2.19 (1.17, 3.74) | -0.64 (-0.70, -0.58) |
| Liberia | 2.26 (1.33, 4.22) | 2.35 (1.37, 4.37) | 2.54 (1.39, 4.32) | 1.91 (1.05, 3.24) | -0.58 (-0.73, -0.43) |
| Niger | 3.74 (2.02, 6.83) | 2.05 (1.10, 3.72) | 9.94 (5.00, 17.17) | 1.78 (0.89, 3.06) | -0.54 (-0.59, -0.48) |
| Mali | 5.43 (3.26, 9.04) | 1.95 (1.18, 3.25) | 11.16 (6.28, 18.59) | 1.82 (1.03, 3.03) | -0.21 (-0.26, -0.16) |
| Sierra Leone | 2.97 (1.57, 5.56) | 1.75 (0.93, 3.28) | 4.34 (2.29, 7.64) | 1.59 (0.84, 2.78) | -0.41 (-0.48, -0.35) |
| Senegal | 5.14 (2.91, 9.03) | 2.08 (1.18, 3.65) | 11.15 (5.80, 18.90) | 1.96 (1.02, 3.31) | -0.21 (-0.29, -0.13) |
| Cook Islands | 0.01 (0.00, 0.02) | 0.91 (0.51, 1.65) | 0.02 (0.01, 0.04) | 0.85 (0.45, 1.68) | -0.36 (-0.48, -0.23) |
| Greenland | 0.04 (0.02, 0.06) | 1.73 (0.84, 3.00) | 0.05 (0.03, 0.07) | 0.90 (0.50, 1.45) | -1.91 (-2.33, -1.47) |
| Guam | 0.05 (0.03, 0.09) | 1.12 (0.68, 1.79) | 0.12 (0.08, 0.19) | 0.66 (0.41, 0.99) | -1.28 (-1.49, -1.07) |
| Niue | 0.00 (0.00, 0.01) | 1.81 (0.75, 4.17) | 0.00 (0.00, 0.01) | 2.21 (0.75, 5.05) | 0.63 (0.52, 0.75) |
| Palau | 0.00 (0.00, 0.01) | 0.33 (0.13, 0.76) | 0.01 (0.00, 0.01) | 0.34 (0.14, 0.79) | 0.36 (0.20, 0.53) |
| Nigeria | 80.91 (55.09, 113.38) | 2.40 (1.64, 3.35) | 138.98 (100.38, 195.34) | 2.33 (1.69, 3.25) | -0.13 (-0.23, -0.03) |
| Northern Mariana Islands | 0.01 (0.01, 0.02) | 1.52 (0.81, 2.55) | 0.06 (0.03, 0.09) | 1.58 (0.96, 2.62) | 0.62 (0.16, 1.09) |
| Mauritania | 1.91 (1.12, 3.29) | 2.29 (1.35, 3.94) | 3.58 (1.86, 6.18) | 2.18 (1.14, 3.76) | -0.36 (-0.42, -0.31) |
| Togo | 1.59 (0.91, 2.87) | 1.89 (1.08, 3.40) | 4.21 (2.27, 7.35) | 1.73 (0.94, 3.00) | -0.46 (-0.52, -0.39) |
| Saint Kitts and Nevis | 0.20 (0.17, 0.23) | 5.10 (4.38, 5.88) | 0.18 (0.14, 0.21) | 3.69 (3.01, 4.43) | -0.48 (-0.84, -0.12) |
| American Samoa | 0.04 (0.02, 0.07) | 2.90 (1.54, 4.65) | 0.13 (0.08, 0.21) | 3.53 (2.14, 5.83) | 1.04 (0.76, 1.31) |
| Puerto Rico | 3.90 (3.02, 4.94) | 1.17 (0.91, 1.48) | 16.39 (12.09, 21.43) | 2.20 (1.63, 2.87) | 2.35 (1.00, 3.71) |
| Monaco | 0.03 (0.01, 0.07) | 0.42 (0.14, 0.91) | 0.05 (0.02, 0.09) | 0.44 (0.17, 0.89) | -0.12 (-0.24, 0.00) |
| Nauru | 0.01 (0.00, 0.01) | 2.01 (0.83, 4.14) | 0.01 (0.00, 0.02) | 2.54 (0.83, 5.62) | 0.67 (0.55, 0.78) |
| Tokelau | 0.00 (0.00, 0.00) | 1.67 (0.61, 4.07) | 0.00 (0.00, 0.01) | 2.00 (0.62, 4.66) | 0.61 (0.51, 0.72) |
| San Marino | 0.14 (0.07, 0.21) | 3.97 (2.16, 6.24) | 0.16 (0.08, 0.28) | 2.05 (0.97, 3.59) | -1.48 (-1.82, -1.15) |
| South Sudan | 9.49 (4.47, 15.37) | 4.50 (2.13, 7.28) | 10.40 (4.32, 19.25) | 4.43 (1.85, 8.14) | -0.12 (-0.15, -0.09) |
| Tuvalu | 0.01 (0.00, 0.02) | 1.61 (0.57, 4.03) | 0.02 (0.01, 0.04) | 2.00 (0.60, 4.90) | 0.68 (0.55, 0.81) |
| United States Virgin Islands | 0.06 (0.04, 0.10) | 1.04 (0.64, 1.71) | 0.17 (0.08, 0.31) | 0.90 (0.45, 1.71) | 0.01 (-0.17, 0.19) |
| Taiwan (Province of China) | 54.18 (45.54, 63.37) | 4.25 (3.55, 4.99) | 90.82 (69.34, 115.42) | 2.31 (1.76, 2.93) | -1.60 (-2.07, -1.12) |
| Uzbekistan | 31.97 (15.80, 48.41) | 3.70 (1.85, 5.59) | 74.06 (53.78, 98.21) | 4.09 (2.95, 5.43) | 0.57 (0.08, 1.06) |
| Honduras | 2.51 (1.50, 4.27) | 1.64 (0.97, 2.80) | 13.57 (7.00, 25.55) | 2.79 (1.44, 5.26) | 1.80 (1.62, 1.99) |
| France | 374.23 (310.70, 433.15) | 4.68 (3.89, 5.42) | 511.75 (385.56, 654.64) | 3.36 (2.55, 4.29) | -1.01 (-1.15, -0.87) |
| Myanmar | 51.85 (29.89, 91.43) | 3.05 (1.75, 5.39) | 158.32 (71.14, 319.65) | 4.24 (1.92, 8.51) | 1.13 (0.98, 1.27) |
| Bahamas | 0.31 (0.26, 0.36) | 2.52 (2.14, 2.93) | 0.71 (0.57, 0.88) | 2.30 (1.84, 2.84) | -0.26 (-0.55, 0.02) |
| South Africa | 36.17 (20.88, 48.98) | 2.31 (1.33, 3.14) | 77.38 (61.48, 94.36) | 2.21 (1.74, 2.70) | -0.51 (-0.82, -0.19) |
| Guyana | 1.15 (0.96, 1.35) | 4.01 (3.35, 4.72) | 1.72 (1.32, 2.19) | 3.51 (2.72, 4.46) | -0.09 (-0.27, 0.09) |
| Bhutan | 0.38 (0.21, 0.61) | 2.45 (1.35, 3.95) | 1.51 (0.81, 2.45) | 3.05 (1.64, 4.96) | 0.68 (0.65, 0.72) |
| Hungary | 51.99 (43.43, 61.73) | 3.81 (3.18, 4.53) | 37.92 (28.97, 48.73) | 1.88 (1.44, 2.42) | -2.55 (-2.95, -2.16) |
| Philippines | 122.36 (96.09, 177.45) | 5.91 (4.62, 8.63) | 450.80 (348.17, 631.95) | 7.42 (5.70, 10.50) | 0.94 (0.70, 1.18) |
| Argentina | 179.17 (140.47, 225.54) | 6.28 (4.92, 7.91) | 185.38 (149.80, 227.86) | 3.52 (2.84, 4.32) | -1.89 (-2.16, -1.63) |
| North Macedonia | 10.94 (8.17, 14.48) | 7.59 (5.66, 10.04) | 20.39 (12.50, 30.95) | 7.03 (4.28, 10.69) | -0.29 (-0.54, -0.03) |
| Germany | 210.09 (168.23, 256.35) | 1.74 (1.39, 2.12) | 348.57 (272.38, 433.32) | 1.79 (1.41, 2.22) | -0.25 (-0.41, -0.09) |
| Andorra | 0.02 (0.01, 0.05) | 0.44 (0.16, 0.96) | 0.05 (0.02, 0.10) | 0.34 (0.13, 0.73) | -0.74 (-0.90, -0.58) |
| Nicaragua | 2.81 (1.73, 4.19) | 2.48 (1.52, 3.69) | 11.88 (7.49, 18.67) | 3.09 (1.94, 4.86) | 0.97 (0.68, 1.26) |
| Greece | 172.44 (152.28, 193.29) | 12.28 (10.81, 13.81) | 136.77 (114.70, 158.95) | 5.36 (4.54, 6.19) | -3.63 (-4.08, -3.18) |
| Chile | 51.89 (40.01, 65.29) | 6.33 (4.88, 7.97) | 86.15 (67.60, 106.58) | 3.69 (2.89, 4.56) | -1.62 (-1.83, -1.41) |
| Barbados | 1.01 (0.86, 1.17) | 3.36 (2.85, 3.89) | 1.52 (1.18, 1.88) | 3.15 (2.43, 3.88) | 0.22 (0.05, 0.39) |
| Azerbaijan | 7.18 (4.14, 13.10) | 2.08 (1.20, 3.79) | 14.53 (8.69, 25.42) | 2.09 (1.25, 3.67) | 0.12 (0.04, 0.20) |
| Ecuador | 26.60 (20.76, 33.59) | 6.68 (5.19, 8.45) | 59.62 (43.95, 79.22) | 4.35 (3.21, 5.76) | -1.26 (-1.40, -1.13) |
| Haiti | 8.00 (4.47, 14.22) | 3.57 (1.95, 6.39) | 15.82 (8.13, 28.90) | 3.35 (1.71, 6.19) | -0.10 (-0.17, -0.04) |
| Brunei Darussalam | 0.06 (0.04, 0.10) | 0.83 (0.52, 1.39) | 0.18 (0.11, 0.30) | 0.77 (0.45, 1.32) | 0.52 (0.27, 0.77) |
| India | 756.22 (550.85, 951.81) | 2.46 (1.78, 3.11) | 2586.43 (2064.20, 3364.88) | 2.72 (2.17, 3.53) | 0.18 (0.07, 0.29) |
| Japan | 189.51 (170.36, 205.48) | 1.28 (1.14, 1.40) | 358.81 (297.54, 405.97) | 0.90 (0.77, 1.00) | -0.93 (-1.23, -0.62) |
| Armenia | 6.42 (4.49, 8.74) | 3.45 (2.41, 4.69) | 15.08 (10.99, 20.51) | 3.83 (2.79, 5.21) | 0.66 (0.41, 0.91) |
| Zimbabwe | 11.11 (6.29, 16.58) | 3.80 (2.14, 5.72) | 17.71 (9.14, 28.58) | 3.86 (1.98, 6.20) | 0.07 (-0.25, 0.39) |
| Kazakhstan | 15.72 (10.52, 21.06) | 1.66 (1.11, 2.23) | 16.03 (11.30, 21.75) | 1.14 (0.80, 1.55) | -1.76 (-2.11, -1.40) |
| Georgia | 17.37 (11.64, 24.06) | 3.40 (2.28, 4.72) | 33.65 (24.27, 44.53) | 6.01 (4.34, 7.95) | 2.06 (1.49, 2.63) |
| Thailand | 83.93 (49.10, 127.83) | 3.41 (2.00, 5.19) | 564.03 (348.03, 888.22) | 5.88 (3.63, 9.24) | 2.43 (2.19, 2.66) |
| Oman | 0.44 (0.24, 0.77) | 0.97 (0.52, 1.69) | 1.50 (0.92, 2.50) | 1.37 (0.84, 2.27) | 1.14 (0.92, 1.37) |
| Bulgaria | 39.36 (30.09, 49.38) | 3.71 (2.85, 4.64) | 49.96 (37.04, 65.48) | 3.41 (2.52, 4.47) | 0.22 (-0.10, 0.55) |
| China | 1653.44 (1028.50, 2959.37) | 2.69 (1.68, 4.82) | 11551.75 (6804.24, 15882.37) | 6.06 (3.58, 8.33) | 3.55 (2.93, 4.19) |
| Denmark | 16.89 (13.79, 20.50) | 2.09 (1.70, 2.53) | 24.38 (18.58, 30.99) | 2.01 (1.53, 2.56) | -0.82 (-1.14, -0.50) |
| Uruguay | 3.45 (2.63, 4.44) | 0.94 (0.72, 1.21) | 4.23 (3.25, 5.46) | 0.78 (0.60, 1.01) | -0.73 (-1.10, -0.36) |
| Malaysia | 14.26 (8.03, 25.97) | 2.12 (1.19, 3.85) | 63.86 (30.39, 122.67) | 2.80 (1.33, 5.39) | 0.86 (0.73, 0.99) |
| Panama | 2.12 (1.83, 2.43) | 1.74 (1.51, 2.00) | 12.48 (9.46, 15.49) | 3.19 (2.42, 3.95) | 1.71 (1.39, 2.02) |
| Norway | 11.91 (10.56, 13.25) | 1.68 (1.49, 1.87) | 21.72 (18.37, 25.03) | 2.17 (1.84, 2.49) | 0.12 (-0.27, 0.51) |
| Poland | 213.79 (199.58, 226.89) | 5.58 (5.18, 5.93) | 196.53 (171.89, 221.61) | 2.74 (2.40, 3.09) | -2.63 (-2.90, -2.36) |
| Micronesia (Federated States of) | 0.07 (0.03, 0.17) | 1.91 (0.82, 4.27) | 0.11 (0.03, 0.25) | 2.42 (0.77, 5.62) | 0.72 (0.60, 0.83) |
| Ireland | 15.50 (12.55, 19.02) | 3.91 (3.15, 4.80) | 19.87 (14.81, 25.56) | 2.60 (1.94, 3.35) | -1.14 (-1.52, -0.77) |
| Estonia | 4.70 (3.81, 5.74) | 2.60 (2.11, 3.18) | 4.45 (3.35, 5.70) | 1.64 (1.23, 2.10) | -2.37 (-2.82, -1.92) |
| Croatia | 24.87 (20.31, 30.18) | 5.15 (4.21, 6.26) | 47.48 (37.02, 59.82) | 5.26 (4.09, 6.63) | -0.37 (-0.78, 0.04) |
| Israel | 12.91 (10.00, 16.45) | 2.85 (2.21, 3.63) | 24.90 (18.69, 31.81) | 2.04 (1.54, 2.61) | -1.32 (-1.50, -1.14) |
| Viet Nam | 119.38 (64.42, 182.51) | 3.64 (1.97, 5.55) | 346.92 (191.10, 536.93) | 4.63 (2.57, 7.19) | 0.83 (0.72, 0.95) |
| United States of America | 601.43 (539.37, 651.28) | 1.92 (1.72, 2.08) | 1302.47 (1130.62, 1437.63) | 2.26 (1.97, 2.49) | 0.30 (0.10, 0.50) |
| Brazil | 347.88 (316.59, 374.32) | 5.19 (4.69, 5.61) | 905.35 (801.86, 988.21) | 4.21 (3.72, 4.60) | -0.42 (-0.60, -0.25) |
| Belarus | 41.57 (32.55, 51.47) | 3.79 (2.96, 4.69) | 36.60 (26.54, 48.98) | 2.47 (1.80, 3.30) | -1.47 (-1.63, -1.31) |
| Republic of Korea | 34.83 (22.17, 52.75) | 1.73 (1.10, 2.64) | 112.33 (67.82, 166.47) | 1.34 (0.81, 1.98) | -1.13 (-1.42, -0.84) |
| Canada | 79.20 (66.50, 92.72) | 2.61 (2.19, 3.06) | 143.21 (111.01, 177.21) | 1.98 (1.54, 2.45) | -0.74 (-0.88, -0.60) |
| Iceland | 0.86 (0.68, 1.06) | 3.16 (2.51, 3.88) | 1.95 (1.46, 2.49) | 3.40 (2.56, 4.34) | -0.55 (-0.94, -0.17) |
| Romania | 156.60 (114.43, 206.17) | 6.68 (4.93, 8.73) | 182.34 (140.64, 232.97) | 4.82 (3.72, 6.16) | -1.13 (-1.23, -1.02) |
| Qatar | 0.13 (0.08, 0.22) | 3.13 (1.90, 5.30) | 1.18 (0.70, 2.00) | 3.76 (2.23, 6.40) | 0.84 (0.65, 1.04) |
| Portugal | 64.28 (54.20, 76.01) | 5.02 (4.21, 5.96) | 95.26 (72.72, 120.46) | 3.66 (2.80, 4.62) | -1.55 (-1.90, -1.19) |
| Montenegro | 1.69 (0.94, 2.66) | 3.38 (1.89, 5.33) | 2.99 (2.05, 4.44) | 3.33 (2.27, 4.94) | -0.09 (-0.29, 0.10) |
| Sri Lanka | 13.74 (7.49, 20.60) | 1.73 (0.95, 2.60) | 65.66 (32.76, 126.44) | 2.72 (1.36, 5.23) | 2.35 (1.93, 2.77) |
| Dominican Republic | 11.76 (5.74, 18.88) | 4.26 (2.08, 6.83) | 41.26 (21.44, 65.87) | 4.91 (2.55, 7.84) | 0.72 (0.56, 0.89) |
| Venezuela (Bolivarian Republic of) | 28.38 (24.30, 32.51) | 3.88 (3.31, 4.45) | 102.94 (76.79, 133.61) | 4.14 (3.10, 5.36) | 0.30 (0.09, 0.51) |
| Democratic People's Republic of Korea | 41.51 (23.81, 73.51) | 3.76 (2.18, 6.64) | 119.65 (58.29, 237.69) | 4.45 (2.17, 8.83) | 0.74 (0.58, 0.90) |
| Yemen | 7.59 (3.74, 13.74) | 2.27 (1.10, 4.16) | 23.34 (12.12, 42.92) | 2.43 (1.26, 4.47) | 0.27 (0.21, 0.33) |
| Suriname | 0.65 (0.36, 1.00) | 3.41 (1.89, 5.19) | 1.51 (0.84, 2.47) | 2.96 (1.64, 4.82) | -0.14 (-0.23, -0.05) |
| Italy | 456.28 (405.55, 499.75) | 5.38 (4.77, 5.91) | 436.90 (360.36, 501.60) | 2.87 (2.40, 3.27) | -1.71 (-2.12, -1.31) |
| Indonesia | 173.50 (110.84, 237.71) | 2.60 (1.67, 3.56) | 786.49 (355.01, 1254.56) | 4.66 (2.14, 7.39) | 1.99 (1.84, 2.13) |
| Egypt | 106.97 (59.16, 168.32) | 6.68 (3.63, 10.52) | 288.11 (175.68, 430.43) | 7.44 (4.41, 11.36) | 0.44 (0.28, 0.59) |
| Papua New Guinea | 1.22 (0.34, 3.43) | 1.10 (0.30, 3.07) | 3.88 (0.85, 10.77) | 1.29 (0.28, 3.60) | 0.50 (0.33, 0.67) |
| Latvia | 10.31 (8.33, 12.67) | 3.25 (2.62, 3.99) | 10.24 (7.72, 13.21) | 2.61 (1.97, 3.37) | -1.31 (-1.72, -0.89) |
| Serbia | 48.33 (28.04, 83.89) | 5.87 (3.39, 10.10) | 85.74 (53.10, 129.60) | 5.22 (3.23, 7.91) | -0.66 (-0.78, -0.54) |
| Cyprus | 2.70 (1.42, 4.17) | 4.01 (2.08, 6.30) | 6.55 (3.84, 9.87) | 3.46 (2.01, 5.27) | -0.33 (-0.50, -0.15) |
| Bosnia and Herzegovina | 8.50 (4.39, 16.00) | 2.96 (1.54, 5.59) | 16.72 (10.11, 27.77) | 2.78 (1.68, 4.61) | -0.27 (-0.34, -0.20) |
| Pakistan | 125.65 (84.60, 181.22) | 2.84 (1.91, 4.10) | 277.44 (181.04, 406.37) | 3.39 (2.22, 4.96) | 0.46 (0.35, 0.57) |
| Tonga | 0.06 (0.02, 0.14) | 1.40 (0.55, 3.24) | 0.13 (0.04, 0.30) | 1.95 (0.59, 4.54) | 1.07 (0.89, 1.25) |
| Lesotho | 2.06 (1.18, 3.38) | 3.07 (1.75, 5.03) | 3.61 (1.98, 5.90) | 4.54 (2.47, 7.49) | 1.71 (1.44, 1.99) |
| Kyrgyzstan | 8.96 (6.64, 11.81) | 3.94 (2.92, 5.20) | 21.39 (15.22, 28.95) | 6.36 (4.56, 8.56) | 1.83 (1.32, 2.35) |
| Luxembourg | 1.65 (1.43, 1.88) | 3.30 (2.86, 3.77) | 2.72 (2.27, 3.21) | 2.71 (2.27, 3.19) | -0.63 (-0.81, -0.46) |
| Cambodia | 11.09 (6.40, 19.43) | 3.45 (1.99, 6.02) | 43.52 (18.94, 85.66) | 4.76 (2.09, 9.33) | 1.16 (1.09, 1.23) |
| Slovakia | 19.40 (13.33, 29.75) | 3.56 (2.45, 5.45) | 30.01 (18.23, 47.07) | 3.24 (1.97, 5.08) | -0.46 (-0.52, -0.39) |
| Samoa | 0.28 (0.12, 0.51) | 4.47 (1.89, 8.05) | 0.52 (0.25, 0.84) | 4.76 (2.28, 7.78) | 0.29 (0.15, 0.43) |
| Tunisia | 9.94 (5.53, 15.31) | 2.66 (1.49, 4.11) | 32.58 (17.36, 52.32) | 2.99 (1.60, 4.80) | 0.49 (0.45, 0.54) |
| Spain | 233.56 (199.92, 269.98) | 4.52 (3.86, 5.24) | 287.95 (217.52, 365.50) | 2.90 (2.20, 3.66) | -1.19 (-1.46, -0.91) |
| Saint Lucia | 0.23 (0.20, 0.27) | 3.11 (2.66, 3.60) | 0.44 (0.35, 0.54) | 2.16 (1.73, 2.65) | -1.43 (-1.64, -1.23) |
| Timor-Leste | 0.44 (0.25, 0.78) | 2.86 (1.63, 5.08) | 2.76 (1.19, 5.55) | 3.79 (1.63, 7.61) | 1.09 (1.02, 1.16) |
| Dominica | 0.22 (0.12, 0.34) | 3.94 (2.18, 6.09) | 0.34 (0.19, 0.52) | 4.84 (2.73, 7.51) | 0.86 (0.60, 1.11) |
| Lao People's Democratic Republic | 5.41 (2.99, 9.82) | 3.48 (1.93, 6.30) | 15.04 (6.47, 31.09) | 4.67 (2.04, 9.58) | 1.02 (0.90, 1.13) |
| Lithuania | 11.12 (9.03, 13.54) | 2.81 (2.28, 3.41) | 9.31 (7.12, 11.87) | 1.68 (1.29, 2.15) | -2.40 (-2.84, -1.95) |
| Burkina Faso | 6.89 (3.73, 13.19) | 2.10 (1.13, 4.00) | 12.08 (6.27, 20.86) | 1.79 (0.93, 3.08) | -0.62 (-0.73, -0.51) |
| Sweden | 38.04 (31.24, 45.67) | 2.42 (1.98, 2.90) | 31.77 (24.39, 40.12) | 1.40 (1.08, 1.76) | -1.42 (-1.68, -1.15) |
| Belgium | 57.30 (45.80, 70.75) | 3.90 (3.12, 4.83) | 72.95 (55.44, 92.42) | 3.07 (2.35, 3.88) | -0.89 (-1.27, -0.51) |
| United Arab Emirates | 0.90 (0.50, 1.63) | 4.32 (2.40, 7.93) | 4.62 (2.34, 7.74) | 4.27 (2.11, 7.25) | 2.05 (1.44, 2.67) |
| Albania | 10.36 (6.74, 14.70) | 6.67 (4.33, 9.51) | 23.42 (10.77, 40.81) | 5.75 (2.66, 9.99) | -0.48 (-0.58, -0.39) |
| Fiji | 0.54 (0.26, 0.89) | 2.37 (1.15, 3.93) | 1.58 (0.87, 2.48) | 2.92 (1.59, 4.58) | 0.57 (0.24, 0.89) |
| United Kingdom | 154.42 (141.64, 166.45) | 1.72 (1.57, 1.85) | 283.64 (247.95, 313.14) | 2.15 (1.89, 2.36) | 0.90 (0.65, 1.15) |
| Cuba | 43.18 (33.93, 54.23) | 4.68 (3.67, 5.88) | 128.92 (99.38, 163.47) | 7.19 (5.54, 9.11) | 0.87 (0.61, 1.14) |
| Marshall Islands | 0.02 (0.01, 0.04) | 1.40 (0.46, 3.64) | 0.04 (0.01, 0.10) | 1.85 (0.52, 4.56) | 0.75 (0.55, 0.95) |
| Belize | 0.22 (0.16, 0.27) | 2.74 (2.04, 3.36) | 0.55 (0.46, 0.64) | 2.50 (2.08, 2.94) | -0.29 (-0.61, 0.03) |
| Slovenia | 5.75 (4.62, 7.08) | 2.65 (2.13, 3.26) | 10.05 (7.33, 13.33) | 2.24 (1.64, 2.98) | -0.66 (-1.07, -0.25) |
| Republic of Moldova | 20.20 (16.78, 23.79) | 5.60 (4.65, 6.58) | 13.27 (11.24, 15.52) | 2.38 (2.01, 2.78) | -3.31 (-3.78, -2.83) |
| Ukraine | 114.38 (88.33, 147.13) | 1.81 (1.39, 2.32) | 121.47 (86.23, 165.74) | 1.63 (1.15, 2.22) | -0.46 (-0.77, -0.14) |
| Austria | 24.67 (20.00, 30.03) | 2.13 (1.72, 2.60) | 42.96 (32.94, 54.57) | 2.37 (1.83, 3.00) | -0.12 (-0.44, 0.21) |
| Mongolia | 4.87 (2.76, 7.80) | 5.63 (3.20, 8.99) | 6.59 (4.05, 9.61) | 4.52 (2.76, 6.61) | -0.91 (-1.07, -0.75) |
| Netherlands | 43.98 (36.02, 53.06) | 2.30 (1.88, 2.78) | 77.68 (59.42, 97.88) | 2.19 (1.68, 2.76) | -0.49 (-0.70, -0.28) |
| Antigua and Barbuda | 0.13 (0.10, 0.15) | 2.36 (1.94, 2.81) | 0.22 (0.19, 0.25) | 2.56 (2.18, 2.98) | 0.49 (0.21, 0.77) |
| Malta | 1.57 (1.22, 1.97) | 4.08 (3.18, 5.12) | 3.12 (2.35, 4.03) | 3.06 (2.30, 3.95) | -1.09 (-1.33, -0.85) |
| Vanuatu | 0.06 (0.02, 0.14) | 1.38 (0.46, 3.49) | 0.21 (0.06, 0.52) | 1.84 (0.53, 4.55) | 0.89 (0.75, 1.03) |
| Singapore | 3.06 (2.41, 3.79) | 1.82 (1.43, 2.25) | 8.65 (6.49, 11.18) | 1.16 (0.87, 1.49) | -1.41 (-1.81, -1.02) |
| Turkmenistan | 7.76 (4.90, 11.14) | 5.60 (3.52, 8.06) | 12.80 (7.94, 19.66) | 4.53 (2.81, 6.97) | -0.97 (-1.09, -0.84) |
| Australia | 46.49 (38.33, 55.14) | 2.55 (2.10, 3.04) | 74.88 (57.23, 95.39) | 1.64 (1.26, 2.09) | -1.42 (-1.64, -1.21) |
| New Zealand | 10.00 (8.00, 12.35) | 2.66 (2.12, 3.29) | 15.07 (11.51, 19.17) | 1.84 (1.41, 2.34) | -1.45 (-1.77, -1.12) |
| Czechia | 43.02 (36.04, 51.08) | 3.33 (2.79, 3.96) | 53.25 (41.44, 67.78) | 2.41 (1.87, 3.06) | -1.11 (-1.47, -0.74) |
| Tajikistan | 11.51 (6.58, 18.68) | 5.69 (3.26, 9.24) | 14.74 (8.63, 22.64) | 3.98 (2.35, 6.13) | -1.57 (-1.90, -1.25) |
| Russian Federation | 1060.29 (998.34, 1116.10) | 7.17 (6.72, 7.56) | 430.70 (383.06, 478.72) | 1.88 (1.68, 2.10) | -5.32 (-5.68, -4.96) |
| Botswana | 1.30 (0.77, 2.17) | 3.33 (1.97, 5.54) | 3.49 (2.03, 5.65) | 3.56 (2.06, 5.79) | 0.34 (0.18, 0.50) |
| Namibia | 1.89 (1.00, 2.84) | 4.06 (2.13, 6.16) | 3.89 (1.90, 6.31) | 4.04 (1.98, 6.58) | -0.31 (-0.61, -0.01) |
| Kiribati | 0.03 (0.02, 0.05) | 1.33 (0.75, 2.19) | 0.07 (0.04, 0.13) | 1.65 (0.90, 3.02) | 0.82 (0.58, 1.07) |
| Bolivia (Plurinational State of) | 10.70 (6.16, 16.64) | 4.61 (2.65, 7.16) | 35.47 (18.69, 59.00) | 5.01 (2.66, 8.30) | 0.25 (0.20, 0.30) |
| Maldives | 0.17 (0.10, 0.30) | 3.15 (1.88, 5.55) | 0.62 (0.35, 1.13) | 2.86 (1.62, 5.17) | -0.41 (-0.55, -0.28) |
| Grenada | 0.32 (0.22, 0.40) | 4.51 (3.08, 5.67) | 0.29 (0.24, 0.34) | 3.10 (2.53, 3.68) | -0.90 (-1.42, -0.38) |
| Finland | 15.34 (12.19, 18.85) | 2.25 (1.78, 2.76) | 13.64 (10.27, 17.47) | 1.02 (0.77, 1.30) | -2.63 (-2.80, -2.46) |
| Afghanistan | 19.81 (11.01, 34.06) | 3.82 (2.09, 6.57) | 26.28 (14.97, 43.72) | 4.27 (2.44, 7.09) | 0.49 (0.35, 0.62) |
| Switzerland | 20.86 (16.79, 25.48) | 2.05 (1.65, 2.51) | 29.57 (21.47, 38.78) | 1.61 (1.17, 2.10) | -0.97 (-1.19, -0.75) |
| Bangladesh | 91.34 (53.27, 160.12) | 2.62 (1.53, 4.60) | 279.99 (157.28, 475.28) | 2.48 (1.40, 4.21) | -0.32 (-0.39, -0.25) |
| Solomon Islands | 0.11 (0.03, 0.31) | 1.35 (0.40, 3.63) | 0.37 (0.10, 0.96) | 1.68 (0.47, 4.36) | 0.67 (0.54, 0.79) |
