# Supplementary material for: Global, regional, and national burden of malignant neoplasm of bone and articular cartilage in adults aged 65 years and older, 1990–2021: a systematic analysis based on the global burden of disease study 2021
Source: Aging Clin Exp Res. 2025 Jan 8;37(1):21. doi: 10.1007/s40520-024-02926-0 (PMC11711276; doi:10.1007/s40520-024-02926-0)
Supplement: Supplementary file 7 — Supplementary file7 (DOCX 46 KB) [file 40520_2024_2926_MOESM7_ESM.docx]

Table S4 MNBAC death in people aged ≥65 years in 1990 and 2021 for both sexes and EAPC in age-standardized rates by location

| location | Number in 1990 (95% CI) | Rate in 1990 (95% CI) | Number in 2021 (95% CI) | Rate in 2021 (95% CI) | EAPC in age-standardized rates between 1990 and 2021 (95%CI) |
| --- | --- | --- | --- | --- | --- |
| Global | 11115.53 (10003.34, 13025.66) | 3.56 (3.19, 4.16) | 27588.16 (21346.95, 32119.10) | 3.66 (2.83, 4.26) | 0.22 (0.11, 0.34) |
| High SDI | 2442.77 (2245.29, 2577.79) | 2.37 (2.17, 2.50) | 3751.09 (3246.38, 4122.06) | 1.74 (1.52, 1.91) | -1.12 (-1.26, -0.98) |
| High-middle SDI | 3992.39 (3605.00, 4540.44) | 5.02 (4.51, 5.70) | 7486.03 (5128.62, 9683.52) | 4.16 (2.86, 5.38) | -0.49 (-0.63, -0.35) |
| Middle SDI | 2638.72 (2130.89, 3706.21) | 3.69 (2.99, 5.13) | 10843.01 (8110.84, 13042.65) | 4.94 (3.70, 5.93) | 1.33 (1.03, 1.63) |
| Low-middle SDI | 1441.23 (1114.75, 1737.58) | 3.44 (2.67, 4.16) | 4249.75 (3428.94, 5066.88) | 3.89 (3.14, 4.64) | 0.38 (0.35, 0.41) |
| Low SDI | 581.90 (448.30, 729.73) | 3.76 (2.88, 4.73) | 1231.26 (964.63, 1628.89) | 3.53 (2.76, 4.66) | -0.29 (-0.37, -0.21) |
| Eastern Sub-Saharan Africa | 292.26 (226.90, 384.43) | 5.66 (4.37, 7.46) | 560.83 (408.65, 828.75) | 5.02 (3.66, 7.39) | -0.54 (-0.60, -0.48) |
| Western Sub-Saharan Africa | 168.06 (118.39, 222.46) | 2.63 (1.86, 3.47) | 321.81 (242.05, 422.89) | 2.49 (1.88, 3.28) | -0.22 (-0.26, -0.17) |
| Central Sub-Saharan Africa | 51.45 (31.86, 81.31) | 3.82 (2.33, 6.10) | 97.59 (54.75, 150.11) | 3.05 (1.70, 4.73) | -0.85 (-0.97, -0.73) |
| North Africa and Middle East | 459.46 (346.22, 612.20) | 4.06 (3.05, 5.43) | 1264.56 (986.23, 1682.64) | 3.99 (3.11, 5.33) | 0.03 (-0.04, 0.11) |
| Oceania | 3.10 (1.75, 6.15) | 1.75 (1.01, 3.40) | 8.79 (4.21, 17.93) | 1.92 (0.93, 3.90) | 0.35 (0.17, 0.53) |
| South Asia | 1136.73 (841.10, 1408.64) | 2.98 (2.20, 3.71) | 3706.42 (2983.13, 4775.82) | 3.25 (2.61, 4.18) | 0.14 (0.05, 0.23) |
| Southeast Asia | 691.91 (550.49, 867.75) | 3.88 (3.08, 4.88) | 2811.64 (1855.01, 3628.45) | 5.73 (3.82, 7.37) | 1.49 (1.32, 1.65) |
| Caribbean | 100.98 (85.03, 119.69) | 4.63 (3.89, 5.49) | 257.93 (209.88, 310.78) | 5.40 (4.40, 6.51) | 0.32 (0.13, 0.52) |
| Southern Sub-Saharan Africa | 62.59 (42.47, 80.43) | 3.14 (2.11, 4.06) | 125.51 (101.99, 157.33) | 3.00 (2.42, 3.75) | -0.41 (-0.72, -0.11) |
| Central Latin America | 258.06 (239.61, 274.12) | 4.23 (3.91, 4.50) | 914.56 (803.93, 1021.32) | 4.41 (3.88, 4.92) | 0.35 (0.08, 0.62) |
| Central Asia | 129.91 (98.58, 159.41) | 3.72 (2.82, 4.58) | 234.85 (196.84, 276.79) | 4.00 (3.35, 4.71) | 0.30 (0.08, 0.51) |
| Andean Latin America | 80.88 (63.29, 103.67) | 5.26 (4.11, 6.74) | 225.57 (165.17, 304.72) | 4.57 (3.34, 6.17) | -0.55 (-0.66, -0.44) |
| Tropical Latin America | 415.96 (378.54, 447.91) | 6.19 (5.58, 6.70) | 1095.26 (971.75, 1193.75) | 5.01 (4.44, 5.47) | -0.42 (-0.58, -0.25) |
| Central Europe | 741.50 (655.41, 827.19) | 5.83 (5.14, 6.51) | 722.10 (631.47, 814.22) | 3.24 (2.83, 3.65) | -2.15 (-2.34, -1.96) |
| Southern Latin America | 275.78 (225.28, 333.47) | 6.91 (5.63, 8.36) | 302.06 (253.44, 355.68) | 3.69 (3.10, 4.34) | -2.04 (-2.27, -1.82) |
| East Asia | 2003.37 (1291.86, 3482.53) | 3.25 (2.10, 5.66) | 10630.11 (6415.50, 14382.61) | 5.53 (3.35, 7.48) | 2.50 (1.82, 3.18) |
| Eastern Europe | 1445.02 (1358.45, 1520.24) | 6.22 (5.82, 6.55) | 609.81 (544.37, 671.57) | 1.83 (1.63, 2.01) | -4.85 (-5.15, -4.56) |
| Western Europe | 1886.70 (1720.46, 2007.47) | 3.37 (3.07, 3.59) | 1994.12 (1696.42, 2212.39) | 1.99 (1.72, 2.20) | -1.76 (-1.95, -1.56) |
| High-income Asia Pacific | 219.14 (194.78, 243.49) | 1.31 (1.16, 1.46) | 384.45 (312.21, 443.17) | 0.75 (0.62, 0.86) | -1.70 (-1.95, -1.45) |
| High-income North America | 640.67 (581.03, 675.13) | 1.87 (1.69, 1.97) | 1250.58 (1082.97, 1346.76) | 1.92 (1.67, 2.07) | -0.11 (-0.33, 0.12) |
| Australasia | 51.98 (44.43, 60.02) | 2.40 (2.04, 2.78) | 69.62 (54.62, 85.59) | 1.28 (1.01, 1.57) | -2.03 (-2.24, -1.83) |
| Mexico | 137.35 (129.78, 143.99) | 4.49 (4.22, 4.71) | 493.66 (434.49, 552.85) | 4.84 (4.26, 5.41) | 0.85 (0.34, 1.36) |
| Guatemala | 11.30 (9.21, 13.29) | 5.51 (4.39, 6.53) | 36.40 (30.21, 43.71) | 4.14 (3.44, 4.97) | -0.82 (-1.05, -0.60) |
| Guinea | 7.09 (4.02, 11.15) | 2.66 (1.51, 4.18) | 11.05 (5.68, 18.30) | 2.69 (1.39, 4.43) | -0.01 (-0.05, 0.03) |
| Gambia | 0.59 (0.33, 0.98) | 2.40 (1.35, 3.93) | 1.88 (0.97, 3.03) | 2.68 (1.39, 4.33) | 0.17 (0.07, 0.28) |
| El Salvador | 5.55 (3.98, 7.39) | 2.29 (1.64, 3.05) | 16.90 (10.48, 25.55) | 2.94 (1.83, 4.44) | 0.82 (0.76, 0.89) |
| Costa Rica | 4.15 (3.30, 5.15) | 2.91 (2.32, 3.62) | 17.85 (13.48, 22.93) | 3.72 (2.81, 4.78) | 1.09 (0.85, 1.32) |
| Cabo Verde | 0.27 (0.15, 0.49) | 1.22 (0.68, 2.24) | 0.45 (0.24, 0.78) | 1.40 (0.76, 2.47) | 0.30 (0.18, 0.43) |
| Peru | 36.11 (22.67, 56.32) | 3.94 (2.47, 6.13) | 111.72 (68.04, 176.23) | 3.89 (2.37, 6.13) | -0.32 (-0.51, -0.13) |
| Paraguay | 11.35 (6.80, 16.75) | 6.43 (3.86, 9.50) | 33.70 (19.63, 52.22) | 7.20 (4.21, 11.16) | 0.48 (0.27, 0.68) |
| Guinea-Bissau | 0.73 (0.40, 1.33) | 2.65 (1.44, 4.78) | 0.99 (0.54, 1.68) | 2.29 (1.24, 3.87) | -0.52 (-0.56, -0.49) |
| Colombia | 57.60 (48.35, 68.22) | 4.47 (3.74, 5.31) | 184.22 (140.41, 233.91) | 3.80 (2.90, 4.81) | -1.08 (-1.29, -0.88) |
| Sao Tome and Principe | 0.10 (0.06, 0.18) | 1.94 (1.10, 3.50) | 0.15 (0.09, 0.25) | 2.09 (1.17, 3.39) | 0.13 (0.06, 0.20) |
| Saint Vincent and the Grenadines | 0.14 (0.12, 0.16) | 2.21 (1.91, 2.52) | 0.90 (0.76, 1.06) | 7.50 (6.31, 8.84) | 3.41 (2.14, 4.70) |
| Trinidad and Tobago | 2.98 (2.61, 3.43) | 4.26 (3.71, 4.91) | 5.15 (3.89, 6.56) | 2.99 (2.26, 3.80) | -1.13 (-1.31, -0.94) |
| Jamaica | 9.86 (7.37, 12.68) | 5.78 (4.32, 7.44) | 15.03 (10.63, 20.50) | 5.52 (3.91, 7.55) | 0.00 (-0.28, 0.29) |
| Turkey | 94.39 (58.05, 149.51) | 3.98 (2.45, 6.30) | 261.94 (155.93, 393.57) | 3.43 (2.05, 5.15) | -0.36 (-0.66, -0.05) |
| Bermuda | 0.17 (0.12, 0.22) | 3.19 (2.38, 4.15) | 0.29 (0.21, 0.39) | 2.13 (1.57, 2.88) | -1.27 (-1.53, -1.00) |
| Saudi Arabia | 13.73 (7.85, 21.93) | 3.67 (2.10, 5.86) | 33.30 (19.18, 52.62) | 3.86 (2.25, 6.10) | 0.09 (-0.01, 0.19) |
| Burundi | 8.02 (4.20, 12.73) | 4.57 (2.40, 7.23) | 12.30 (5.49, 20.97) | 4.05 (1.82, 6.88) | -0.58 (-0.66, -0.51) |
| Morocco | 24.27 (13.84, 40.00) | 2.22 (1.26, 3.65) | 65.55 (37.83, 106.45) | 2.53 (1.46, 4.10) | 0.56 (0.44, 0.69) |
| Palestine | 3.65 (1.95, 5.76) | 5.70 (3.04, 9.01) | 10.02 (6.37, 15.50) | 6.31 (4.01, 9.78) | 0.62 (0.46, 0.78) |
| Bahrain | 0.56 (0.35, 0.95) | 6.26 (3.94, 10.57) | 2.47 (1.49, 4.27) | 6.06 (3.65, 10.50) | -0.21 (-0.33, -0.10) |
| Algeria | 35.93 (21.68, 55.58) | 4.45 (2.71, 6.98) | 105.49 (54.67, 169.69) | 4.28 (2.21, 6.90) | 0.02 (-0.08, 0.13) |
| Central African Republic | 2.42 (1.29, 4.26) | 3.69 (1.96, 6.56) | 3.38 (1.61, 5.44) | 3.00 (1.40, 4.90) | -0.74 (-0.79, -0.69) |
| Syrian Arab Republic | 3.96 (2.04, 7.69) | 1.13 (0.58, 2.19) | 10.78 (5.59, 20.29) | 1.23 (0.64, 2.37) | 0.15 (0.07, 0.22) |
| Equatorial Guinea | 0.44 (0.22, 0.79) | 3.55 (1.78, 6.47) | 1.09 (0.54, 1.84) | 3.50 (1.74, 5.85) | 0.11 (0.03, 0.20) |
| Iran (Islamic Republic of) | 44.68 (34.91, 57.46) | 2.79 (2.18, 3.58) | 148.99 (114.97, 197.48) | 2.61 (2.01, 3.47) | -0.14 (-0.20, -0.08) |
| Jordan | 3.45 (1.98, 5.32) | 4.29 (2.44, 6.65) | 20.99 (12.59, 33.93) | 4.55 (2.72, 7.35) | 0.25 (0.09, 0.41) |
| Nepal | 16.93 (10.06, 27.58) | 2.78 (1.65, 4.53) | 56.18 (32.13, 89.71) | 3.14 (1.79, 5.00) | 0.44 (0.29, 0.60) |
| Congo | 2.96 (1.66, 4.74) | 4.21 (2.35, 6.84) | 5.83 (3.04, 9.34) | 3.70 (1.94, 5.93) | -0.59 (-0.68, -0.50) |
| Democratic Republic of the Congo | 36.24 (19.76, 62.35) | 3.88 (2.08, 6.82) | 64.02 (29.95, 106.31) | 2.97 (1.38, 4.97) | -0.99 (-1.14, -0.85) |
| Libya | 4.05 (2.23, 6.75) | 2.97 (1.64, 4.97) | 11.59 (6.53, 19.03) | 3.41 (1.92, 5.59) | 0.80 (0.66, 0.93) |
| Comoros | 0.68 (0.34, 1.08) | 4.98 (2.52, 7.89) | 1.73 (0.77, 3.15) | 4.89 (2.18, 8.85) | -0.17 (-0.23, -0.12) |
| Lebanon | 6.33 (3.62, 9.86) | 4.12 (2.35, 6.42) | 23.64 (13.18, 36.24) | 4.10 (2.28, 6.28) | 0.14 (-0.10, 0.39) |
| Kuwait | 0.93 (0.72, 1.18) | 2.89 (2.22, 3.65) | 5.02 (3.54, 6.80) | 3.06 (2.16, 4.15) | 0.83 (-0.53, 2.21) |
| Gabon | 1.75 (0.93, 2.92) | 3.98 (2.10, 6.69) | 2.60 (1.32, 4.31) | 3.77 (1.92, 6.25) | -0.38 (-0.49, -0.26) |
| Sudan | 20.98 (11.34, 36.14) | 2.99 (1.60, 5.19) | 43.08 (25.49, 71.72) | 3.28 (1.94, 5.47) | 0.34 (0.27, 0.40) |
| Djibouti | 0.33 (0.16, 0.56) | 4.45 (2.17, 7.45) | 1.85 (0.84, 3.33) | 5.04 (2.28, 8.88) | 0.39 (0.32, 0.45) |
| Eritrea | 2.45 (1.19, 3.78) | 4.59 (2.25, 7.15) | 7.39 (3.28, 12.72) | 4.74 (2.12, 8.18) | -0.00 (-0.06, 0.05) |
| Kenya | 30.05 (22.36, 39.10) | 5.09 (3.77, 6.61) | 86.10 (63.03, 120.83) | 5.61 (4.10, 7.84) | 0.40 (0.36, 0.44) |
| Iraq | 33.46 (17.84, 52.23) | 5.46 (2.91, 8.52) | 91.71 (54.39, 140.57) | 5.91 (3.52, 8.99) | 0.36 (0.27, 0.46) |
| Malawi | 12.23 (6.14, 18.60) | 4.63 (2.33, 7.08) | 21.65 (10.39, 36.67) | 4.28 (2.05, 7.27) | -0.31 (-0.38, -0.25) |
| Angola | 7.64 (3.91, 14.16) | 3.47 (1.77, 6.50) | 20.67 (9.88, 34.02) | 3.08 (1.46, 5.11) | -0.49 (-0.59, -0.39) |
| Seychelles | 0.05 (0.01, 0.12) | 1.05 (0.29, 2.35) | 0.09 (0.02, 0.20) | 1.04 (0.25, 2.26) | 0.14 (-0.28, 0.55) |
| Madagascar | 15.84 (8.25, 24.79) | 4.42 (2.31, 6.88) | 22.87 (10.16, 39.08) | 3.61 (1.62, 6.13) | -0.58 (-0.76, -0.40) |
| Ethiopia | 99.26 (70.01, 140.26) | 7.59 (5.30, 10.82) | 180.47 (123.72, 249.25) | 5.82 (3.99, 8.05) | -1.18 (-1.31, -1.05) |
| Mozambique | 20.07 (9.95, 31.30) | 5.06 (2.51, 7.90) | 34.25 (15.72, 56.73) | 4.83 (2.21, 8.07) | -0.03 (-0.11, 0.05) |
| Rwanda | 10.17 (5.15, 15.48) | 5.33 (2.70, 8.11) | 17.99 (8.01, 32.96) | 4.32 (1.94, 7.89) | -1.03 (-1.17, -0.88) |
| Uganda | 25.51 (15.20, 38.34) | 5.46 (3.25, 8.23) | 48.64 (25.73, 78.55) | 5.04 (2.67, 8.13) | -0.59 (-0.74, -0.45) |
| Mauritius | 3.35 (2.95, 3.79) | 5.93 (5.21, 6.69) | 8.53 (7.41, 9.68) | 5.30 (4.60, 6.02) | 1.01 (-0.50, 2.55) |
| Somalia | 6.29 (3.06, 10.02) | 5.13 (2.49, 8.18) | 16.00 (6.73, 26.91) | 4.48 (1.89, 7.63) | -0.39 (-0.42, -0.36) |
| United Republic of Tanzania | 40.59 (21.12, 62.21) | 5.16 (2.70, 7.92) | 77.89 (35.99, 136.54) | 4.40 (2.05, 7.69) | -0.65 (-0.71, -0.59) |
| Eswatini | 0.98 (0.57, 1.56) | 5.31 (3.07, 8.43) | 2.05 (1.05, 3.51) | 5.66 (2.88, 9.76) | 0.31 (-0.04, 0.65) |
| Zambia | 9.72 (4.90, 14.94) | 5.15 (2.62, 7.93) | 19.22 (8.63, 33.99) | 4.43 (2.02, 7.80) | -0.67 (-0.75, -0.59) |
| Chad | 4.86 (2.58, 9.04) | 2.06 (1.09, 3.84) | 8.74 (4.17, 15.94) | 2.23 (1.07, 4.04) | 0.21 (0.18, 0.25) |
| Benin | 3.86 (2.27, 6.83) | 2.42 (1.43, 4.28) | 7.75 (4.21, 13.18) | 2.18 (1.18, 3.69) | -0.45 (-0.52, -0.37) |
| Coted'Ivoire | 4.77 (2.85, 8.40) | 2.12 (1.27, 3.69) | 13.14 (7.62, 22.73) | 1.93 (1.12, 3.31) | -0.35 (-0.41, -0.29) |
| Ghana | 8.40 (4.56, 15.89) | 2.03 (1.11, 3.81) | 25.83 (13.85, 43.00) | 2.31 (1.24, 3.85) | 0.45 (0.42, 0.48) |
| Cameroon | 9.12 (5.53, 15.50) | 3.08 (1.86, 5.21) | 21.04 (11.28, 35.92) | 2.59 (1.39, 4.40) | -0.64 (-0.70, -0.58) |
| Liberia | 2.60 (1.51, 4.90) | 2.77 (1.60, 5.19) | 2.95 (1.62, 5.00) | 2.26 (1.24, 3.82) | -0.58 (-0.72, -0.44) |
| Niger | 4.28 (2.35, 7.87) | 2.41 (1.32, 4.41) | 11.48 (5.75, 19.93) | 2.11 (1.06, 3.65) | -0.51 (-0.56, -0.46) |
| Mali | 6.21 (3.73, 10.30) | 2.31 (1.40, 3.81) | 12.88 (7.24, 21.26) | 2.16 (1.22, 3.56) | -0.20 (-0.25, -0.15) |
| Sierra Leone | 3.45 (1.81, 6.37) | 2.07 (1.09, 3.83) | 5.05 (2.64, 8.86) | 1.88 (0.99, 3.28) | -0.41 (-0.47, -0.35) |
| Senegal | 5.94 (3.36, 10.55) | 2.45 (1.39, 4.36) | 12.96 (6.76, 21.98) | 2.31 (1.21, 3.91) | -0.20 (-0.28, -0.13) |
| Cook Islands | 0.01 (0.01, 0.02) | 1.07 (0.60, 1.95) | 0.02 (0.01, 0.04) | 0.91 (0.48, 1.80) | -0.68 (-0.80, -0.56) |
| Greenland | 0.04 (0.02, 0.07) | 2.05 (1.01, 3.56) | 0.05 (0.03, 0.08) | 0.99 (0.55, 1.60) | -2.12 (-2.53, -1.72) |
| Guam | 0.06 (0.04, 0.10) | 1.32 (0.80, 2.12) | 0.14 (0.09, 0.21) | 0.75 (0.46, 1.12) | -1.38 (-1.61, -1.16) |
| Niue | 0.00 (0.00, 0.01) | 2.13 (0.89, 4.85) | 0.00 (0.00, 0.01) | 2.62 (0.88, 5.94) | 0.64 (0.53, 0.76) |
| Palau | 0.00 (0.00, 0.01) | 0.39 (0.15, 0.92) | 0.01 (0.00, 0.01) | 0.40 (0.16, 0.94) | 0.36 (0.20, 0.52) |
| Nigeria | 93.83 (64.35, 131.95) | 2.85 (1.96, 3.97) | 162.42 (117.67, 227.87) | 2.77 (2.01, 3.86) | -0.11 (-0.21, -0.01) |
| Northern Mariana Islands | 0.01 (0.01, 0.02) | 1.80 (0.96, 3.01) | 0.06 (0.04, 0.11) | 1.84 (1.12, 3.06) | 0.59 (0.12, 1.07) |
| Mauritania | 2.20 (1.30, 3.81) | 2.70 (1.60, 4.66) | 4.17 (2.17, 7.17) | 2.59 (1.35, 4.43) | -0.35 (-0.40, -0.29) |
| Togo | 1.83 (1.05, 3.30) | 2.23 (1.27, 4.01) | 4.84 (2.63, 8.45) | 2.05 (1.12, 3.55) | -0.44 (-0.50, -0.38) |
| Saint Kitts and Nevis | 0.23 (0.20, 0.27) | 6.13 (5.26, 7.09) | 0.21 (0.17, 0.25) | 4.46 (3.64, 5.32) | -0.48 (-0.84, -0.13) |
| American Samoa | 0.05 (0.03, 0.08) | 3.42 (1.82, 5.45) | 0.15 (0.09, 0.24) | 4.17 (2.52, 6.86) | 1.04 (0.77, 1.32) |
| Puerto Rico | 4.59 (3.56, 5.81) | 1.39 (1.08, 1.77) | 16.32 (12.16, 21.23) | 2.13 (1.59, 2.78) | 1.54 (0.22, 2.88) |
| Monaco | 0.03 (0.01, 0.06) | 0.38 (0.12, 0.82) | 0.04 (0.01, 0.07) | 0.34 (0.13, 0.68) | -0.60 (-0.74, -0.46) |
| Nauru | 0.01 (0.00, 0.01) | 2.36 (0.97, 4.92) | 0.01 (0.00, 0.02) | 2.99 (0.98, 6.51) | 0.66 (0.55, 0.77) |
| Tokelau | 0.00 (0.00, 0.01) | 1.97 (0.72, 4.85) | 0.00 (0.00, 0.01) | 2.36 (0.73, 5.49) | 0.61 (0.51, 0.72) |
| San Marino | 0.12 (0.06, 0.19) | 3.52 (1.89, 5.53) | 0.13 (0.06, 0.22) | 1.57 (0.76, 2.69) | -1.94 (-2.26, -1.61) |
| South Sudan | 10.83 (5.11, 17.56) | 5.27 (2.50, 8.54) | 11.96 (5.00, 21.96) | 5.22 (2.19, 9.52) | -0.10 (-0.13, -0.07) |
| Tuvalu | 0.01 (0.00, 0.02) | 1.90 (0.66, 4.78) | 0.02 (0.01, 0.05) | 2.36 (0.71, 5.77) | 0.69 (0.56, 0.82) |
| United States Virgin Islands | 0.07 (0.05, 0.12) | 1.25 (0.77, 2.07) | 0.19 (0.10, 0.36) | 1.06 (0.53, 2.01) | -0.07 (-0.26, 0.12) |
| Taiwan (Province of China) | 58.05 (49.25, 67.37) | 4.71 (3.97, 5.49) | 79.47 (61.66, 100.32) | 2.03 (1.57, 2.56) | -2.39 (-2.89, -1.88) |
| Uzbekistan | 37.51 (18.09, 56.98) | 4.34 (2.11, 6.58) | 84.33 (61.26, 111.78) | 4.74 (3.42, 6.29) | 0.53 (0.03, 1.03) |
| Honduras | 2.93 (1.74, 5.02) | 1.95 (1.14, 3.37) | 15.87 (8.18, 29.81) | 3.34 (1.72, 6.29) | 1.81 (1.62, 1.99) |
| France | 383.01 (321.06, 439.64) | 4.74 (3.97, 5.44) | 414.35 (315.28, 522.96) | 2.61 (2.00, 3.30) | -1.89 (-2.07, -1.70) |
| Myanmar | 59.27 (33.75, 105.82) | 3.58 (2.03, 6.41) | 182.86 (82.14, 367.85) | 4.99 (2.26, 9.97) | 1.14 (0.99, 1.28) |
| Bahamas | 0.36 (0.31, 0.42) | 2.95 (2.51, 3.44) | 0.83 (0.66, 1.02) | 2.73 (2.18, 3.35) | -0.22 (-0.50, 0.06) |
| South Africa | 42.79 (24.61, 58.04) | 2.78 (1.59, 3.78) | 90.58 (72.06, 110.34) | 2.65 (2.09, 3.24) | -0.51 (-0.82, -0.19) |
| Guyana | 1.33 (1.11, 1.57) | 4.73 (3.94, 5.58) | 1.98 (1.53, 2.51) | 4.14 (3.21, 5.24) | -0.09 (-0.27, 0.08) |
| Bhutan | 0.44 (0.24, 0.71) | 2.89 (1.61, 4.71) | 1.78 (0.97, 2.88) | 3.62 (1.97, 5.88) | 0.71 (0.67, 0.75) |
| Hungary | 59.66 (49.95, 70.58) | 4.44 (3.71, 5.26) | 34.39 (26.34, 44.40) | 1.71 (1.31, 2.20) | -3.32 (-3.68, -2.96) |
| Philippines | 141.31 (111.20, 204.39) | 7.02 (5.49, 10.24) | 519.28 (400.33, 730.56) | 8.72 (6.69, 12.41) | 0.91 (0.68, 1.15) |
| Argentina | 210.17 (164.34, 264.48) | 7.47 (5.83, 9.40) | 212.41 (170.95, 260.59) | 4.02 (3.24, 4.93) | -2.02 (-2.28, -1.77) |
| North Macedonia | 12.75 (9.53, 16.84) | 8.94 (6.68, 11.82) | 20.14 (12.49, 30.53) | 7.28 (4.47, 11.05) | -0.87 (-1.16, -0.59) |
| Germany | 216.78 (174.79, 263.51) | 1.78 (1.43, 2.16) | 279.83 (219.84, 343.32) | 1.39 (1.10, 1.70) | -1.06 (-1.22, -0.89) |
| Andorra | 0.02 (0.01, 0.04) | 0.40 (0.15, 0.87) | 0.04 (0.01, 0.08) | 0.26 (0.10, 0.55) | -1.33 (-1.51, -1.15) |
| Nicaragua | 3.32 (2.04, 4.96) | 2.96 (1.82, 4.43) | 13.93 (8.80, 21.95) | 3.66 (2.30, 5.78) | 0.96 (0.67, 1.25) |
| Greece | 157.25 (140.26, 173.93) | 11.28 (10.02, 12.50) | 117.84 (98.85, 135.50) | 4.40 (3.74, 5.04) | -3.95 (-4.35, -3.54) |
| Chile | 61.52 (47.48, 77.64) | 7.62 (5.88, 9.61) | 85.05 (67.24, 104.76) | 3.63 (2.87, 4.48) | -2.37 (-2.55, -2.17) |
| Barbados | 1.21 (1.03, 1.40) | 4.04 (3.43, 4.67) | 1.75 (1.36, 2.14) | 3.64 (2.83, 4.46) | 0.08 (-0.09, 0.26) |
| Azerbaijan | 8.30 (4.79, 15.19) | 2.41 (1.39, 4.41) | 16.42 (9.80, 28.72) | 2.41 (1.44, 4.22) | 0.13 (0.04, 0.22) |
| Ecuador | 32.25 (25.15, 40.76) | 8.26 (6.40, 10.47) | 72.16 (53.58, 95.15) | 5.34 (3.98, 7.02) | -1.24 (-1.38, -1.11) |
| Haiti | 9.17 (5.09, 16.35) | 4.24 (2.30, 7.61) | 18.30 (9.37, 33.55) | 4.01 (2.04, 7.43) | -0.08 (-0.15, -0.01) |
| Brunei Darussalam | 0.07 (0.04, 0.11) | 0.98 (0.61, 1.63) | 0.20 (0.12, 0.34) | 0.89 (0.52, 1.54) | 0.51 (0.25, 0.77) |
| India | 866.59 (631.02, 1089.76) | 2.90 (2.10, 3.67) | 2997.83 (2393.63, 3887.27) | 3.22 (2.56, 4.17) | 0.20 (0.09, 0.31) |
| Japan | 175.38 (160.05, 185.20) | 1.20 (1.09, 1.27) | 290.02 (238.74, 320.35) | 0.69 (0.58, 0.75) | -1.56 (-1.91, -1.22) |
| Armenia | 7.47 (5.20, 10.17) | 4.01 (2.79, 5.47) | 15.92 (11.58, 21.66) | 4.08 (2.96, 5.55) | 0.32 (0.09, 0.56) |
| Zimbabwe | 12.79 (7.14, 19.15) | 4.54 (2.52, 6.86) | 20.20 (10.42, 32.60) | 4.58 (2.35, 7.36) | 0.08 (-0.25, 0.40) |
| Kazakhstan | 18.22 (12.12, 24.46) | 1.93 (1.28, 2.60) | 17.16 (12.24, 23.06) | 1.25 (0.89, 1.69) | -1.92 (-2.30, -1.54) |
| Georgia | 19.88 (13.35, 27.48) | 3.90 (2.62, 5.41) | 37.99 (27.63, 50.42) | 6.78 (4.94, 9.00) | 2.06 (1.50, 2.63) |
| Thailand | 97.67 (57.21, 148.21) | 4.04 (2.37, 6.12) | 594.42 (364.89, 939.28) | 6.22 (3.82, 9.81) | 2.00 (1.76, 2.23) |
| Oman | 0.51 (0.28, 0.88) | 1.15 (0.62, 1.99) | 1.47 (0.90, 2.44) | 1.40 (0.85, 2.32) | 0.69 (0.49, 0.89) |
| Bulgaria | 44.60 (34.22, 55.85) | 4.38 (3.38, 5.47) | 52.70 (39.35, 68.25) | 3.61 (2.70, 4.69) | -0.19 (-0.53, 0.15) |
| China | 1897.49 (1185.11, 3386.31) | 3.20 (2.00, 5.72) | 10411.38 (6265.38, 14110.68) | 5.62 (3.39, 7.61) | 2.61 (1.90, 3.32) |
| Denmark | 16.84 (13.75, 20.25) | 2.07 (1.69, 2.49) | 19.69 (15.12, 24.71) | 1.60 (1.23, 2.01) | -1.61 (-1.95, -1.26) |
| Uruguay | 4.08 (3.12, 5.25) | 1.12 (0.85, 1.44) | 4.58 (3.52, 5.90) | 0.82 (0.63, 1.06) | -1.11 (-1.48, -0.74) |
| Malaysia | 16.56 (9.33, 29.83) | 2.47 (1.39, 4.45) | 72.36 (34.57, 139.53) | 3.24 (1.54, 6.27) | 0.86 (0.73, 0.99) |
| Panama | 2.52 (2.18, 2.89) | 2.09 (1.80, 2.40) | 14.24 (10.74, 17.57) | 3.61 (2.73, 4.46) | 1.56 (1.22, 1.90) |
| Norway | 11.46 (10.26, 12.55) | 1.61 (1.44, 1.76) | 16.52 (14.10, 18.68) | 1.62 (1.39, 1.83) | -0.70 (-1.11, -0.28) |
| Poland | 249.53 (233.09, 264.49) | 6.59 (6.12, 7.00) | 184.95 (163.73, 203.48) | 2.58 (2.29, 2.84) | -3.44 (-3.71, -3.16) |
| Micronesia (Federated States of) | 0.09 (0.04, 0.19) | 2.25 (0.96, 5.02) | 0.12 (0.04, 0.29) | 2.85 (0.90, 6.64) | 0.73 (0.61, 0.85) |
| Ireland | 15.16 (12.38, 18.46) | 3.88 (3.16, 4.73) | 14.94 (11.16, 18.95) | 1.95 (1.46, 2.47) | -2.17 (-2.58, -1.75) |
| Estonia | 5.44 (4.41, 6.65) | 3.02 (2.45, 3.70) | 3.98 (3.02, 5.10) | 1.43 (1.09, 1.84) | -3.42 (-3.89, -2.94) |
| Croatia | 26.42 (21.68, 31.65) | 5.59 (4.60, 6.69) | 39.94 (31.46, 49.82) | 4.39 (3.45, 5.48) | -1.25 (-1.65, -0.86) |
| Israel | 13.59 (10.48, 17.18) | 3.06 (2.36, 3.87) | 20.84 (15.79, 26.38) | 1.69 (1.28, 2.14) | -2.17 (-2.33, -2.01) |
| Viet Nam | 138.85 (74.58, 212.71) | 4.29 (2.31, 6.57) | 390.13 (215.85, 599.99) | 5.31 (2.96, 8.20) | 0.76 (0.63, 0.89) |
| United States of America | 571.94 (516.85, 602.53) | 1.83 (1.65, 1.93) | 1141.83 (990.72, 1224.51) | 1.98 (1.72, 2.12) | 0.02 (-0.21, 0.26) |
| Brazil | 404.61 (368.24, 435.55) | 6.19 (5.58, 6.69) | 1061.56 (940.55, 1158.14) | 4.97 (4.39, 5.42) | -0.44 (-0.61, -0.27) |
| Belarus | 48.07 (37.76, 60.00) | 4.40 (3.45, 5.49) | 34.03 (24.80, 45.32) | 2.30 (1.69, 3.06) | -2.27 (-2.37, -2.17) |
| Republic of Korea | 40.40 (25.73, 61.00) | 2.09 (1.33, 3.17) | 87.53 (53.04, 128.68) | 1.05 (0.63, 1.54) | -2.71 (-2.94, -2.47) |
| Canada | 68.68 (58.24, 79.35) | 2.29 (1.93, 2.65) | 108.68 (84.87, 133.19) | 1.49 (1.16, 1.82) | -1.26 (-1.44, -1.07) |
| Iceland | 0.76 (0.60, 0.92) | 2.77 (2.21, 3.38) | 1.44 (1.07, 1.82) | 2.47 (1.85, 3.13) | -1.14 (-1.54, -0.73) |
| Romania | 180.95 (132.56, 237.26) | 7.87 (5.82, 10.25) | 177.38 (137.32, 224.06) | 4.67 (3.61, 5.91) | -1.87 (-2.00, -1.75) |
| Qatar | 0.15 (0.09, 0.25) | 3.79 (2.30, 6.42) | 1.01 (0.60, 1.70) | 3.45 (2.06, 5.87) | -0.23 (-0.39, -0.07) |
| Portugal | 71.24 (60.34, 83.87) | 5.69 (4.79, 6.72) | 80.72 (62.13, 101.18) | 3.00 (2.32, 3.76) | -2.61 (-3.01, -2.22) |
| Montenegro | 1.72 (0.96, 2.71) | 3.47 (1.93, 5.45) | 2.67 (1.85, 3.94) | 3.05 (2.10, 4.52) | -0.62 (-0.82, -0.43) |
| Sri Lanka | 15.91 (8.68, 23.65) | 2.06 (1.13, 3.07) | 70.46 (34.91, 134.02) | 2.99 (1.49, 5.70) | 2.11 (1.66, 2.56) |
| Dominican Republic | 13.90 (6.70, 22.26) | 5.15 (2.48, 8.24) | 49.25 (25.71, 78.40) | 5.87 (3.07, 9.35) | 0.70 (0.52, 0.87) |
| Venezuela (Bolivarian Republic of) | 33.34 (28.53, 38.22) | 4.61 (3.93, 5.30) | 121.50 (91.32, 156.56) | 4.94 (3.73, 6.35) | 0.31 (0.10, 0.52) |
| Democratic People's Republic of Korea | 47.83 (27.64, 84.49) | 4.44 (2.58, 7.80) | 139.26 (67.37, 275.55) | 5.21 (2.52, 10.31) | 0.71 (0.55, 0.87) |
| Yemen | 8.69 (4.23, 15.71) | 2.69 (1.29, 4.93) | 27.11 (14.05, 50.32) | 2.90 (1.50, 5.38) | 0.30 (0.24, 0.36) |
| Suriname | 0.77 (0.42, 1.17) | 4.05 (2.22, 6.11) | 1.79 (1.00, 2.92) | 3.52 (1.96, 5.72) | -0.13 (-0.22, -0.04) |
| Italy | 424.67 (382.33, 456.70) | 5.04 (4.51, 5.43) | 343.81 (286.16, 387.43) | 2.16 (1.83, 2.43) | -2.38 (-2.86, -1.90) |
| Indonesia | 198.44 (126.89, 271.96) | 3.05 (1.96, 4.17) | 898.40 (407.87, 1433.89) | 5.49 (2.53, 8.70) | 2.01 (1.86, 2.16) |
| Egypt | 124.16 (68.63, 196.20) | 8.09 (4.38, 12.81) | 329.39 (202.70, 489.82) | 8.90 (5.29, 13.57) | 0.42 (0.26, 0.58) |
| Papua New Guinea | 1.39 (0.38, 3.91) | 1.29 (0.35, 3.62) | 4.47 (0.97, 12.51) | 1.52 (0.33, 4.29) | 0.51 (0.34, 0.68) |
| Latvia | 12.02 (9.72, 14.73) | 3.79 (3.07, 4.65) | 10.11 (7.73, 12.96) | 2.52 (1.93, 3.24) | -1.91 (-2.34, -1.48) |
| Serbia | 56.08 (32.32, 96.73) | 7.04 (4.04, 12.06) | 80.28 (49.53, 120.33) | 4.92 (3.03, 7.38) | -1.55 (-1.68, -1.42) |
| Cyprus | 2.89 (1.51, 4.45) | 4.57 (2.36, 7.19) | 5.09 (2.99, 7.75) | 2.75 (1.59, 4.23) | -1.68 (-1.81, -1.55) |
| Bosnia and Herzegovina | 9.87 (5.12, 18.69) | 3.49 (1.82, 6.63) | 16.39 (10.01, 26.66) | 2.74 (1.67, 4.46) | -1.02 (-1.14, -0.90) |
| Pakistan | 146.11 (99.12, 211.92) | 3.37 (2.28, 4.90) | 322.18 (210.71, 468.13) | 4.03 (2.64, 5.86) | 0.47 (0.36, 0.58) |
| Tonga | 0.07 (0.03, 0.16) | 1.65 (0.64, 3.82) | 0.15 (0.05, 0.35) | 2.30 (0.69, 5.29) | 1.08 (0.90, 1.26) |
| Lesotho | 2.39 (1.36, 3.92) | 3.64 (2.07, 5.97) | 4.13 (2.29, 6.73) | 5.38 (2.95, 8.83) | 1.73 (1.45, 2.01) |
| Kyrgyzstan | 10.52 (7.80, 13.91) | 4.66 (3.46, 6.16) | 24.11 (17.34, 32.70) | 7.31 (5.30, 9.84) | 1.77 (1.24, 2.30) |
| Luxembourg | 1.68 (1.47, 1.89) | 3.37 (2.95, 3.80) | 2.15 (1.79, 2.50) | 2.10 (1.76, 2.43) | -1.56 (-1.72, -1.40) |
| Cambodia | 12.65 (7.28, 22.13) | 4.04 (2.33, 7.03) | 50.00 (21.69, 98.33) | 5.61 (2.47, 10.99) | 1.19 (1.12, 1.26) |
| Slovakia | 22.14 (15.25, 33.58) | 4.12 (2.83, 6.24) | 27.31 (16.80, 42.80) | 2.99 (1.84, 4.68) | -1.20 (-1.27, -1.14) |
| Samoa | 0.33 (0.14, 0.59) | 5.27 (2.23, 9.53) | 0.60 (0.29, 0.99) | 5.63 (2.72, 9.22) | 0.30 (0.16, 0.44) |
| Tunisia | 11.57 (6.43, 18.04) | 3.20 (1.79, 5.01) | 34.46 (18.57, 55.51) | 3.24 (1.75, 5.20) | 0.12 (0.08, 0.17) |
| Spain | 228.23 (196.33, 261.45) | 4.46 (3.83, 5.12) | 223.77 (171.30, 281.86) | 2.15 (1.66, 2.70) | -2.09 (-2.42, -1.75) |
| Saint Lucia | 0.28 (0.24, 0.32) | 3.79 (3.23, 4.39) | 0.53 (0.43, 0.65) | 2.62 (2.09, 3.19) | -1.47 (-1.68, -1.27) |
| Timor-Leste | 0.51 (0.29, 0.91) | 3.36 (1.90, 5.99) | 3.18 (1.36, 6.35) | 4.47 (1.91, 8.96) | 1.10 (1.03, 1.17) |
| Dominica | 0.26 (0.15, 0.40) | 4.74 (2.61, 7.32) | 0.40 (0.23, 0.63) | 5.87 (3.29, 9.19) | 0.88 (0.62, 1.14) |
| Lao People's Democratic Republic | 6.14 (3.41, 10.98) | 4.07 (2.26, 7.29) | 17.31 (7.42, 35.54) | 5.50 (2.39, 11.20) | 1.04 (0.92, 1.16) |
| Lithuania | 12.84 (10.46, 15.59) | 3.24 (2.64, 3.93) | 9.33 (7.16, 11.80) | 1.64 (1.26, 2.08) | -2.85 (-3.29, -2.42) |
| Burkina Faso | 7.93 (4.31, 15.32) | 2.48 (1.34, 4.77) | 14.05 (7.30, 24.42) | 2.13 (1.11, 3.67) | -0.60 (-0.71, -0.49) |
| Sweden | 34.81 (28.65, 41.45) | 2.19 (1.80, 2.61) | 24.55 (19.22, 30.55) | 1.05 (0.83, 1.31) | -2.00 (-2.26, -1.74) |
| Belgium | 54.98 (44.27, 67.34) | 3.75 (3.01, 4.60) | 59.06 (44.77, 74.39) | 2.40 (1.84, 3.02) | -1.55 (-1.95, -1.15) |
| United Arab Emirates | 1.03 (0.58, 1.86) | 5.10 (2.83, 9.28) | 4.86 (2.46, 8.16) | 4.90 (2.44, 8.31) | 2.03 (1.39, 2.67) |
| Albania | 12.08 (7.84, 17.19) | 7.91 (5.12, 11.31) | 23.25 (10.89, 39.65) | 5.79 (2.73, 9.85) | -1.15 (-1.29, -1.01) |
| Fiji | 0.62 (0.30, 1.02) | 2.79 (1.34, 4.61) | 1.83 (1.02, 2.88) | 3.44 (1.90, 5.45) | 0.57 (0.24, 0.90) |
| United Kingdom | 151.92 (141.34, 158.99) | 1.69 (1.57, 1.77) | 239.73 (211.26, 256.44) | 1.78 (1.58, 1.90) | 0.27 (-0.01, 0.55) |
| Cuba | 51.44 (40.41, 64.45) | 5.63 (4.42, 7.06) | 135.04 (105.27, 170.25) | 7.43 (5.79, 9.37) | 0.40 (0.14, 0.67) |
| Marshall Islands | 0.02 (0.01, 0.05) | 1.65 (0.54, 4.25) | 0.04 (0.01, 0.11) | 2.19 (0.61, 5.41) | 0.77 (0.57, 0.97) |
| Belize | 0.26 (0.19, 0.32) | 3.24 (2.42, 3.98) | 0.65 (0.54, 0.76) | 2.98 (2.49, 3.50) | -0.25 (-0.57, 0.07) |
| Slovenia | 5.98 (4.81, 7.30) | 2.76 (2.22, 3.36) | 7.82 (5.75, 10.31) | 1.71 (1.26, 2.26) | -1.77 (-2.19, -1.34) |
| Republic of Moldova | 23.20 (19.27, 27.31) | 6.57 (5.46, 7.72) | 13.44 (11.50, 15.58) | 2.43 (2.08, 2.81) | -3.73 (-4.16, -3.30) |
| Ukraine | 127.75 (98.09, 164.63) | 2.03 (1.56, 2.62) | 126.29 (89.29, 171.32) | 1.69 (1.20, 2.29) | -0.80 (-1.15, -0.45) |
| Austria | 24.08 (19.61, 29.02) | 2.07 (1.68, 2.50) | 34.17 (26.27, 42.97) | 1.82 (1.41, 2.29) | -0.87 (-1.22, -0.51) |
| Mongolia | 5.54 (3.12, 8.81) | 6.51 (3.67, 10.31) | 7.54 (4.64, 11.03) | 5.26 (3.22, 7.71) | -0.89 (-1.06, -0.73) |
| Netherlands | 39.77 (32.87, 47.52) | 2.08 (1.72, 2.49) | 57.95 (45.04, 72.30) | 1.62 (1.26, 2.02) | -1.24 (-1.47, -1.02) |
| Antigua and Barbuda | 0.15 (0.12, 0.18) | 2.85 (2.33, 3.39) | 0.25 (0.21, 0.29) | 2.99 (2.56, 3.48) | 0.42 (0.15, 0.69) |
| Malta | 1.56 (1.22, 1.94) | 4.12 (3.23, 5.12) | 2.49 (1.87, 3.20) | 2.42 (1.82, 3.11) | -1.86 (-2.07, -1.66) |
| Vanuatu | 0.06 (0.02, 0.16) | 1.64 (0.53, 4.18) | 0.25 (0.07, 0.61) | 2.18 (0.61, 5.43) | 0.89 (0.75, 1.03) |
| Singapore | 3.29 (2.61, 4.05) | 2.00 (1.58, 2.46) | 6.70 (5.08, 8.47) | 0.91 (0.69, 1.15) | -2.55 (-2.88, -2.21) |
| Turkmenistan | 8.91 (5.59, 12.82) | 6.52 (4.06, 9.39) | 14.65 (8.98, 22.28) | 5.26 (3.22, 8.01) | -0.97 (-1.10, -0.85) |
| Australia | 42.68 (35.65, 50.33) | 2.38 (1.98, 2.82) | 57.85 (43.87, 72.66) | 1.25 (0.95, 1.57) | -2.04 (-2.25, -1.82) |
| New Zealand | 9.30 (7.51, 11.35) | 2.50 (2.01, 3.05) | 11.77 (9.09, 14.82) | 1.43 (1.10, 1.80) | -2.05 (-2.38, -1.72) |
| Czechia | 47.86 (40.35, 56.52) | 3.74 (3.15, 4.43) | 44.38 (34.37, 55.89) | 2.01 (1.55, 2.53) | -1.98 (-2.28, -1.68) |
| Tajikistan | 13.55 (7.74, 22.15) | 6.73 (3.85, 11.02) | 16.72 (9.86, 25.85) | 4.61 (2.73, 7.15) | -1.66 (-1.98, -1.35) |
| Russian Federation | 1215.71 (1148.62, 1271.95) | 8.30 (7.81, 8.70) | 412.62 (370.52, 450.91) | 1.82 (1.64, 1.99) | -5.95 (-6.29, -5.59) |
| Botswana | 1.48 (0.87, 2.49) | 3.98 (2.33, 6.64) | 4.05 (2.37, 6.58) | 4.25 (2.47, 6.96) | 0.35 (0.19, 0.51) |
| Namibia | 2.16 (1.14, 3.26) | 4.82 (2.52, 7.37) | 4.50 (2.22, 7.30) | 4.80 (2.37, 7.83) | -0.31 (-0.61, -0.01) |
| Kiribati | 0.04 (0.02, 0.06) | 1.57 (0.88, 2.60) | 0.08 (0.04, 0.15) | 1.95 (1.06, 3.56) | 0.83 (0.58, 1.07) |
| Bolivia (Plurinational State of) | 12.52 (7.18, 19.45) | 5.54 (3.18, 8.62) | 41.69 (21.97, 69.29) | 6.03 (3.20, 9.99) | 0.27 (0.22, 0.32) |
| Maldives | 0.20 (0.12, 0.34) | 3.71 (2.20, 6.61) | 0.69 (0.38, 1.25) | 3.16 (1.77, 5.75) | -0.60 (-0.77, -0.43) |
| Grenada | 0.38 (0.26, 0.48) | 5.35 (3.64, 6.73) | 0.34 (0.28, 0.40) | 3.65 (2.98, 4.35) | -0.92 (-1.44, -0.40) |
| Finland | 15.59 (12.47, 19.18) | 2.29 (1.83, 2.82) | 10.71 (8.05, 13.64) | 0.78 (0.59, 0.99) | -3.57 (-3.80, -3.34) |
| Afghanistan | 22.74 (12.61, 39.64) | 4.53 (2.47, 7.89) | 30.50 (17.57, 50.56) | 5.06 (2.91, 8.38) | 0.49 (0.36, 0.63) |
| Switzerland | 18.71 (15.23, 22.68) | 1.82 (1.48, 2.20) | 22.52 (16.45, 29.13) | 1.18 (0.87, 1.53) | -1.56 (-1.75, -1.37) |
| Bangladesh | 106.66 (61.76, 186.57) | 3.10 (1.80, 5.42) | 328.46 (185.39, 557.04) | 2.97 (1.67, 5.02) | -0.29 (-0.37, -0.22) |
| Solomon Islands | 0.13 (0.04, 0.35) | 1.59 (0.47, 4.29) | 0.43 (0.12, 1.10) | 1.99 (0.55, 5.13) | 0.66 (0.54, 0.79) |
