# Supplementary material for: Global, regional, and national burden of malignant neoplasm of bone and articular cartilage in adults aged 65 years and older, 1990–2021: a systematic analysis based on the global burden of disease study 2021
Source: Aging Clin Exp Res. 2025 Jan 8;37(1):21. doi: 10.1007/s40520-024-02926-0 (PMC11711276; doi:10.1007/s40520-024-02926-0)
Supplement: Supplementary file 8 — Supplementary file8 (DOCX 48 KB) [file 40520_2024_2926_MOESM8_ESM.docx]

Table S5 MNBAC DALYs in people aged ≥65 years in 1990 and 2021 for both sexes and EAPC in age-standardized rates by location

| location | Number in 1990 (95% CI) | Rate in 1990 (95% CI) | Number in 2021 (95% CI) | Rate in 2021 (95% CI) | EAPC in age-standardized rates between 1990 and 2021 (95%CI) |
| --- | --- | --- | --- | --- | --- |
| Global | 209290.95 (188853.16, 246597.50) | 63.89 (57.50, 75.12) | 508202.61 (391760.58, 591451.45) | 65.85 (50.82, 76.62) | 0.21 (0.10, 0.33) |
| High SDI | 43260.09 (40221.71, 45561.43) | 41.57 (38.58, 43.81) | 62310.42 (54896.80, 68313.09) | 30.12 (26.69, 32.97) | -1.20 (-1.34, -1.06) |
| High-middle SDI | 75091.88 (67883.20, 85698.99) | 89.91 (81.03, 102.54) | 137454.43 (93553.05, 178686.81) | 74.82 (51.00, 97.17) | -0.51 (-0.64, -0.37) |
| Middle SDI | 50928.79 (41032.83, 72088.89) | 65.57 (52.92, 92.09) | 203201.77 (151310.87, 244887.05) | 88.17 (65.79, 106.09) | 1.35 (1.05, 1.64) |
| Low-middle SDI | 27992.47 (21593.74, 33821.44) | 62.26 (48.11, 75.24) | 80863.98 (65043.16, 96291.88) | 70.06 (56.40, 83.45) | 0.35 (0.32, 0.38) |
| Low SDI | 11672.78 (8996.96, 14626.63) | 69.43 (53.33, 87.18) | 23884.84 (18738.81, 31638.10) | 63.79 (49.97, 84.38) | -0.38 (-0.45, -0.30) |
| Eastern Sub-Saharan Africa | 5894.04 (4578.56, 7757.43) | 104.99 (81.30, 138.37) | 10945.40 (7973.81, 16213.73) | 91.24 (66.54, 134.81) | -0.62 (-0.68, -0.55) |
| Western Sub-Saharan Africa | 3241.67 (2267.91, 4310.28) | 47.59 (33.44, 63.10) | 6103.29 (4581.20, 8006.16) | 44.58 (33.54, 58.50) | -0.26 (-0.32, -0.21) |
| Central Sub-Saharan Africa | 1054.26 (655.77, 1663.60) | 68.76 (42.36, 109.10) | 1951.99 (1097.97, 2992.26) | 55.30 (31.00, 85.18) | -0.82 (-0.94, -0.71) |
| North Africa and Middle East | 8697.22 (6563.51, 11607.14) | 71.14 (53.54, 95.11) | 23524.25 (18373.11, 31223.92) | 69.71 (54.37, 92.75) | 0.01 (-0.06, 0.08) |
| Oceania | 61.65 (34.55, 123.68) | 31.60 (17.93, 62.30) | 170.62 (81.18, 350.32) | 34.87 (16.70, 71.25) | 0.37 (0.18, 0.56) |
| South Asia | 22372.80 (16570.85, 27712.50) | 54.36 (40.23, 67.51) | 70675.74 (56869.18, 91315.51) | 58.53 (47.03, 75.54) | 0.08 (-0.01, 0.17) |
| Southeast Asia | 13538.22 (10780.51, 16973.52) | 71.54 (56.86, 89.85) | 54678.39 (35559.67, 70618.92) | 105.23 (69.01, 135.62) | 1.48 (1.32, 1.64) |
| Caribbean | 1795.76 (1511.73, 2128.81) | 79.66 (66.99, 94.48) | 4525.08 (3694.13, 5448.30) | 95.30 (77.81, 114.73) | 0.37 (0.18, 0.57) |
| Southern Sub-Saharan Africa | 1171.50 (801.57, 1501.52) | 55.48 (37.76, 71.30) | 2394.27 (1952.92, 3019.86) | 53.34 (43.34, 67.01) | -0.40 (-0.70, -0.10) |
| Central Latin America | 4669.86 (4360.30, 4953.29) | 73.20 (68.15, 77.75) | 16088.68 (14242.75, 17986.80) | 76.25 (67.48, 85.22) | 0.31 (0.05, 0.57) |
| Central Asia | 2478.86 (1923.91, 3013.93) | 70.54 (54.65, 85.80) | 4706.09 (3944.75, 5560.21) | 76.26 (63.92, 90.06) | 0.28 (0.08, 0.49) |
| Andean Latin America | 1413.48 (1103.72, 1815.12) | 89.16 (69.62, 114.44) | 3879.38 (2837.48, 5269.20) | 77.62 (56.78, 105.39) | -0.59 (-0.69, -0.49) |
| Tropical Latin America | 7904.94 (7252.55, 8487.89) | 110.57 (100.85, 119.05) | 19709.43 (17788.86, 21342.58) | 88.66 (79.82, 96.09) | -0.47 (-0.64, -0.31) |
| Central Europe | 14011.84 (12386.69, 15678.45) | 105.90 (93.51, 118.44) | 13072.89 (11462.85, 14752.85) | 58.49 (51.28, 66.01) | -2.19 (-2.38, -1.99) |
| Southern Latin America | 5053.64 (4126.58, 6122.39) | 122.63 (100.03, 148.61) | 5225.42 (4416.59, 6141.83) | 64.32 (54.40, 75.57) | -2.14 (-2.36, -1.92) |
| East Asia | 39391.06 (25328.54, 68591.43) | 58.12 (37.42, 101.22) | 199532.65 (119927.87, 270015.61) | 98.55 (59.36, 133.19) | 2.49 (1.82, 3.16) |
| Eastern Europe | 27611.02 (26012.11, 28999.83) | 116.65 (109.69, 122.61) | 11694.02 (10486.81, 12915.84) | 34.56 (31.00, 38.18) | -4.87 (-5.18, -4.57) |
| Western Europe | 32807.02 (30186.10, 34874.13) | 58.76 (54.02, 62.46) | 31440.64 (27292.15, 34823.29) | 33.72 (29.57, 37.21) | -1.88 (-2.08, -1.68) |
| High-income Asia Pacific | 3958.92 (3551.22, 4397.49) | 22.86 (20.41, 25.41) | 6181.44 (5122.52, 7124.34) | 13.12 (11.02, 15.13) | -1.72 (-1.95, -1.49) |
| High-income North America | 11244.45 (10380.06, 11807.10) | 32.68 (30.14, 34.33) | 20569.07 (18351.88, 21989.54) | 31.92 (28.53, 34.11) | -0.27 (-0.50, -0.04) |
| Australasia | 918.74 (791.08, 1058.05) | 41.26 (35.42, 47.60) | 1133.87 (897.56, 1391.11) | 21.42 (17.00, 26.25) | -2.19 (-2.38, -1.99) |
| Mexico | 2449.98 (2322.41, 2572.00) | 75.67 (71.55, 79.52) | 8730.02 (7681.62, 9828.40) | 83.21 (73.23, 93.61) | 0.93 (0.42, 1.44) |
| Guatemala | 206.75 (169.84, 242.86) | 88.57 (71.78, 104.54) | 651.25 (539.63, 783.55) | 71.49 (59.26, 85.93) | -0.65 (-0.88, -0.42) |
| Guinea | 137.47 (77.31, 216.43) | 48.61 (27.42, 76.46) | 210.82 (107.68, 351.59) | 48.99 (25.13, 81.37) | -0.03 (-0.08, 0.02) |
| Gambia | 11.58 (6.45, 19.16) | 43.87 (24.52, 72.17) | 35.47 (18.25, 57.21) | 48.64 (25.05, 78.43) | 0.12 (0.00, 0.25) |
| El Salvador | 98.94 (71.75, 131.28) | 40.43 (29.31, 53.64) | 286.61 (178.15, 430.94) | 51.48 (32.03, 77.27) | 0.77 (0.71, 0.84) |
| Costa Rica | 73.56 (58.79, 91.59) | 51.10 (40.82, 63.63) | 309.99 (235.01, 396.41) | 64.73 (49.12, 82.79) | 1.03 (0.79, 1.26) |
| Cabo Verde | 4.76 (2.66, 8.78) | 22.29 (12.43, 40.97) | 8.06 (4.41, 14.17) | 25.33 (13.82, 44.93) | 0.24 (0.12, 0.37) |
| Peru | 636.27 (401.05, 998.30) | 68.13 (42.88, 106.69) | 1897.52 (1162.09, 2990.04) | 66.60 (40.79, 104.89) | -0.39 (-0.57, -0.21) |
| Paraguay | 205.00 (122.74, 301.90) | 113.31 (67.87, 166.94) | 609.76 (352.65, 943.73) | 127.27 (73.80, 196.80) | 0.44 (0.23, 0.64) |
| Guinea-Bissau | 14.73 (7.90, 26.89) | 49.09 (26.46, 89.16) | 19.78 (10.73, 33.70) | 41.91 (22.75, 71.21) | -0.56 (-0.59, -0.53) |
| Colombia | 1063.96 (897.02, 1257.83) | 79.42 (66.81, 94.03) | 3118.54 (2393.88, 3964.29) | 64.80 (49.83, 82.30) | -1.30 (-1.52, -1.08) |
| Sao Tome and Principe | 1.93 (1.09, 3.51) | 35.66 (20.12, 64.70) | 2.94 (1.64, 4.74) | 38.03 (21.29, 61.54) | 0.07 (0.00, 0.15) |
| Saint Vincent and the Grenadines | 2.58 (2.23, 2.94) | 38.58 (33.34, 44.02) | 16.04 (13.51, 18.98) | 128.65 (108.37, 152.13) | 3.31 (2.05, 4.59) |
| Trinidad and Tobago | 56.05 (48.94, 64.45) | 75.63 (65.94, 87.06) | 95.43 (71.84, 122.07) | 53.46 (40.34, 68.26) | -1.16 (-1.34, -0.97) |
| Jamaica | 175.57 (131.53, 226.74) | 103.53 (77.58, 133.77) | 260.71 (183.66, 357.73) | 98.10 (69.09, 134.68) | -0.05 (-0.34, 0.23) |
| Turkey | 1722.29 (1057.27, 2723.79) | 69.53 (42.72, 109.83) | 4638.42 (2756.11, 6964.25) | 57.91 (34.50, 86.86) | -0.54 (-0.80, -0.28) |
| Bermuda | 2.97 (2.20, 3.88) | 55.29 (41.08, 72.30) | 4.78 (3.51, 6.48) | 35.90 (26.33, 48.65) | -1.35 (-1.60, -1.10) |
| Saudi Arabia | 254.30 (144.50, 407.24) | 64.35 (36.61, 103.02) | 660.51 (377.99, 1045.50) | 67.09 (38.80, 106.29) | 0.07 (-0.02, 0.17) |
| Burundi | 157.16 (82.40, 250.52) | 84.33 (44.26, 134.10) | 245.05 (108.46, 418.35) | 74.09 (33.12, 126.36) | -0.61 (-0.68, -0.53) |
| Morocco | 450.78 (257.23, 746.11) | 39.47 (22.48, 65.21) | 1220.73 (702.02, 1987.87) | 44.75 (25.74, 72.79) | 0.53 (0.41, 0.66) |
| Palestine | 66.99 (35.63, 105.55) | 98.33 (52.40, 154.96) | 187.78 (118.88, 291.15) | 108.88 (68.99, 168.91) | 0.61 (0.46, 0.77) |
| Bahrain | 11.12 (7.02, 18.79) | 108.89 (68.66, 183.56) | 49.67 (29.73, 85.22) | 102.09 (61.52, 175.38) | -0.38 (-0.49, -0.27) |
| Algeria | 676.63 (403.55, 1046.62) | 72.89 (43.81, 113.79) | 1886.54 (965.65, 3064.59) | 69.93 (35.84, 113.53) | -0.08 (-0.18, 0.01) |
| Central African Republic | 50.79 (26.94, 89.72) | 67.87 (35.99, 120.10) | 70.20 (33.60, 112.20) | 54.89 (25.88, 88.55) | -0.78 (-0.83, -0.73) |
| Syrian Arab Republic | 71.99 (37.20, 139.80) | 19.44 (10.04, 37.66) | 202.10 (104.64, 378.33) | 20.80 (10.78, 39.38) | 0.06 (-0.02, 0.14) |
| Equatorial Guinea | 8.83 (4.52, 15.99) | 64.71 (32.79, 117.63) | 21.01 (10.27, 35.75) | 62.44 (30.66, 105.25) | 0.02 (-0.06, 0.10) |
| Iran (Islamic Republic of) | 891.26 (697.11, 1147.42) | 48.77 (38.09, 62.52) | 2647.29 (2047.34, 3517.85) | 44.86 (34.67, 59.63) | -0.21 (-0.27, -0.15) |
| Jordan | 65.73 (37.99, 101.59) | 75.73 (43.49, 117.24) | 395.32 (236.59, 641.44) | 78.80 (47.13, 127.57) | 0.18 (0.01, 0.35) |
| Nepal | 335.45 (199.78, 545.20) | 50.38 (29.93, 81.91) | 1070.05 (614.02, 1718.38) | 55.78 (32.00, 89.39) | 0.37 (0.21, 0.52) |
| Congo | 60.08 (33.60, 96.35) | 76.08 (42.56, 122.93) | 115.73 (59.87, 185.68) | 66.59 (34.58, 106.67) | -0.61 (-0.70, -0.53) |
| Democratic Republic of the Congo | 744.74 (408.15, 1276.81) | 69.56 (37.57, 120.96) | 1280.39 (597.63, 2125.49) | 53.92 (25.14, 89.79) | -0.94 (-1.08, -0.79) |
| Libya | 74.24 (41.30, 122.48) | 53.21 (29.55, 87.90) | 212.47 (119.44, 350.86) | 60.76 (34.18, 100.16) | 0.73 (0.61, 0.85) |
| Comoros | 13.65 (6.87, 21.81) | 91.97 (46.43, 146.35) | 33.06 (14.59, 60.27) | 88.33 (39.05, 160.66) | -0.25 (-0.31, -0.20) |
| Lebanon | 117.42 (67.16, 183.43) | 71.80 (41.11, 111.91) | 383.82 (211.49, 585.55) | 68.80 (37.81, 104.94) | -0.01 (-0.26, 0.24) |
| Kuwait | 17.66 (13.61, 22.23) | 51.81 (39.90, 65.28) | 86.57 (61.48, 117.08) | 51.80 (36.77, 70.14) | 0.64 (-0.71, 2.01) |
| Gabon | 33.58 (17.80, 55.86) | 71.66 (37.90, 119.81) | 50.98 (25.89, 84.47) | 67.85 (34.62, 112.31) | -0.37 (-0.48, -0.26) |
| Sudan | 406.52 (219.63, 698.72) | 53.54 (28.81, 92.28) | 804.33 (474.55, 1343.96) | 57.99 (34.23, 96.87) | 0.28 (0.21, 0.35) |
| Djibouti | 6.77 (3.26, 11.44) | 82.20 (39.91, 138.03) | 37.72 (17.12, 68.64) | 92.39 (41.86, 165.52) | 0.36 (0.28, 0.43) |
| Eritrea | 52.16 (25.27, 80.20) | 85.69 (41.78, 132.81) | 148.54 (65.64, 255.35) | 86.28 (38.30, 148.48) | -0.09 (-0.14, -0.04) |
| Kenya | 579.13 (432.24, 755.14) | 92.58 (68.98, 120.66) | 1708.27 (1250.26, 2406.19) | 101.81 (74.54, 143.01) | 0.40 (0.36, 0.45) |
| Iraq | 618.36 (330.89, 964.81) | 99.69 (53.34, 155.50) | 1755.10 (1033.16, 2707.19) | 104.68 (61.89, 160.50) | 0.30 (0.21, 0.39) |
| Malawi | 248.44 (124.84, 378.00) | 85.07 (42.81, 129.75) | 431.45 (207.41, 729.58) | 78.42 (37.61, 132.67) | -0.33 (-0.40, -0.27) |
| Angola | 156.24 (79.96, 288.00) | 63.30 (32.37, 117.34) | 413.67 (197.87, 679.47) | 55.49 (26.49, 91.58) | -0.54 (-0.64, -0.44) |
| Seychelles | 1.00 (0.28, 2.24) | 19.32 (5.41, 43.39) | 1.68 (0.41, 3.64) | 18.60 (4.51, 40.30) | 0.04 (-0.38, 0.46) |
| Madagascar | 315.82 (163.55, 496.94) | 82.08 (42.66, 128.49) | 465.79 (206.36, 798.89) | 66.23 (29.59, 113.04) | -0.62 (-0.81, -0.43) |
| Ethiopia | 2062.32 (1454.24, 2911.55) | 141.42 (99.22, 200.59) | 3432.08 (2342.00, 4769.70) | 105.30 (71.98, 146.27) | -1.29 (-1.43, -1.16) |
| Mozambique | 397.65 (196.71, 618.92) | 91.64 (45.36, 142.79) | 683.32 (313.88, 1128.46) | 87.85 (40.24, 145.78) | -0.04 (-0.12, 0.04) |
| Rwanda | 207.97 (105.58, 318.03) | 98.41 (49.84, 150.21) | 357.65 (158.23, 654.40) | 78.40 (35.01, 143.21) | -1.09 (-1.24, -0.94) |
| Uganda | 502.32 (298.16, 756.76) | 100.47 (59.56, 151.49) | 940.08 (493.57, 1521.65) | 91.55 (48.25, 148.10) | -0.65 (-0.80, -0.50) |
| Mauritius | 67.97 (59.75, 76.97) | 112.72 (99.05, 127.54) | 167.24 (145.31, 190.19) | 99.66 (86.52, 113.27) | 0.97 (-0.54, 2.49) |
| Somalia | 126.83 (61.90, 201.97) | 95.21 (46.19, 151.41) | 338.39 (141.87, 567.30) | 83.82 (35.21, 141.76) | -0.38 (-0.41, -0.34) |
| United Republic of Tanzania | 810.14 (419.88, 1240.34) | 94.84 (49.33, 145.27) | 1501.22 (688.46, 2643.78) | 80.44 (37.05, 141.32) | -0.67 (-0.72, -0.61) |
| Eswatini | 19.22 (11.14, 30.58) | 94.74 (54.85, 150.50) | 41.04 (20.96, 69.95) | 101.86 (51.92, 174.51) | 0.27 (-0.07, 0.61) |
| Zambia | 191.67 (95.80, 294.91) | 94.91 (47.81, 146.20) | 378.00 (167.06, 671.15) | 81.27 (36.36, 143.85) | -0.72 (-0.80, -0.63) |
| Chad | 93.47 (49.50, 173.18) | 37.79 (19.98, 70.18) | 170.31 (80.52, 313.03) | 40.96 (19.46, 75.02) | 0.23 (0.19, 0.26) |
| Benin | 72.85 (42.75, 129.32) | 44.14 (25.92, 78.25) | 146.99 (79.78, 251.01) | 39.41 (21.41, 67.27) | -0.48 (-0.55, -0.40) |
| Coted'Ivoire | 94.98 (56.40, 168.57) | 38.39 (22.98, 67.53) | 254.81 (147.77, 443.14) | 34.92 (20.20, 60.35) | -0.37 (-0.43, -0.31) |
| Ghana | 164.78 (89.37, 314.56) | 36.83 (20.04, 69.85) | 500.55 (266.83, 835.20) | 41.60 (22.20, 69.37) | 0.41 (0.38, 0.44) |
| Cameroon | 176.40 (107.00, 301.53) | 55.98 (33.93, 95.28) | 407.54 (217.24, 696.84) | 46.97 (25.14, 80.03) | -0.65 (-0.72, -0.59) |
| Liberia | 50.58 (29.48, 95.40) | 50.36 (29.22, 94.69) | 56.62 (31.06, 96.29) | 41.04 (22.51, 69.62) | -0.59 (-0.74, -0.43) |
| Niger | 85.65 (47.11, 158.00) | 44.41 (24.34, 81.62) | 222.32 (112.19, 384.32) | 37.94 (19.10, 65.46) | -0.59 (-0.65, -0.54) |
| Mali | 123.86 (74.20, 205.63) | 42.03 (25.33, 69.51) | 250.57 (141.12, 412.31) | 39.09 (22.09, 64.40) | -0.23 (-0.28, -0.18) |
| Sierra Leone | 65.56 (34.25, 121.75) | 37.63 (19.69, 69.84) | 96.01 (49.78, 169.69) | 34.14 (17.80, 60.03) | -0.40 (-0.47, -0.34) |
| Senegal | 114.80 (64.78, 203.91) | 44.78 (25.29, 79.51) | 246.68 (127.48, 420.17) | 41.96 (21.79, 71.36) | -0.23 (-0.31, -0.14) |
| Cook Islands | 0.19 (0.11, 0.34) | 19.15 (10.81, 34.96) | 0.39 (0.20, 0.75) | 16.33 (8.59, 32.05) | -0.71 (-0.83, -0.58) |
| Greenland | 0.79 (0.36, 1.42) | 37.38 (17.71, 66.11) | 0.94 (0.53, 1.52) | 17.72 (9.92, 28.62) | -2.18 (-2.56, -1.81) |
| Guam | 1.21 (0.74, 1.92) | 23.65 (14.43, 37.56) | 2.69 (1.68, 4.03) | 14.27 (8.92, 21.30) | -1.25 (-1.45, -1.05) |
| Niue | 0.08 (0.03, 0.18) | 38.03 (15.69, 86.60) | 0.09 (0.03, 0.20) | 46.57 (15.42, 105.54) | 0.64 (0.52, 0.76) |
| Palau | 0.06 (0.02, 0.13) | 6.95 (2.71, 16.53) | 0.12 (0.05, 0.28) | 7.25 (2.85, 17.08) | 0.36 (0.18, 0.53) |
| Nigeria | 1794.30 (1225.94, 2547.24) | 51.36 (35.21, 72.43) | 3033.04 (2186.62, 4275.98) | 49.09 (35.52, 68.93) | -0.18 (-0.28, -0.07) |
| Northern Mariana Islands | 0.27 (0.15, 0.45) | 32.04 (17.20, 53.18) | 1.25 (0.77, 2.08) | 33.27 (20.39, 55.09) | 0.62 (0.17, 1.07) |
| Mauritania | 42.74 (25.11, 74.27) | 49.34 (29.07, 85.53) | 78.67 (40.70, 135.85) | 46.53 (24.19, 80.14) | -0.40 (-0.46, -0.35) |
| Togo | 35.76 (20.36, 64.22) | 40.61 (23.15, 73.04) | 95.30 (51.35, 167.30) | 37.13 (20.12, 64.92) | -0.48 (-0.54, -0.41) |
| Saint Kitts and Nevis | 4.19 (3.61, 4.84) | 105.63 (90.66, 122.04) | 3.88 (3.12, 4.71) | 75.71 (61.33, 91.23) | -0.48 (-0.85, -0.11) |
| American Samoa | 0.96 (0.51, 1.54) | 61.70 (32.75, 98.28) | 2.74 (1.66, 4.48) | 74.64 (45.04, 122.06) | 1.01 (0.75, 1.28) |
| Puerto Rico | 81.40 (63.36, 102.92) | 24.12 (18.76, 30.50) | 271.00 (201.56, 354.27) | 37.29 (27.76, 48.83) | 1.57 (0.25, 2.91) |
| Monaco | 0.47 (0.16, 1.02) | 6.51 (2.15, 14.12) | 0.57 (0.22, 1.15) | 5.75 (2.20, 11.58) | -0.66 (-0.81, -0.52) |
| Nauru | 0.13 (0.06, 0.27) | 43.17 (17.91, 89.70) | 0.22 (0.07, 0.49) | 54.63 (17.77, 119.28) | 0.68 (0.57, 0.78) |
| Tokelau | 0.04 (0.02, 0.10) | 35.66 (13.06, 87.06) | 0.06 (0.02, 0.14) | 42.58 (13.07, 99.05) | 0.61 (0.50, 0.72) |
| San Marino | 1.98 (1.08, 3.09) | 57.88 (31.51, 90.37) | 1.96 (0.95, 3.39) | 26.30 (12.76, 45.49) | -1.86 (-2.16, -1.55) |
| South Sudan | 217.81 (102.13, 352.82) | 98.33 (46.28, 159.16) | 235.27 (97.98, 432.77) | 95.61 (39.89, 175.14) | -0.17 (-0.21, -0.13) |
| Tuvalu | 0.18 (0.07, 0.45) | 34.49 (12.31, 86.50) | 0.37 (0.11, 0.92) | 42.59 (12.73, 104.60) | 0.66 (0.53, 0.79) |
| United States Virgin Islands | 1.38 (0.85, 2.28) | 21.49 (13.28, 35.65) | 3.40 (1.73, 6.41) | 18.14 (9.19, 34.43) | -0.11 (-0.28, 0.06) |
| Taiwan (Province of China) | 1159.48 (983.83, 1347.40) | 87.64 (74.09, 102.12) | 1398.55 (1086.97, 1763.26) | 35.34 (27.48, 44.56) | -2.56 (-3.07, -2.06) |
| Uzbekistan | 697.02 (357.94, 1046.26) | 81.25 (42.00, 121.72) | 1722.49 (1258.10, 2271.22) | 91.25 (66.24, 120.72) | 0.61 (0.13, 1.08) |
| Honduras | 54.89 (32.83, 93.81) | 34.74 (20.63, 59.63) | 297.45 (151.95, 558.80) | 58.74 (30.03, 110.37) | 1.79 (1.62, 1.96) |
| France | 6437.75 (5374.62, 7388.23) | 81.43 (68.05, 93.48) | 6312.32 (4817.07, 7988.39) | 43.35 (33.18, 54.84) | -2.04 (-2.23, -1.85) |
| Myanmar | 1200.05 (684.23, 2140.14) | 67.25 (38.23, 120.08) | 3565.97 (1579.02, 7215.29) | 91.99 (41.09, 184.92) | 1.07 (0.92, 1.22) |
| Bahamas | 6.84 (5.86, 7.95) | 55.16 (47.15, 64.18) | 15.47 (12.28, 19.13) | 48.62 (38.64, 60.00) | -0.37 (-0.67, -0.07) |
| South Africa | 780.63 (452.97, 1057.72) | 48.41 (28.00, 65.74) | 1697.72 (1355.94, 2067.90) | 46.55 (36.99, 56.71) | -0.50 (-0.81, -0.19) |
| Guyana | 25.62 (21.47, 30.23) | 86.42 (72.30, 102.05) | 38.99 (29.98, 49.59) | 76.41 (58.99, 96.91) | -0.07 (-0.25, 0.12) |
| Bhutan | 8.78 (4.91, 14.23) | 53.13 (29.63, 86.25) | 32.42 (17.60, 52.50) | 64.51 (35.03, 104.49) | 0.61 (0.57, 0.64) |
| Hungary | 1113.71 (933.29, 1311.85) | 80.32 (67.43, 94.68) | 620.97 (477.56, 806.55) | 30.91 (23.78, 40.13) | -3.37 (-3.73, -3.02) |
| Philippines | 2733.15 (2168.79, 3937.28) | 126.61 (99.97, 183.35) | 10224.48 (7907.79, 14221.74) | 161.79 (124.74, 226.90) | 0.99 (0.75, 1.24) |
| Argentina | 3883.49 (3031.35, 4903.20) | 133.41 (104.06, 168.43) | 3699.59 (2987.15, 4538.71) | 70.35 (56.83, 86.29) | -2.14 (-2.39, -1.89) |
| North Macedonia | 241.14 (180.52, 317.75) | 162.67 (121.79, 214.24) | 383.94 (238.02, 584.57) | 126.64 (78.12, 192.77) | -0.99 (-1.22, -0.76) |
| Germany | 3661.04 (2972.41, 4433.93) | 30.45 (24.71, 36.88) | 4536.51 (3611.29, 5540.59) | 24.08 (19.34, 29.30) | -1.02 (-1.18, -0.85) |
| Andorra | 0.35 (0.13, 0.77) | 6.98 (2.55, 15.08) | 0.60 (0.23, 1.31) | 4.34 (1.67, 9.44) | -1.37 (-1.55, -1.19) |
| Nicaragua | 60.56 (37.10, 90.29) | 52.04 (31.90, 77.61) | 257.53 (163.38, 405.21) | 65.90 (41.69, 103.80) | 0.99 (0.71, 1.28) |
| Greece | 2740.12 (2454.75, 3026.43) | 193.57 (173.08, 214.02) | 1797.18 (1531.74, 2061.64) | 74.00 (63.76, 84.61) | -4.02 (-4.42, -3.61) |
| Chile | 1096.42 (845.86, 1382.90) | 131.07 (101.12, 165.27) | 1447.68 (1155.16, 1778.77) | 62.17 (49.62, 76.37) | -2.39 (-2.58, -2.21) |
| Barbados | 20.76 (17.65, 23.92) | 69.89 (59.44, 80.59) | 30.06 (23.10, 37.04) | 61.53 (47.35, 75.78) | 0.02 (-0.14, 0.18) |
| Azerbaijan | 160.66 (93.07, 294.06) | 46.60 (26.99, 85.35) | 332.48 (197.07, 585.55) | 46.19 (27.42, 81.36) | 0.00 (-0.09, 0.09) |
| Ecuador | 543.11 (426.40, 686.93) | 133.24 (104.27, 168.83) | 1212.82 (895.50, 1605.95) | 87.04 (64.37, 115.02) | -1.32 (-1.45, -1.19) |
| Haiti | 183.32 (102.81, 326.84) | 76.58 (42.17, 136.95) | 356.26 (182.57, 646.75) | 70.90 (36.22, 129.78) | -0.15 (-0.22, -0.09) |
| Brunei Darussalam | 1.34 (0.83, 2.22) | 18.30 (11.35, 30.47) | 3.89 (2.34, 6.58) | 15.90 (9.45, 27.07) | 0.25 (0.01, 0.48) |
| India | 17251.86 (12584.87, 21695.93) | 53.21 (38.71, 67.20) | 57315.18 (45948.46, 74552.64) | 58.14 (46.54, 75.57) | 0.13 (0.02, 0.24) |
| Japan | 3127.49 (2892.66, 3298.70) | 20.87 (19.21, 22.05) | 4547.30 (3872.99, 4983.85) | 12.01 (10.49, 13.02) | -1.56 (-1.88, -1.23) |
| Armenia | 142.50 (99.77, 193.25) | 76.47 (53.46, 103.76) | 303.02 (220.70, 410.96) | 76.29 (55.51, 103.52) | 0.24 (0.01, 0.47) |
| Zimbabwe | 251.76 (140.79, 377.05) | 80.65 (45.06, 121.46) | 407.57 (210.41, 658.92) | 82.30 (42.34, 132.59) | 0.06 (-0.25, 0.38) |
| Kazakhstan | 350.58 (238.42, 466.28) | 36.71 (24.92, 48.92) | 345.81 (246.38, 464.38) | 23.81 (16.98, 32.01) | -1.94 (-2.33, -1.54) |
| Georgia | 394.44 (266.01, 544.63) | 76.95 (51.92, 106.41) | 721.72 (524.36, 958.32) | 129.11 (93.83, 171.40) | 1.85 (1.28, 2.42) |
| Thailand | 1855.84 (1085.65, 2825.26) | 73.15 (42.84, 111.12) | 11072.16 (6756.92, 17627.59) | 114.95 (70.25, 182.80) | 2.11 (1.87, 2.35) |
| Oman | 9.93 (5.40, 17.26) | 21.27 (11.53, 36.97) | 28.11 (17.35, 46.47) | 24.65 (15.16, 40.78) | 0.50 (0.32, 0.67) |
| Bulgaria | 883.63 (671.72, 1110.71) | 78.45 (59.80, 98.42) | 952.97 (703.21, 1251.84) | 64.70 (47.71, 85.11) | -0.15 (-0.50, 0.21) |
| China | 37293.23 (23180.67, 66715.62) | 57.14 (35.55, 102.19) | 195512.24 (117166.85, 265469.69) | 99.91 (59.98, 135.48) | 2.59 (1.90, 3.29) |
| Denmark | 298.56 (244.73, 359.15) | 37.11 (30.43, 44.63) | 326.61 (253.18, 412.96) | 27.30 (21.16, 34.55) | -1.78 (-2.12, -1.44) |
| Uruguay | 73.50 (56.13, 94.68) | 19.85 (15.15, 25.57) | 77.86 (59.82, 100.28) | 14.67 (11.28, 18.91) | -1.13 (-1.49, -0.76) |
| Malaysia | 316.16 (178.88, 568.15) | 46.41 (26.24, 83.40) | 1387.58 (665.09, 2654.40) | 58.69 (28.06, 112.62) | 0.70 (0.57, 0.82) |
| Panama | 45.06 (39.08, 51.75) | 36.51 (31.62, 41.94) | 244.75 (184.59, 302.66) | 62.89 (47.46, 77.76) | 1.54 (1.22, 1.87) |
| Norway | 200.15 (181.03, 218.97) | 28.43 (25.72, 31.11) | 277.21 (239.59, 312.93) | 28.15 (24.41, 31.76) | -0.76 (-1.18, -0.34) |
| Poland | 4667.19 (4383.47, 4942.10) | 119.78 (112.19, 127.01) | 3312.62 (2950.44, 3642.23) | 46.08 (41.05, 50.67) | -3.49 (-3.76, -3.22) |
| Micronesia (Federated States of) | 1.65 (0.71, 3.70) | 40.88 (17.48, 91.35) | 2.43 (0.76, 5.69) | 51.79 (16.36, 120.65) | 0.71 (0.59, 0.83) |
| Ireland | 272.50 (222.32, 331.75) | 67.62 (55.09, 82.36) | 250.29 (188.31, 319.03) | 32.98 (24.83, 42.04) | -2.26 (-2.68, -1.85) |
| Estonia | 96.61 (78.40, 117.81) | 53.30 (43.25, 65.05) | 70.00 (52.95, 89.50) | 26.30 (19.87, 33.68) | -3.33 (-3.84, -2.83) |
| Croatia | 474.83 (389.36, 568.21) | 96.18 (79.00, 115.00) | 689.45 (542.47, 860.88) | 76.89 (60.46, 96.10) | -1.25 (-1.67, -0.83) |
| Israel | 242.31 (187.49, 307.30) | 52.52 (40.64, 66.55) | 343.04 (261.59, 433.58) | 28.38 (21.67, 35.87) | -2.26 (-2.44, -2.09) |
| Viet Nam | 2644.91 (1414.29, 4076.69) | 78.41 (42.02, 120.79) | 7391.95 (4042.35, 11336.42) | 96.17 (53.06, 147.79) | 0.73 (0.60, 0.85) |
| United States of America | 10021.83 (9230.40, 10515.98) | 31.97 (29.43, 33.56) | 18802.02 (16818.62, 20053.63) | 32.82 (29.40, 34.99) | -0.14 (-0.39, 0.10) |
| Brazil | 7699.94 (7056.69, 8259.80) | 110.49 (100.67, 118.83) | 19099.67 (17187.24, 20727.00) | 87.81 (78.84, 95.39) | -0.49 (-0.67, -0.32) |
| Belarus | 882.79 (697.85, 1096.63) | 80.34 (63.46, 99.82) | 634.23 (462.15, 847.08) | 42.56 (31.09, 56.71) | -2.25 (-2.36, -2.14) |
| Republic of Korea | 766.70 (488.58, 1154.20) | 36.03 (22.95, 54.52) | 1509.22 (909.65, 2218.69) | 17.85 (10.75, 26.25) | -2.76 (-3.00, -2.53) |
| Canada | 1221.57 (1043.12, 1409.16) | 39.89 (34.00, 46.08) | 1765.79 (1393.04, 2152.28) | 24.72 (19.54, 30.12) | -1.40 (-1.57, -1.23) |
| Iceland | 13.17 (10.53, 16.04) | 48.70 (38.95, 59.28) | 23.25 (17.58, 29.51) | 41.31 (31.36, 52.45) | -1.30 (-1.70, -0.90) |
| Romania | 3503.68 (2531.44, 4641.05) | 145.15 (106.06, 190.89) | 3270.81 (2520.75, 4163.91) | 86.62 (66.73, 110.34) | -1.88 (-2.03, -1.73) |
| Qatar | 2.86 (1.73, 4.84) | 64.70 (39.12, 109.68) | 19.66 (11.70, 33.00) | 58.40 (34.76, 98.86) | -0.14 (-0.29, 0.00) |
| Portugal | 1272.43 (1081.47, 1490.96) | 96.75 (81.96, 113.67) | 1261.74 (977.44, 1582.94) | 50.15 (38.98, 62.94) | -2.69 (-3.10, -2.29) |
| Montenegro | 31.56 (17.58, 49.68) | 63.16 (35.16, 99.23) | 49.29 (34.13, 73.41) | 53.24 (36.76, 79.26) | -0.76 (-0.92, -0.60) |
| Sri Lanka | 305.58 (166.07, 456.09) | 37.07 (20.20, 55.33) | 1341.58 (659.70, 2565.97) | 54.15 (26.69, 103.50) | 2.07 (1.63, 2.52) |
| Dominican Republic | 251.71 (121.36, 402.97) | 88.12 (42.47, 141.02) | 873.03 (452.28, 1394.54) | 103.62 (53.70, 165.54) | 0.76 (0.61, 0.91) |
| Venezuela (Bolivarian Republic of) | 616.14 (530.36, 704.30) | 82.88 (71.19, 94.85) | 2192.54 (1644.21, 2839.94) | 86.10 (64.74, 111.28) | 0.16 (-0.04, 0.37) |
| Democratic People's Republic of Korea | 938.36 (535.77, 1668.14) | 81.29 (46.82, 143.89) | 2621.85 (1252.38, 5241.77) | 96.92 (46.29, 193.62) | 0.77 (0.60, 0.94) |
| Yemen | 174.40 (85.36, 313.58) | 49.01 (23.76, 88.93) | 521.08 (269.79, 965.52) | 51.47 (26.62, 95.09) | 0.20 (0.14, 0.26) |
| Suriname | 14.16 (7.81, 21.44) | 72.58 (39.97, 109.81) | 32.61 (18.34, 52.97) | 62.86 (35.32, 102.07) | -0.17 (-0.26, -0.09) |
| Italy | 7624.31 (6937.11, 8181.84) | 89.45 (81.22, 96.12) | 5376.41 (4577.01, 6050.21) | 36.81 (31.79, 41.25) | -2.52 (-3.01, -2.03) |
| Indonesia | 3997.61 (2541.82, 5479.32) | 57.15 (36.48, 78.21) | 18043.21 (8070.71, 28993.03) | 100.80 (45.74, 161.07) | 1.93 (1.79, 2.07) |
| Egypt | 2370.82 (1320.78, 3746.86) | 137.07 (75.36, 216.68) | 6500.56 (4045.02, 9578.57) | 154.32 (93.68, 231.21) | 0.45 (0.30, 0.59) |
| Papua New Guinea | 28.24 (7.88, 79.85) | 23.70 (6.48, 66.84) | 87.86 (19.25, 245.26) | 27.80 (6.01, 77.92) | 0.49 (0.33, 0.66) |
| Latvia | 213.69 (173.03, 260.88) | 67.36 (54.55, 82.27) | 181.12 (138.95, 232.40) | 47.22 (36.21, 60.68) | -1.79 (-2.24, -1.33) |
| Serbia | 1061.46 (613.52, 1836.02) | 121.34 (69.79, 208.41) | 1453.80 (898.62, 2168.28) | 87.98 (54.34, 131.35) | -1.48 (-1.62, -1.34) |
| Cyprus | 52.53 (27.75, 80.36) | 73.32 (38.21, 113.80) | 88.00 (51.31, 132.85) | 45.51 (26.36, 69.17) | -1.55 (-1.69, -1.41) |
| Bosnia and Herzegovina | 188.16 (97.00, 357.06) | 64.07 (33.17, 121.61) | 301.56 (183.66, 491.08) | 49.76 (30.33, 80.99) | -1.00 (-1.08, -0.91) |
| Pakistan | 2775.16 (1882.45, 4035.75) | 60.64 (41.04, 88.23) | 6169.66 (4026.47, 8994.79) | 72.04 (47.10, 105.01) | 0.44 (0.33, 0.55) |
| Tonga | 1.32 (0.52, 3.05) | 30.21 (11.76, 69.69) | 2.78 (0.83, 6.40) | 41.56 (12.42, 95.59) | 1.03 (0.86, 1.21) |
| Lesotho | 46.00 (26.25, 75.82) | 65.88 (37.57, 108.51) | 82.62 (45.77, 134.17) | 97.33 (53.71, 159.11) | 1.68 (1.42, 1.95) |
| Kyrgyzstan | 194.76 (143.06, 258.55) | 85.21 (62.58, 113.12) | 486.10 (349.21, 663.32) | 139.71 (100.93, 189.43) | 1.84 (1.33, 2.35) |
| Luxembourg | 29.25 (25.66, 33.00) | 58.31 (51.09, 65.86) | 34.92 (29.48, 40.43) | 35.60 (30.14, 41.17) | -1.63 (-1.80, -1.47) |
| Cambodia | 255.95 (146.84, 450.58) | 76.04 (43.66, 133.16) | 990.67 (426.30, 1959.36) | 103.46 (44.99, 203.75) | 1.11 (1.04, 1.18) |
| Slovakia | 412.20 (283.88, 627.05) | 74.71 (51.38, 113.54) | 503.56 (310.67, 791.62) | 53.70 (33.09, 84.35) | -1.28 (-1.36, -1.20) |
| Samoa | 6.24 (2.59, 11.43) | 95.34 (39.86, 174.09) | 11.15 (5.40, 18.30) | 100.75 (48.73, 165.42) | 0.26 (0.13, 0.39) |
| Tunisia | 218.10 (120.19, 339.76) | 55.35 (30.64, 86.42) | 621.05 (332.19, 1003.22) | 55.78 (29.95, 89.94) | 0.09 (0.06, 0.13) |
| Spain | 3979.34 (3447.57, 4548.08) | 76.26 (65.95, 87.25) | 3532.30 (2739.29, 4421.45) | 37.23 (29.11, 46.48) | -2.08 (-2.44, -1.72) |
| Saint Lucia | 4.99 (4.31, 5.75) | 63.51 (54.53, 73.45) | 9.11 (7.28, 11.19) | 44.43 (35.52, 54.57) | -1.40 (-1.61, -1.20) |
| Timor-Leste | 10.19 (5.76, 18.37) | 62.56 (35.47, 112.12) | 62.74 (26.71, 126.17) | 82.17 (35.04, 165.52) | 1.04 (0.97, 1.10) |
| Dominica | 4.64 (2.58, 7.10) | 81.48 (45.30, 125.11) | 7.04 (4.02, 11.02) | 98.64 (56.04, 154.47) | 0.79 (0.54, 1.05) |
| Lao People's Democratic Republic | 126.24 (69.83, 226.87) | 76.86 (42.61, 137.96) | 340.51 (143.29, 705.69) | 101.61 (43.24, 209.21) | 0.97 (0.86, 1.08) |
| Lithuania | 225.77 (184.14, 273.41) | 57.22 (46.67, 69.19) | 166.98 (128.05, 211.93) | 30.96 (23.72, 39.34) | -2.73 (-3.18, -2.27) |
| Burkina Faso | 155.34 (85.05, 300.06) | 45.13 (24.58, 86.83) | 266.75 (138.06, 464.87) | 38.11 (19.76, 66.14) | -0.65 (-0.77, -0.53) |
| Sweden | 591.41 (488.30, 704.16) | 37.85 (31.24, 45.10) | 392.53 (308.44, 489.22) | 17.70 (13.96, 22.07) | -2.08 (-2.33, -1.83) |
| Belgium | 966.50 (783.11, 1182.75) | 65.86 (53.34, 80.64) | 945.01 (727.45, 1186.20) | 41.02 (31.78, 51.43) | -1.63 (-2.02, -1.24) |
| United Arab Emirates | 20.36 (11.41, 36.23) | 94.00 (52.54, 169.16) | 101.87 (52.50, 171.15) | 86.01 (42.58, 145.68) | 1.74 (1.15, 2.33) |
| Albania | 228.04 (148.36, 322.31) | 142.35 (92.39, 201.73) | 413.86 (189.79, 712.80) | 99.98 (46.07, 171.88) | -1.29 (-1.44, -1.15) |
| Fiji | 12.14 (5.85, 19.97) | 50.82 (24.45, 83.91) | 35.32 (19.74, 55.79) | 62.17 (34.65, 98.36) | 0.55 (0.22, 0.88) |
| United Kingdom | 2662.38 (2506.25, 2781.40) | 29.62 (27.85, 30.97) | 3814.31 (3435.98, 4071.40) | 29.59 (26.85, 31.51) | 0.10 (-0.18, 0.37) |
| Cuba | 884.91 (694.65, 1107.68) | 94.98 (74.53, 118.95) | 2331.37 (1816.48, 2942.98) | 131.57 (102.47, 166.17) | 0.50 (0.23, 0.77) |
| Marshall Islands | 0.38 (0.13, 0.99) | 29.96 (9.96, 77.35) | 0.89 (0.25, 2.21) | 39.69 (11.19, 97.64) | 0.76 (0.57, 0.96) |
| Belize | 4.68 (3.45, 5.74) | 58.78 (43.32, 72.05) | 11.85 (9.90, 14.00) | 52.87 (44.20, 62.39) | -0.35 (-0.66, -0.04) |
| Slovenia | 107.48 (86.58, 131.31) | 49.74 (40.09, 60.63) | 132.91 (97.41, 176.38) | 30.24 (22.17, 40.19) | -1.89 (-2.32, -1.45) |
| Republic of Moldova | 458.89 (379.20, 542.24) | 122.61 (101.46, 144.62) | 272.81 (233.74, 316.86) | 48.50 (41.59, 56.28) | -3.51 (-3.92, -3.09) |
| Ukraine | 2516.83 (1947.39, 3244.61) | 39.50 (30.56, 50.97) | 2471.84 (1735.94, 3365.58) | 33.00 (23.18, 44.93) | -0.79 (-1.13, -0.45) |
| Austria | 419.17 (342.16, 504.21) | 36.25 (29.51, 43.64) | 549.41 (424.75, 691.15) | 31.18 (24.24, 39.19) | -0.91 (-1.26, -0.56) |
| Mongolia | 112.52 (63.46, 180.49) | 127.07 (71.70, 203.00) | 151.14 (93.39, 221.36) | 100.67 (61.89, 147.67) | -0.95 (-1.10, -0.80) |
| Netherlands | 694.01 (576.33, 827.56) | 36.25 (30.08, 43.25) | 972.67 (761.55, 1213.13) | 27.72 (21.70, 34.57) | -1.29 (-1.52, -1.06) |
| Antigua and Barbuda | 2.56 (2.11, 3.03) | 48.82 (40.35, 57.80) | 4.48 (3.84, 5.19) | 51.01 (43.69, 59.17) | 0.34 (0.06, 0.62) |
| Malta | 28.67 (22.46, 35.57) | 73.30 (57.42, 90.92) | 42.26 (31.85, 54.43) | 41.77 (31.51, 53.81) | -1.98 (-2.20, -1.76) |
| Vanuatu | 1.28 (0.42, 3.23) | 29.60 (9.74, 75.16) | 4.78 (1.34, 11.84) | 39.42 (11.08, 98.00) | 0.89 (0.76, 1.02) |
| Singapore | 63.39 (50.29, 77.94) | 36.76 (29.15, 45.21) | 121.04 (92.48, 153.20) | 15.97 (12.20, 20.20) | -2.70 (-3.03, -2.37) |
| Turkmenistan | 176.57 (112.71, 253.50) | 124.84 (79.29, 179.55) | 296.17 (182.51, 448.40) | 101.09 (62.28, 153.10) | -0.98 (-1.11, -0.84) |
| Australia | 748.54 (629.38, 879.78) | 40.55 (34.01, 47.74) | 932.08 (714.52, 1168.30) | 20.78 (15.96, 26.02) | -2.18 (-2.39, -1.97) |
| New Zealand | 170.21 (137.76, 207.42) | 44.93 (36.29, 54.79) | 201.79 (157.50, 253.60) | 24.78 (19.36, 31.13) | -2.21 (-2.55, -1.86) |
| Czechia | 874.71 (739.97, 1025.64) | 67.17 (56.82, 78.79) | 796.88 (620.68, 1000.47) | 36.03 (28.03, 45.30) | -2.03 (-2.32, -1.74) |
| Tajikistan | 249.83 (142.57, 405.36) | 122.71 (70.15, 198.93) | 347.16 (203.90, 535.64) | 89.74 (52.95, 138.74) | -1.40 (-1.74, -1.07) |
| Russian Federation | 23216.44 (21998.22, 24242.83) | 155.24 (146.77, 162.26) | 7897.04 (7120.44, 8652.34) | 34.19 (30.85, 37.46) | -6.00 (-6.36, -5.64) |
| Botswana | 30.01 (17.63, 50.47) | 71.15 (41.68, 119.12) | 78.38 (46.13, 126.69) | 75.42 (44.21, 122.67) | 0.30 (0.15, 0.46) |
| Namibia | 43.87 (23.40, 66.08) | 86.84 (45.92, 131.51) | 86.94 (42.50, 141.21) | 85.66 (42.02, 139.42) | -0.35 (-0.65, -0.05) |
| Kiribati | 0.75 (0.42, 1.25) | 28.52 (16.07, 47.31) | 1.59 (0.87, 2.90) | 35.11 (19.08, 63.91) | 0.81 (0.56, 1.06) |
| Bolivia (Plurinational State of) | 234.10 (133.89, 365.01) | 96.45 (55.25, 150.27) | 769.04 (400.06, 1282.78) | 104.03 (54.49, 173.16) | 0.21 (0.16, 0.26) |
| Maldives | 3.99 (2.39, 6.91) | 68.48 (40.80, 119.99) | 12.36 (6.95, 22.37) | 56.35 (31.64, 101.90) | -0.75 (-0.91, -0.59) |
| Grenada | 6.62 (4.51, 8.33) | 96.09 (66.04, 120.83) | 6.43 (5.28, 7.65) | 66.71 (54.66, 79.55) | -0.86 (-1.35, -0.37) |
| Finland | 272.50 (219.26, 333.98) | 39.79 (31.96, 48.77) | 178.15 (135.78, 226.23) | 13.59 (10.41, 17.23) | -3.57 (-3.79, -3.34) |
| Afghanistan | 450.71 (252.33, 786.94) | 82.11 (45.53, 143.12) | 579.32 (331.06, 967.10) | 91.53 (52.32, 152.67) | 0.46 (0.33, 0.59) |
| Switzerland | 319.15 (260.62, 386.13) | 31.77 (25.96, 38.42) | 355.69 (262.94, 460.35) | 20.02 (14.88, 25.88) | -1.68 (-1.87, -1.49) |
| Bangladesh | 2001.56 (1154.67, 3533.34) | 56.45 (32.56, 99.64) | 6088.42 (3458.15, 10365.39) | 52.43 (29.75, 89.08) | -0.35 (-0.41, -0.30) |
| Solomon Islands | 2.59 (0.77, 7.09) | 28.85 (8.52, 78.23) | 8.22 (2.26, 21.18) | 35.96 (9.87, 92.76) | 0.65 (0.53, 0.77) |
